# The quaternion-based spatial-coordinate and orientation-frame alignment problems

**DOI:** 10.1107/S2053273320002648

**Published:** 2020-06-18

**Authors:** Andrew J. Hanson

**Affiliations:** a Luddy School of Informatics, Computing, and Engineering, Indiana University, Bloomington, Indiana, USA

**Keywords:** data alignment, spatial-coordinate alignment, orientation-frame alignment, quaternions, quaternion frames, quaternion eigenvalue methods

## Abstract

The general problem of finding a global rotation that transforms a given set of spatial coordinates and/or orientation frames (the ‘test’ data) into the best possible alignment with a corresponding set (the ‘reference’ data) is reviewed. For 3D point data, this ‘orthogonal Procrustes problem’ is often phrased in terms of minimizing a root-mean-square deviation (RMSD) corresponding to a Euclidean distance measure relating the two sets of matched coordinates. This article focuses on quaternion eigensystem methods that have been exploited to solve this problem for at least five decades in several different bodies of scientific literature, where they were discovered independently. While numerical methods for the eigenvalue solutions dominate much of this literature, it has long been realized that the quaternion-based RMSD optimization problem can also be solved using exact algebraic expressions based on the form of the quartic equation solution published by Cardano in 1545; focusing on these exact solutions exposes the structure of the entire eigensystem for the traditional 3D spatial-alignment problem. The structure of the less-studied orientation-data context is then explored, investigating how quaternion methods can be extended to solve the corresponding 3D quaternion orientation-frame alignment (QFA) problem, noting the interesting equivalence of this problem to the rotation-averaging problem, which also has been the subject of independent literature threads. The article concludes with a brief discussion of the combined 3D translation–orientation data alignment problem. Appendices are devoted to a tutorial on quaternion frames, a related quaternion technique for extracting quaternions from rotation matrices and a review of quaternion rotation-averaging methods relevant to the orientation-frame alignment problem. The supporting information covers novel extensions of quaternion methods to the 4D Euclidean spatial-coordinate alignment and 4D orientation-frame alignment problems, some miscellaneous topics, and additional details of the quartic algebraic eigenvalue problem.

## Abstract 2

Quaternion methods for obtaining solutions to the problem of finding global rotations that optimally align pairs of corresponding lists of 3D spatial and/or orientation data are critically studied. The existence of multiple literatures and historical contexts is pointed out, and the algebraic solutions of the quaternion approach to the classic 3D spatial problem are emphasized. The treatment is extended to novel quaternion-based solutions to the alignment problems for 4D spatial and orientation data.

## 1. Context   

Aligning matched sets of spatial point data is a universal problem that occurs in a wide variety of applications. In addition, generic objects such as protein residues, parts of composite object models, satellites, cameras, or camera-calibrating reference objects are not only located at points in three-dimensional space, but may also need 3D orientation frames to describe them effectively for certain applications. We are therefore led to consider both the Euclidean spatial-coordinate alignment problem and the orientation-frame alignment problem on the same footing.

Our purpose in this article is to review, and in some cases to refine, clarify and extend, the possible quaternion-based approaches to the optimal alignment problem for matched sets of translated and/or rotated objects in 3D space, which could be referred to in its most generic sense as the ‘generalized orthogonal Procrustes problem’ (Golub & van Loan, 1983 ▸). We also devote some attention to identifying the surprising breadth of domains and literature where the various approaches, including particularly quaternion-based methods, have appeared; in fact the number of times in which quaternion-related methods have been described independently without cross-disciplinary references is rather interesting, and exposes some challenging issues that scientists, including the author, have faced in coping with the wide dispersion of both historical and modern scientific literature relevant to these subjects.

We present our study on two levels. The first level, the present main article, is devoted to a description of the 3D spatial and orientation alignment problems, emphasizing quaternion methods, with an historical perspective and a moderate level of technical detail that strives to be accessible. The second level, comprising the supporting information, treats novel extensions of the quaternion method to the 4D spatial and orientation alignment problems, along with many other technical topics, including analysis of algebraic quartic eigenvalue solutions and numerical studies of the applicability of certain common approximations and methods.

In the following, we first review the diverse bodies of literature regarding the extraction of 3D rotations that optimally align matched pairs of Euclidean point data sets. It is important for us to remark that we have repeatedly become aware of additional literature in the course of this work and it is entirely possible that other worthy references have been overlooked; if so, we apologize for any oversights and hope that the literature that we have found to review will provide an adequate context for the interested reader. We then introduce our own preferred version of the quaternion approach to the spatial-alignment problem, often described as the root-mean-square-deviation (RMSD) minimization problem, and we will adopt that terminology when convenient; our intent is to consolidate a range of distinct variants in the literature into one uniform treatment, and, given the wide variations in symbolic notation and terminology, here we will adopt terms and conventions that work well for us personally. Following a technical introduction to quaternions, we treat the quaternion-based 3D spatial-alignment problem itself. Next we introduce the quaternion approach to the 3D orientation-frame alignment (QFA) problem in a way that parallels the 3D spatial problem, and note its equivalence to quaternion-frame averaging methods. We conclude with a brief analysis of the 6-degree-of-freedom (6DOF) problem, combining the 3D spatial and 3D orientation-frame measures. Appendices include treatments of the basics of quaternion orientation frames, an elegant method that extracts a quaternion from a numerical 3D rotation matrix and the generalization of that method to compute averages of rotations.

## 2. Summary of spatial-alignment problems, known solutions and historical contexts   

### 2.1. The problem, standard solutions and the quaternion method   

The fundamental problem we will be concerned with arises when we are given a well behaved *D* × *D* matrix *E* and we wish to find the optimal *D*-dimensional proper orthogonal matrix 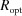 that maximizes the measure 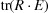. This is equivalent to the RMSD problem, which seeks a global rotation *R* that rotates an ordered set of point test data *X* in such a way as to minimize the squared Euclidean differences relative to a matched reference set *Y*. We will find below that *E* corresponds to the cross-covariance matrix of the pair (*X*, *Y*) of *N* columns of *D*-dimensional vectors, namely 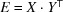, though we will look at cases where *E* could have almost any origin.

One solution to this problem in any dimension *D* uses the decomposition of the general matrix *E* into an orthogonal matrix *U* and a symmetric matrix *S* that takes the form *E* = 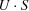 = 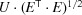, giving 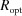 = 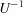 = 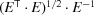; note that there exist several equivalent forms [see, *e.g.*, Green (1952 ▸) and Horn *et al.* (1988 ▸)]. General solutions may also be found using singular-value-decomposition (SVD) methods, starting with the decomposition 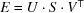, where *S* is now diagonal and *U* and *V* are orthogonal matrices, to give the result 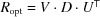, where *D* is the identity matrix up to a possible sign in one element [see, *e.g.*, Kabsch (1976 ▸, 1978 ▸), Golub & van Loan (1983 ▸) and Markley (1988 ▸)].

In addition to these general methods based on traditional linear algebra approaches, a significant literature exists for three dimensions that exploits the relationship between 3D rotation matrices and quaternions, and rephrases the task of finding 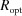 as a *quaternion eigensystem* problem. This approach notes that, using the quadratic quaternion form *R*(*q*) for the rotation matrix, one can rewrite 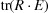 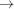 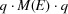, where the *profile matrix* *M*(*E*) is a traceless, symmetric 4 × 4 matrix consisting of linear combinations of the elements of the 3 × 3 matrix *E*. Finding the largest eigenvalue 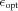 of *M*(*E*) determines the optimal quaternion eigenvector 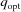 and thus the solution 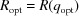. The quaternion framework will be our main topic here.

### 2.2. Historical literature overview   

Although our focus is the quaternion eigensystem context, we first note that one of the original approaches to the RMSD task exploited the singular-value decomposition directly to obtain an optimal rotation matrix. This solution appears to date at least from 1966 in Schönemann’s thesis (Schönemann, 1966 ▸) and possibly Cliff (1966 ▸) later in the same journal issue; Schönemann’s work is chosen for citation, for example, in the earliest editions of Golub & van Loan (1983 ▸). Applications of the SVD to alignment in the aerospace literature appear, for example, in the context of Wahba’s problem (Wikipedia, 2018*b* ▸; Wahba, 1965 ▸) and are used explicitly, *e.g.*, in Markley (1988 ▸), while the introduction of the SVD for the alignment problem in molecular chemistry is generally attributed to Kabsch (Wikipedia, 2018*a* ▸; Kabsch, 1976 ▸), and in machine vision Arun *et al.* (1987 ▸) is often cited.

We believe that the quaternion eigenvalue approach itself was first noticed around 1968 by Davenport (Davenport, 1968 ▸) in the context of Wahba’s problem, rediscovered in 1983 by Hebert and Faugeras (Hebert, 1983 ▸; Faugeras & Hebert, 1983 ▸, 1986 ▸) in the context of machine vision, and then found independently a third time in 1986 by Horn (Horn, 1987 ▸).

An alternative quaternion-free approach by Horn *et al.* (1988 ▸) with the optimal rotation of the form 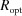 = 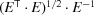 appeared in 1988, but this basic form was apparently known elsewhere as early as 1952 (Green, 1952 ▸; Gibson, 1960 ▸).

**Figure 1:**
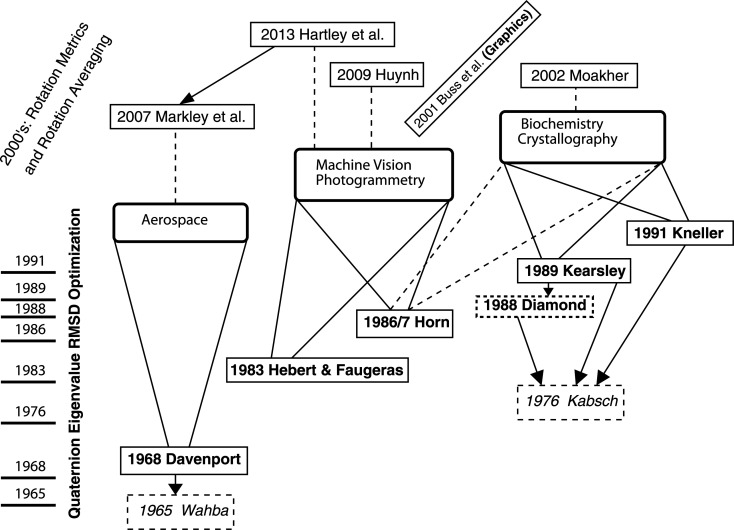
The quaternion eigensystem method for computing the optimal rotation matching two spatial data sets was discovered independently and published without cross-references in at least three distinct literatures. Downward arrows point to the introduction of the abstract problem and upward rays indicate domains of publications specifically citing the quaternion method. Horn eventually appeared routinely in the crystallography citations, and reviews such as that by Flower (1999 ▸) introduced multiple cross-field citations. Several fields have included activity on quaternion-related rotation metrics and rotation averaging with varying degrees of cross-field awareness.

Much of the recent activity has occurred in the context of the molecular alignment problem, starting from a basic framework put forth by Kabsch (1976 ▸, 1978 ▸). So far as we can determine, the matrix eigenvalue approach to molecular alignment was introduced in 1988 without actually mentioning quaternions by name in Diamond (1988 ▸) and refined to specifically incorporate quaternion methods in 1989 by Kearsley (1989 ▸). In 1991 Kneller (Kneller, 1991 ▸) independently described a version of the quaternion-eigenvalue-based approach that is widely cited as well. A concise and useful review can be found in Flower (1999 ▸), in which the contributions of Schönemann, Faugeras and Hebert, Horn, Diamond, and Kearsley are acknowledged and all cited in the same place. A graphical summary of the discovery chronology in various domains is given in Fig. 1 ▸. Most of these treatments mention using numerical methods to find the optimal eigenvalue, though several references, starting with Horn (1987 ▸), point out that 16th-century algebraic methods for solving the quartic polynomial characteristic equation, discussed in the next section, could also be used to determine the eigenvalues. In our treatment we will study the explicit form of these algebraic solutions for the 3D problem (and also for 4D in the supporting information), taking advantage of several threads of the literature.

### 2.3. Historical notes on the quartic   

The actual solution to the quartic equation, and thus the solution of the characteristic polynomial of the 4D eigensystem of interest to us, was first published in 1545 by Gerolamo Cardano (Wikipedia, 2019 ▸) in his book *Ars Magna*. The intellectual history of this fact is controversial and narrated with varying emphasis in diverse sources. It seems generally agreed upon that Cardano’s student Lodovico Ferrari was the first to discover the basic method for solving the quartic in 1540, but his technique was incomplete as it only reduced the problem to the cubic equation, for which no solution was publicly known at that time, and that apparently prevented him from publishing it. The complication appears to be that Cardano had actually learned of a method for solving the cubic already in 1539 from Niccolò Fontana Tartaglia (legendarily in the form of a poem), but had been sworn to secrecy, and so could not reveal the final explicit step needed to complete Ferrari’s implicit solution. Where it gets controversial is that at some point between 1539 and 1545, Cardano learned that Scipione del Ferro had found the same cubic solution as the one of Tartaglia that he had sworn not to reveal, and furthermore that del Ferro had discovered his solution before Tartaglia did. Cardano interpreted that fact as releasing him from his oath of secrecy (which Tartaglia did not appreciate), allowing him to publish the complete solution to the quartic, incorporating the cubic solution into Ferrari’s result. Sources claiming that Cardano ‘stole’ Ferrari’s solution may perhaps be exaggerated, since Ferrari did not have access to the cubic equations and Cardano did not conceal his sources; exactly who ‘solved’ the quartic is thus philosophically complicated, but Cardano does seem to be the one who combined the multiple threads needed to express the equations as a single complete formula.

Other interesting observations were made later, for example by Descartes in 1637 (Descartes, 1637 ▸) and in 1733 by Euler (Euler, 1733 ▸; Bell, 2008 ▸). For further descriptions, one may consult, *e.g.*, Abramowitz & Stegun (1970 ▸) and Boyer & Merzbach (1991 ▸), as well as the narratives in Weisstein (2019*a* ▸,*b* ▸). Additional amusing pedagogical investigations of the historical solutions may be found in several expositions by Nickalls (1993 ▸, 2009 ▸).

### 2.4. Further literature   

A very informative treatment of the features of the quaternion eigenvalue solutions was given by Coutsias, Seok and Dill in 2004, and expanded in 2019 (Coutsias *et al.*, 2004 ▸; Coutsias & Wester, 2019 ▸). Coutsias *et al.* not only take on a thorough review of the quaternion RMSD method, but also derive the complete relationship between the linear algebra of the SVD method and the quaternion eigenvalue system; furthermore, they exhaustively enumerate the special cases involving mirror geometries and degenerate eigenvalues that may appear rarely, but must be dealt with on occasion. Efficiency is also an area of potential interest, and Theobald *et al.* in Theobald (2005 ▸) and in Liu *et al.* (2010 ▸) argue that among the many variants of numerical methods that have been used to compute the optimal quaternion eigenvalues, Horn’s original proposal to use Newton’s method directly on the characteristic equations of the relevant eigenvalue systems may well be the best approach.

There is also a rich literature dealing with distance measures among representations of rotation frames themselves, some dealing directly with the properties of distances computed with rotation matrices or quaternions, *e.g.* Huynh (2009 ▸), and others combining discussion of the distance measures with associated applications such as rotation averaging or finding ‘rotational centers of mass’, *e.g.* Brown & Worsey (1992 ▸), Park & Ravani (1997 ▸), Buss & Fillmore (2001 ▸), Moakher (2002 ▸), Markley *et al.* (2007 ▸), and Hartley *et al.* (2013 ▸). The specific computations explored below in Section 7 on optimal alignment of matched pairs of orientation frames make extensive use of the quaternion-based and rotation-based measures discussed in these treatments. In the appendices, we review the details of some of these orientation-frame-based applications.

## 3. Introduction   

We explore the problem of finding global rotations that optimally align pairs of corresponding lists of spatial and/or orientation data. This issue is significant in diverse application domains. Among these are aligning spacecraft (see, *e.g.*, Wahba, 1965 ▸; Davenport, 1968 ▸; Markley, 1988 ▸; and Markley & Mortari, 2000 ▸), obtaining correspondence of registration points in 3D model matching (see, *e.g.*, Faugeras & Hebert, 1983 ▸, 1986 ▸), matching structures in aerial imagery (see, *e.g.*, Horn, 1987 ▸; Horn *et al.*, 1988 ▸; Huang *et al.*, 1986 ▸; Arun *et al.*, 1987 ▸; Umeyama, 1991 ▸; and Zhang, 2000 ▸), and alignment of matched molecular and biochemical structures (see, *e.g.*, Kabsch, 1976 ▸, 1978 ▸; McLachlan, 1982 ▸; Lesk, 1986 ▸; Diamond, 1988 ▸; Kearsley, 1989 ▸, 1990 ▸; Kneller, 1991 ▸; Coutsias *et al.*, 2004 ▸; Theobald, 2005 ▸; Liu *et al.*, 2010 ▸; and Coutsias & Wester, 2019 ▸). A closely related task is the alignment of multiple sets of 3D range data, for example in digital-heritage applications (Levoy *et al.*, 2000 ▸); the widely used iterative closest point (ICP) algorithm [see, *e.g.*, Chen & Medioni (1991 ▸), Besl & McKay (1992 ▸) and Bergevin *et al.* (1996 ▸), as well as Rusinkiewicz & Levoy (2001 ▸) and Nüchter *et al.* (2007 ▸)] explicitly incorporates standard alignment methods in individual steps with known correspondences.

We note in particular the several alternative approaches of Schönemann (1966 ▸), Faugeras & Hebert (1983 ▸), Horn (1987 ▸) and Horn *et al.* (1988 ▸) that in principle produce the same optimal global rotation to solve a given alignment problem. While the SVD and 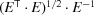 methods apply to any dimension, here we will critically examine the quaternion eigensystem decomposition approach that applies only to the 3D and 4D spatial-coordinate alignment problems, along with the extensions to the 3D and 4D orientation-frame alignment problems. Starting from the quartic algebraic equations for the quaternion eigensystem arising in our optimization problem, we direct attention to the elegant exact algebraic forms of the eigenvalue solutions appropriate for these applications. (For brevity, the more complicated 4D treatment is deferred to the supporting information.)

Our extension of the quaternion approach to *orientation data* exploits the fact that collections of 3D orientation frames can *themselves* be expressed as quaternions, *e.g.* amino-acid 3D orientation frames written as quaternions (see Hanson & Thakur, 2012 ▸), and we will refer to the corresponding ‘quaternion-frame alignment’ task as the QFA problem. Various proximity measures for such orientation data have been explored in the literature (see, *e.g.*, Park & Ravani, 1997 ▸; Moakher, 2002 ▸; Huynh, 2009 ▸; and Huggins, 2014*a* ▸), and the general consensus is that the most rigorous measure minimizes the sums of squares of geodesic arc lengths between pairs of quaternions. This ideal QFA proximity measure is highly nonlinear compared to the analogous spatial RMSD measure, but fortunately there is an often-justifiable linearization, the chord angular distance measure. We present several alternative solutions exploiting this approximation that closely parallel our spatial RMSD formulation, and point out the relationship to the rotation-averaging problem.

In addition, we analyze the problem of optimally aligning *combined *3D spatial and quaternion 3D-frame-triad data, a 6DOF task that is relevant to studying molecules with composite structure as well as to some gaming and robotics contexts. Such rotational–translational measures have appeared in both the computer vision and the molecular entropy literature [see, *e.g.*, the dual-quaternion approach of Walker *et al.* (1991 ▸) as well as Huggins (2014*b* ▸) and Fogolari *et al.* (2016 ▸)]; after some confusion, it has been recognized that the spatial and rotational measures are dimensionally incompatible, and either they must be optimized independently, or an arbitrary context-dependent scaling parameter with the dimension of length must appear in any combined measure for the RMSD+QFA problem.

In the following, we organize our thoughts by first summarizing the fundamentals of quaternions, which will be our main computational tool. We next introduce the measures that underlie the general spatial alignment problem, then restrict our attention to the quaternion approach to the 3D problem, emphasizing a class of exact algebraic solutions that can be used as an alternative to the traditional numerical methods. Our quaternion approach to the 3D orientation-frame triad alignment problem is presented next, along with a discussion of the combined spatial–rotational problem. Appendices provide an alternative formulation of the 3D RMSD optimization equations, a tutorial on the quaternion orientation-frame methodology, and a summary of the method of Bar-Itzhack (2000 ▸) for obtaining the corresponding quaternion from a numerical 3D rotation matrix, along with a treatment of the closely related quaternion-based rotation averaging problem.

In the supporting information, we extend all of our 3D results to 4D space, exploiting quaternion pairs to formulate the 4D spatial-coordinate RMSD alignment and 4D orientation-based QFA methods. We expose the relationship between these quaternion methods and the singular-value decomposition, and extend the 3D Bar-Itzhack approach to 4D, showing how to find the pair of quaternions corresponding to any numerical 4D rotation matrix. Other sections of the supporting information explore properties of the RMSD problem for 2D data and evaluate the accuracy of our 3D orientation-frame alignment approximations, as well as studying and evaluating the properties of combined measures for aligning spatial-coordinate and orientation-frame data in 3D. An appendix is devoted to further details of the quartic equations and forms of the algebraic solutions related to our eigenvalue problems.

## 4. Foundations of quaternions   

For the purposes of this paper, we take a quaternion to be a point *q* = (*q*_0_, *q*_1_, *q*_2_, *q*_3_) = (*q*_0_, **q**) in 4D Euclidean space with unit norm, *q* · *q* = 1, and so geometrically it is a point on the unit 3-sphere **S** ^3^ [see, *e.g.*, Hanson (2006 ▸) for further details about quaternions]. The first term, *q*_0_, plays the role of a real number and the last three terms, denoted as a 3D vector **q**, play the role of a generalized imaginary number, and so are treated differently from the first: in particular the conjugation operation is taken to be 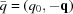. Quaternions possess a multiplication operation denoted by 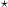 and defined as follows:
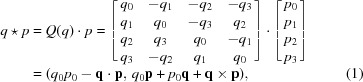
where the orthonormal matrix *Q*(*q*) expresses a form of quaternion multiplication that can be useful. Note that the orthonormality of *Q*(*q*) means that quaternion multiplication of *p* by *q literally* produces a rotation of *p* in 4D Euclidean space.

Choosing exactly one of the three imaginary components in both *q* and *p* to be nonzero gives back the classic complex algebra (*q*_0_ + i*q*_1_)(*p*_0_ + i*p*_1_) = (*q*_0_ *p*_0_ − *q*_1_ *p*_1_) + i(*q*_0_ *p*_1_ + *p*_0_ *q*_1_), so there are three copies of the complex numbers embedded in the quaternion algebra; the difference is that in general the final term **q** × **p** changes sign if one reverses the order, making the quaternion product order-dependent, unlike the complex product. Nevertheless, like complex numbers, the quaternion algebra satisfies the non-trivial ‘multiplicative norm’ relation
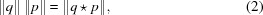
where 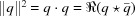, *i.e.* quaternions are one of the four possible Hurwitz algebras (real, complex, quaternion and octonion).

Quaternion triple products obey generalizations of the 3D vector identities *A* · (*B* × *C*) = *B* · (*C* × *A*) = *C* · (*A* × *B*), along with *A* × *B* = −*B* × *A*. The corresponding quaternion identities, which we will need in Section 7, are
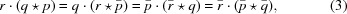
where the complex-conjugate entries are the natural consequences of the sign changes occurring only in the 3D part.

It can be shown that quadratically conjugating a vector **x** = (*x*, *y*, *z*), written as a purely ‘imaginary’ quaternion (0, **x**) (with only a 3D part), by quaternion multiplication is isomorphic to the construction of a 3D Euclidean rotation *R*(*q*) generating all possible elements of the special orthogonal group **SO**(3). If we compute
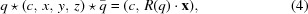
we see that only the purely imaginary part is affected, whether or not the arbitrary real constant *c* = 0. The result of collecting coefficients of the vector term is a *proper* orthonormal 3D rotation matrix quadratic in the quaternion elements that takes the form
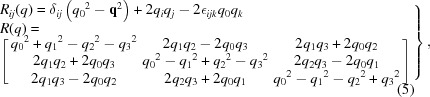
with determinant 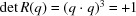. The formula for *R*(*q*) is technically a two-to-one mapping from quaternion space to the 3D rotation group because *R*(*q*) = *R*(−*q*); changing the sign of the quaternion preserves the rotation matrix. Note also that the identity quaternion 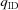 = 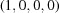 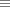 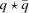 corresponds to the identity rotation matrix, as does 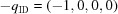. The 3 × 3 matrix *R*(*q*) is fundamental not only to the quaternion formulation of the spatial RMSD alignment problem, but will also be essential to the QFA orientation-frame problem because the *columns* of *R*(*q*) are exactly the needed quaternion representation of the *frame triad* describing the orientation of a body in 3D space, *i.e.* the columns are the vectors of the frame’s local *x*, *y* and *z* axes relative to an initial identity frame.

Multiplying a quaternion *p* by the quaternion *q* to get a new quaternion 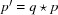 simply *rotates* the 3D frame corresponding to *p* by the matrix equation (5) written in terms of *q*. This has non-trivial implications for 3D rotation matrices, for which quaternion multiplication corresponds exactly to multiplication of two *independent* 3 × 3 orthogonal rotation matrices, and we find that
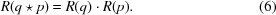
This collapse of repeated rotation matrices to a single rotation matrix with multiplied quaternion arguments can be continued indefinitely.

If we choose the following specific 3-variable parameterization of the quaternion *q* preserving *q* · *q* = 1,
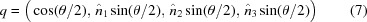
(with 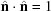), then 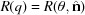 is precisely the ‘axis-angle’ 3D spatial rotation by an angle θ leaving the direction 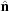 fixed, so 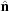 is the lone real eigenvector of *R*(*q*).

### 4.1. The slerp   

Relationships among quaternions can be studied using the *slerp*, or ‘spherical linear interpolation’ (Shoemake, 1985 ▸; Jupp & Kent, 1987 ▸), which smoothly parameterizes the points on the shortest geodesic quaternion path between two constant (unit) quaternions, *q*_0_ and *q*_1_, as
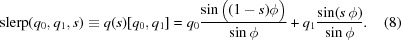
Here 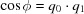 defines the angle ϕ between the two given quaternions, while *q*(*s* = 0) = *q*_0_ and *q*(*s* = 1) = *q*_1_. The ‘long’ geodesic can be obtained for 1 ≤ *s* ≤ 2π/ϕ. For small ϕ, this reduces to the standard linear interpolation (1 − *s*)*q*_0_ + *s* *q*_1_. The unit norm is preserved, *q*(*s*) · *q*(*s*) = 1 for all *s*, so *q*(*s*) is always a valid quaternion and *R*(*q*(*s*)) defined by equation (5) is always a valid 3D rotation matrix. We note that one can formally write equation (8) as an exponential of the form 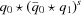, but since this requires computing a logarithm and an exponential whose most efficient reduction to a practical computer program is equation (8), this is mostly of pedagogical interest.

In the following we will make little further use of the quaternion’s algebraic properties, but we will extensively exploit equation (5) to formulate elegant approaches to RMSD problems, along with employing equation (8) to study the behavior of our data under smooth variations of rotation matrices.

### 4.2. Remark on 4D   

Our fundamental formula equation (5) can be extended to four Euclidean dimensions by choosing two *distinct* quaternions in equation (4), producing a 4D Euclidean rotation matrix. Analogously to 3D, the columns of this matrix correspond to the axes of a 4D Euclidean orientation frame. The non-trivial details of the quaternion approach to aligning both 4D spatial-coordinate and 4D orientation-frame data are given in the supporting information.

## 5. Reviewing the 3D spatial-alignment RMSD problem   

We now review the basic ideas of spatial data alignment, and then specialize to 3D (see, *e.g.*, Wahba, 1965 ▸; Davenport, 1968 ▸; Markley, 1988 ▸; Markley & Mortari, 2000 ▸; Kabsch, 1976 ▸, 1978 ▸; McLachlan, 1982 ▸; Lesk, 1986 ▸; Faugeras & Hebert, 1983 ▸; Horn, 1987 ▸; Huang *et al.*, 1986 ▸; Arun *et al.*, 1987 ▸; Diamond, 1988 ▸; Kearsley, 1989 ▸, 1990 ▸; Umeyama, 1991 ▸; Kneller, 1991 ▸; Coutsias *et al.*, 2004 ▸; and Theobald, 2005 ▸). We will then employ quaternion methods to reduce the 3D spatial-alignment problem to the task of finding the optimal quaternion eigenvalue of a certain 4 × 4 matrix. This is the approach we have discussed in the introduction, and it can be solved using numerical or algebraic eigensystem methods. In Section 6 below, we will explore in particular the classical quartic equation solutions for the exact algebraic form of the entire four-part eigensystem, whose optimal eigenvalue and its quaternion eigenvector produce the optimal global rotation solving the 3D spatial-alignment problem.

### 5.1. Aligning matched data sets in Euclidean space   

We begin with the general least-squares form of the RMSD problem, which is solved by minimizing the optimization measure over the space of rotations, which we will convert to an optimization over the space of unit quaternions. We take as input one data array with *N* columns of *D*-dimensional points {*y*_*k*_} as the *reference* structure and a second array of *N* columns of *matched* points {*x*_*k*_} as the *test* structure. Our task is to rotate the latter in space by a global **SO**(*D*) rotation matrix *R*_*D*_ to achieve the minimum value of the cumulative quadratic distance measure
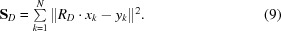
We assume, as is customary, that any overall translational components have been eliminated by displacing both data sets to their centers of mass (see, *e.g.*, Faugeras & Hebert, 1983 ▸; Coutsias *et al.*, 2004 ▸). When this measure is minimized with respect to the rotation *R*_*D*_, the optimal *R*_*D*_ will rotate the test set {*x*_*k*_} to be as close as possible to the reference set {*y*_*k*_}. Here we will focus on 3D data sets (and, in the supporting information, 4D data sets) because those are the dimensions that are easily adaptable to our targeted quaternion approach. In 3D, our least-squares measure equation (9) can be converted directly into a quaternion optimization problem using the method of Hebert and Faugeras detailed in Appendix A[App appa].

*Remark:* Clifford algebras may support alternative methods as well as other approaches to higher dimensions [see, *e.g.*, Havel & Najfeld (1994 ▸) and Buchholz & Sommer (2005 ▸)].

### 5.2. Converting from least-squares minimization to cross-term maximization   

We choose from here onward to focus on an equivalent method based on expanding the measure given in equation (9), removing the constant terms, and recasting the RMSD least-squares minimization problem as the task of maximizing the surviving cross-term expression. This takes the general form
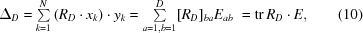
where
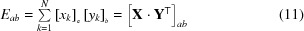
is the *cross-covariance matrix* of the data, [*x*_*k*_] denotes the *k*th column of **X** and the range of the indices (*a*, *b*) is the spatial dimension *D*.

### 5.3. Quaternion transformation of the 3D cross-term form   

We now restrict our attention to the *3D cross-term form* of equation (10) with pairs of 3D point data related by a proper rotation. The key step is to substitute equation (5) for *R*(*q*) into equation (10) and pull out the terms corresponding to pairs of components of the quaternions *q*. In this way the 3D expression is transformed into the 4 × 4 matrix *M*(*E*) sandwiched between two identical quaternions (not a conjugate pair) of the form
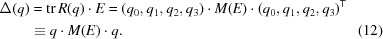
Here *M*(*E*) is the traceless, symmetric 4 × 4 matrix
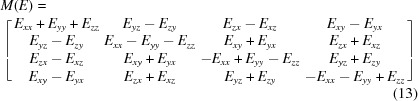
built from our original 3 × 3 cross-covariance matrix *E* defined by equation (11). We will refer to *M*(*E*) from here on as the *profile matrix*, as it essentially reveals a different viewpoint of the optimization function and its relationship to the matrix *E*. Note that in some literature matrices related to the cross-covariance matrix *E* may be referred to as ‘attitude profile matrices’ and one also may see the term ‘key matrix’ referring to *M*(*E*).

The bottom line is that if one decomposes equation (13) into its eigensystem, the measure equation (12) is maximized when the unit-length quaternion vector *q* is the eigenvector of *M*(*E*)’s largest eigenvalue (Davenport, 1968 ▸; Faugeras & Hebert, 1983 ▸; Horn, 1987 ▸; Diamond, 1988 ▸; Kearsley, 1989 ▸; Kneller, 1991 ▸). The RMSD optimal-rotation problem thus reduces to finding the maximal eigenvalue 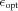 of *M*(*E*) (which we emphasize depends only on the numerical data). Plugging the corresponding eigenvector 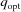 into equation (5), we obtain the rotation matrix 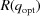 that solves the problem. The resulting proximity measure relating {*x*_*k*_} and {*y*_*k*_} is simply
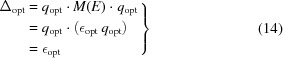
and does not require us to actually compute 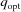 or 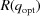 explicitly if all we want to do is compare various test data sets to a reference structure.

*Note*. In the interests of conceptual and notational simplicity, we have made a number of assumptions. For one thing, in declaring that equation (5) describes our sought-for rotation matrix, we have presumed that the optimal rotation matrix will always be a proper rotation, with 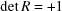. Also, as mentioned, we have omitted any general translation problems, assuming that there is a way to translate each data set to an appropriate center, *e.g.* by subtracting the center of mass. The global translation optimization process is treated in Faugeras & Hebert (1986 ▸) and Coutsias *et al.* (2004 ▸), and discussions of center-of-mass alignment, scaling and point weighting are given in much of the original literature, see, *e.g.*, Horn (1987 ▸), Coutsias *et al.* (2004 ▸), and Theobald (2005 ▸). Finally, in real problems, structures such as molecules may appear in mirror-image or enantiomer form, and such issues were introduced early on by Kabsch (1976 ▸, 1978 ▸). There can also be particular symmetries, or very close approximations to symmetries, that can make some of our natural assumptions about the good behavior of the profile matrix invalid, and many of these issues, including ways to treat degenerate cases, have been carefully studied [see, *e.g.*, Coutsias *et al.* (2004 ▸) and Coutsias & Wester (2019 ▸)]. The latter authors also point out that if a particular data set *M*(*E*) produces a negative smallest eigenvalue ∊_4_ such that 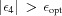, this can be a sign of a reflected match, and the *negative* rotation matrix 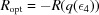 may actually produce the best alignment. These considerations may be essential in some applications, and readers are referred to the original literature for details.

### 5.4. Illustrative example   

**Figure 2:**
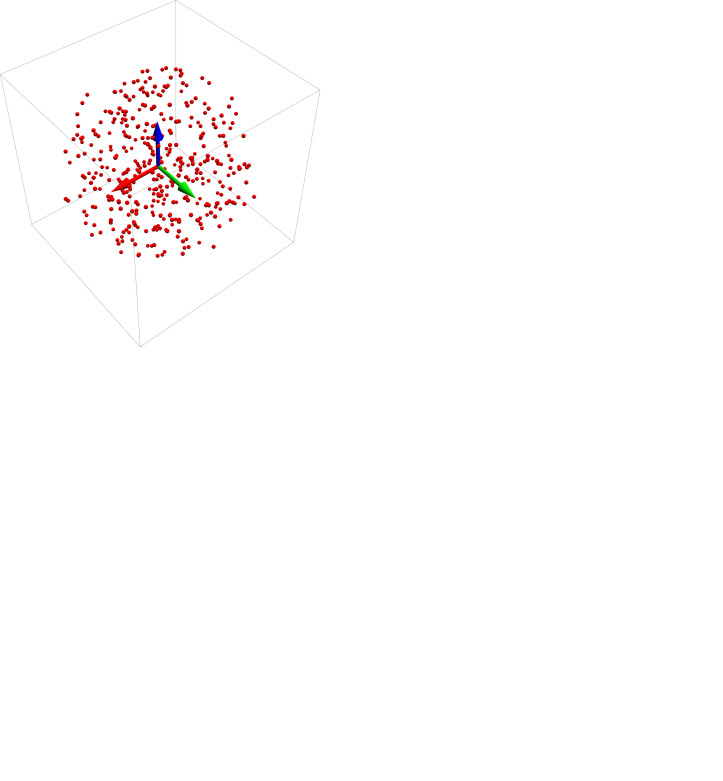
(*a*) A typical 3D spatial reference data set. (*b*) The reference data in red alongside the test data in blue, with blue lines representing the Euclidean distances connecting each test data point with its corresponding reference point. (*c*) The partial alignment at *s* = 0.75. (*d*) The optimal alignment for this data set at *s* = 1.0. The yellow arrow is the axis of rotation specified by the optimal quaternion’s spatial components.

**Figure 3:**
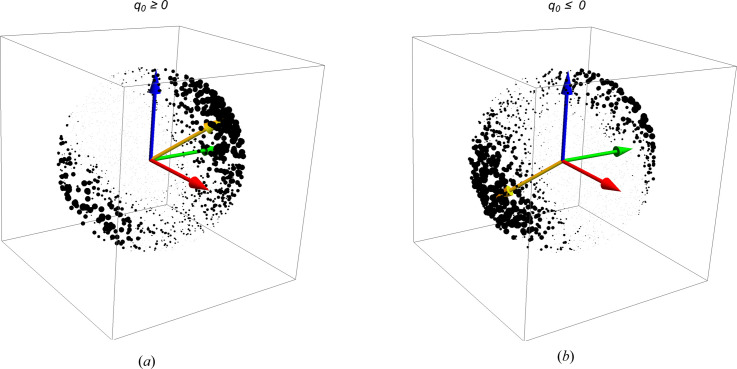
The values of 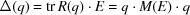 represented by the sizes of the dots placed randomly in the ‘northern’ and ‘southern’ 3D solid balls spanning the entire hypersphere **S** ^3^ with (*a*) containing the *q*_0_ ≥ 0 sector and (*b*) containing the *q*_0_ ≤ 0 sector. We display the data dots at the locations of their spatial quaternion components **q** = (*q*_1_, *q*_2_, *q*_3_), and we know that *q*_0_ = ±(1 − **q** · **q**)^1/2^ so the **q** data uniquely specify the full quaternion. Since *R*(*q*) = *R*(−*q*), the points in *each* ball actually represent all possible unique rotation matrices. The spatial component of the maximal eigenvector is shown by the yellow arrows, which clearly end in the middle of the maximum values of Δ(*q*). Note that, in the quaternion context, diametrically opposite points on the spherical surface are identical rotations, so the cluster of larger dots at the upper right of (*a*) is, in the entire sphere, representing the same data as the ‘diametrically opposite’ lower-left cluster in (*b*), both surrounding the tips of their own yellow arrows. The smaller dots at the upper right of (*b*) are contiguous with the upper-right region of (*a*), forming a single cloud centered on 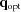, and similarly for the lower left of (*a*) and the lower left of (*b*). The whole figure contains two distinct clusters of dots (related by *q* → −*q*) centered around 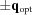.

We can visualize the transition from the initial data 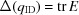 to the optimal alignment 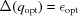 by exploiting the geodesic interpolation equation (8) from the identity quaternion 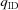 to 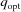 given by
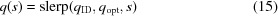
and applying the resulting rotation matrix *R*(*q*(*s*)) to the test data, ending with 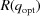 showing the best alignment of the two data sets. In Fig. 2 ▸, we show a sample reference data set in red, a sample test data set in blue connected to the reference data set by blue lines, an intermediate partial alignment and finally the optimally aligned pair. The yellow arrow is the *spatial part of the quaternion solution*, proportional to the eigenvector 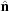 (fixed axis) of the optimal 3D rotation matrix 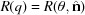, and whose length is 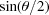, sine of half the rotation angle needed to perform the optimal alignment of the test data with the reference data. In Fig. 3 ▸, we visualize the optimization process in an alternative way, showing random samples of *q* = (*q*_0_, **q**) in **S** ^3^, separated into the ‘northern hemisphere’ 3D unit-radius ball in (*a*) with *q*_0_ ≥ 0, and the ‘southern hemisphere’ 3D unit-radius ball in (*b*) with *q*_0_ ≤ 0. (This is like representing the Earth as two flattened discs, one showing everything above the equator and one showing everything below the equator; the distance from the equatorial plane is implied by the location in the disc, with the maximum at the centers, the north and south poles.) Either solid ball contains one unique quaternion for every possible choice of *R*(*q*), modulo the doubling of diametrically opposite points at *q*_0_ = 0. The values of 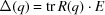 are shown as scaled dots located at their corresponding spatial (‘imaginary’) quaternion points **q** in the solid balls. The yellow arrows, equivalent negatives of each other, show the spatial part 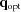 of the optimal quaternion 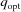, and the tips of the arrows clearly fall in the middle of the mirror pair of clusters of the largest values of Δ(*q*). Note that the lower-left dots in (*a*) continue smoothly into the larger lower-left dots in (*b*), which is the center of the optimal quaternion in (*b*). Further details of such methods of displaying quaternions are provided in Appendix B[App appb] [see also Hanson (2006 ▸)].

## 6. Algebraic solution of the eigensystem for 3D spatial alignment   

At this point, one can simply use the traditional *numerical* methods to solve equation (12) for the maximal eigenvalue 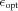 of *M*(*E*) and its eigenvector 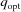, thus solving the 3D spatial-alignment problem of equation (10). Alternatively, we can also exploit *symbolic* methods to study the properties of the eigensystems of 4 × 4 matrices *M* algebraically to provide deeper insights into the structure of the problem, and that is the subject of this section.

Theoretically, the algebraic form of our eigensystem is a textbook problem following from the 16th-century-era solution of the quartic algebraic equation in, *e.g.*, Abramowitz & Stegun (1970 ▸). Our objective here is to explore this textbook solution in the specific context of its application to eigensystems of 4 × 4 matrices and its behavior relative to the properties of such matrices. The real, symmetric, traceless profile matrix *M*(*E*) in equation (13) appearing in the 3D spatial RMSD optimization problem must necessarily possess only real eigenvalues, and the properties of *M*(*E*) permit some particular simplifications in the algebraic solutions that we will discuss. The quaternion RMSD literature varies widely in the details of its treatment of the algebraic solutions, ranging from no discussion at all, to Horn, who mentions the possibility but does not explore it, to the work of Coutsias *et al.* (Coutsias *et al.*, 2004 ▸; Coutsias & Wester, 2019 ▸), who present an exhaustive treatment, in addition to working out the exact details of the correspondence between the SVD eigensystem and the quaternion eigensystem, both of which in principle embody the algebraic solution to the RMSD optimization problem. In addition to the treatment of Coutsias *et al.*, other approaches similar to the one we will study are due to Euler (Euler, 1733 ▸; Bell, 2008 ▸), as well as a series of papers on the quartic by Nickalls (1993 ▸, 2009 ▸).

### 6.1. Eigenvalue expressions   

We begin by writing down the eigenvalue expansion of the profile matrix,
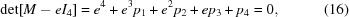
where *e* denotes a generic eigenvalue, *I*_4_ is the 4D identity matrix and the *p*_*k*_ are homogeneous polynomials of degree *k* in the elements of *M*. For the special case of a traceless, symmetric profile matrix *M*(*E*) defined by equation (13), the *p*_*k*_(*E*) coefficients simplify and can be expressed numerically as the following functions either of *M* or of *E*:
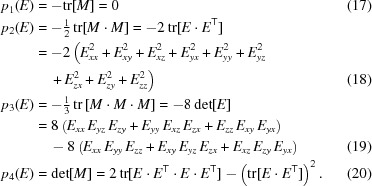
Interestingly, the polynomial *M*(*E*) is arranged so that −*p*_2_(*E*)/2 is the (squared) Fröbenius norm of *E*, and −*p*_3_(*E*)/8 is its determinant. Our task now is to express the four eigenvalues *e* = ∊_*k*_(*p*_1_, *p*_2_, *p*_3_, *p*_4_), *k* = 1, …, 4, usefully in terms of the matrix elements, and also to find their eigenvectors; we are of course particularly interested in the maximal eigenvalue 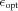.

### 6.2. Approaches to algebraic solutions   

Equation (16) can be solved directly using the quartic equations published by Cardano in 1545 [see, *e.g.*, Abramowitz & Stegun (1970 ▸), Weisstein (2019*b* ▸), and Wikipedia (2019 ▸)], which are incorporated into the *Mathematica* function
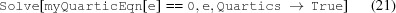
that immediately returns a suitable algebraic formula. At this point we defer detailed discussion of the textbook solution to the supporting information, and instead focus on a particularly symmetric version of the solution and the form it takes for the eigenvalue problem for traceless, symmetric 4 × 4 matrices such as our profile matrices *M*(*E*). For this purpose, we look for an alternative solution by considering the following traceless (*p*_1_ = 0) ansatz:
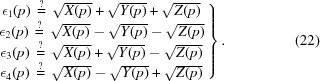
 This form emphasizes some additional explicit symmetry that we will see is connected to the role of cube roots in the quartic algebraic solutions (see, *e.g.*, Coutsias & Wester, 2019 ▸). We can turn it into an equation for ∊_*k*_(*p*) to be solved in terms of the matrix parameters *p*_*k*_(*E*) as follows: First we eliminate *e* using (*e* − ∊_1_)(*e* − ∊_2_)(*e* − ∊_3_)(*e* − ∊_4_) = 0 to express the matrix data expressions *p*_*k*_ directly in terms of totally symmetric polynomials of the eigenvalues in the form (Abramowitz & Stegun, 1970 ▸)
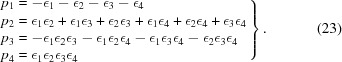
Next we substitute our expression equation (22) for the ∊_*k*_ in terms of the {*X*, *Y*, *Z*} functions into equation (23), yielding a completely different alternative to equation (16) that will *also* solve the 3D RMSD eigenvalue problem if we can invert it to express {*X*(*p*), *Y*(*p*), *Z*(*p*)} in terms of the data *p*_*k*_(*E*) as presented in equation (20):
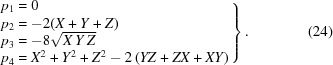
We already see the critical property in *p*_3_ that, while *p*_3_ itself has a deterministic sign from the matrix data, the possibly variable signs of the square roots in equation (22) have to be constrained so their product 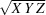 agrees with the sign of *p*_3_. Manipulating the quartic equation solutions that we can obtain by applying the library function equation (21) to equation (24), and restricting our domain to real traceless, symmetric matrices (and hence real eigenvalues), we find solutions for *X*(*p*), *Y*(*p*) and *Z*(*p*) of the following form:
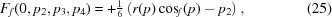
where the 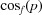 terms differ only by a cube-root phase:
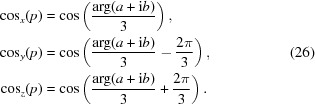
Here 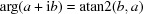 in the C mathematics library, or ArcTan[*a*, *b*] in *Mathematica*, *F*_*f*_(*p*) with *f* = (*x*, *y*, *z*) corresponds to *X*(*p*), *Y*(*p*) or *Z*(*p*), and the utility functions appearing in the equations for our traceless *p*_1_ = 0 case are
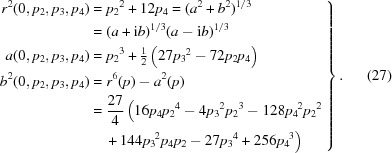
The function *b* ^2^(*p*) has the essential property that, for real solutions to the cubic, which imply the required real solutions to our eigenvalue equations (Abramowitz & Stegun, 1970 ▸), we must have *b* ^2^(*p*) ≥ 0. That essential property allowed us to convert the bare solution into terms involving {(*a* + i*b*)^1/3^, (*a* − i*b*)^1/3^} whose sums form the manifestly real cube-root-related cosine terms in equation (26).

### 6.3. Final eigenvalue algorithm   

While equations (25) and (26) are well defined, square roots must be taken to finish the computation of the eigenvalues postulated in equation (22). In our special case of symmetric, traceless matrices such as *M*(*E*), we can always choose the signs of the first two square roots to be positive, but the sign of the 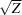 term is non-trivial, and in fact is the sign of 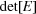. The form of the solution in equations (22) and (25) that works specifically for all traceless symmetric matrices such as *M*(*E*) is given by our equations for *p*_*k*_(*E*) in equations (17)–(22), along with equations (25), (26) and (27) *provided* we modify equation (22) using 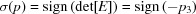 as follows:
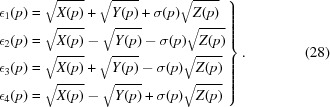
The particular order of the numerical eigenvalues in our chosen form of the solution equation (28) is found in regular cases to be uniformly non-increasing in numerical order for our *M*(*E*) matrices, so ∊_1_(*p*) is always the leading eigenvalue. This is our preferred symbolic version of the solution to the 3D RMSD problem defined by *M*(*E*).

*Note*: We have experimentally confirmed the numerical behavior of equation (25) in equation (28) with 1 000 000 randomly generated sets of 3D cross-covariance matrices *E*, along with the corresponding profile matrices *M*(*E*), producing numerical values of *p*_*k*_ inserted into the equations for *X*(*p*), *Y*(*p*) and *Z*(*p*). We confirmed that the sign of σ(*p*) varied randomly, and found that the algebraically computed values of ∊_*k*_(*p*) corresponded to the standard numerical eigenvalues of the matrices *M*(*E*) in all cases, to within expected variations due to numerical evaluation behavior and expected occasional instabilities. In particular, we found a maximum per-eigenvalue discrepancy of about 10^−13^ for the *algebraic* methods relative to the standard *numerical* eigenvalue methods, and a median difference of 10^−15^, in the context of machine precision of about 10^−16^. (Why did we do this? Because we had earlier versions of the algebraic formulas that produced anomalies due to inconsistent phase choices in the roots, and felt it worthwhile to perform a practical check on the numerical behavior of our final version of the solutions.)

### 6.4. Eigenvectors for 3D data   

The eigenvector formulas corresponding to ∊_*k*_ can be generically computed by solving any three rows of
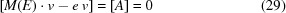
for the elements of *v*, *e.g.* *v* = (1, *v*_1_, *v*_2_, *v*_3_), as a function of some eigenvalue *e* (of course, one must account for special cases, *e.g.* if some subspace of *M*(*E*) is already diagonal). The desired unit quaternion for the optimization problem can then be obtained from the normalized eigenvector
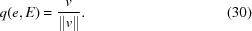
Note that this can often have *q*_0_ < 0, and that whenever the problem in question depends on the sign of *q*_0_, such as a slerp starting at 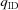, one should choose the sign of equation (30) appropriately; some applications may also require an element of statistical randomness, in which case one might randomly pick a sign for *q*_0_.

As noted by Liu *et al.* (2010 ▸), a very clear way of computing the eigenvectors for a given eigenvalue is to exploit the fact that the determinant of equation (29) must vanish, that is 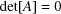; one simply exploits the fact that the columns of the adjugate matrix α_*ij*_ (the transpose of the matrix of cofactors of the matrix [*A*]) produce its inverse by means of creating multiple copies of the determinant. That is,
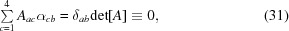
so we can just compute *any* column of the adjugate via the appropriate set of subdeterminants and, in the absence of singularities, that will be an eigenvector (since any of the four columns can be eigenvectors, if one fails just try another).

In the general well behaved case, the form of *v* in the eigenvector solution for any eigenvalue *e* = ∊_*k*_ may be explicitly computed to give the corresponding quaternion (among several equivalent alternative expressions) as
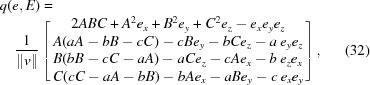
where for convenience we define {*e*_*x*_ = (*e* − *x* + *y* + *z*), *e*_*y*_ = (*e* + *x* − *y* + *z*), *e*_*z*_ = (*e* + *x* + *y* − *z*)} with *x* = *E*_*xx*_, cyclic, *a* = *E*_*yz*_ − *E*_*zy*_, cyclic, and *A* = *E*_*yz*_ + *E*_*zy*_, cyclic. We substitute the maximal eigenvector 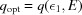 into equation (5) to give the sought-for optimal 3D rotation matrix 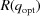 that solves the RMSD problem with 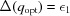, as we noted in equation (14).

*Remark*: Yet another approach to computing eigenvectors that, surprisingly, *almost* entirely avoids any reference to the original matrix, but needs only its eigenvalues and minor eigenvalues, has recently been rescued from relative obscurity (Denton *et al.*, 2019 ▸). (The authors uncovered a long list of non-cross-citing literature mentioning the result dating back at least to 1934.) If, for a real, symmetric 4 × 4 matrix *M* we label the set of four eigenvectors *v*_*i*_ by the index *i* and the components of any single such four-vector by *a*, the squares of each of the sixteen corresponding components take the form 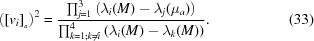
Here the μ_*a*_ are the 3 × 3 minors obtained by removing the *a*th row and column of *M*, and the λ_*j*_(μ_*a*_) comprise the list of three eigenvalues of each of these minors. Attempting to obtain the eigenvectors by taking square roots is of course hampered by the nondeterministic sign; however, since the eigenvalues λ_*i*_(*M*) are known, and the overall sign of each eigenvector *v*_*i*_ is arbitrary, one needs to check at most eight sign combinations to find the one for which *M* · *v*_*i*_ = λ_*i*_(*M*)*v*_*i*_, solving the problem. Note that the general formula extends to Hermitian matrices of any dimension.

## 7. The 3D orientation-frame alignment problem   

We turn next to the orientation-frame problem, assuming that the data are like lists of orientations of rollercoaster cars, or lists of residue orientations in a protein, ordered pairwise in some way, but *without* specifically considering any spatial location or nearest-neighbor ordering information. In *D*-dimensional space, the *columns* of any **SO**(*D*) orthonormal *D* × *D* rotation matrix *R*_*D*_ are what we mean by an orientation frame, since these columns are the directions pointed to by the axes of the identity matrix after rotating something from its defining identity frame to a new attitude; note that no spatial location information whatever is contained in *R*_*D*_, though one may wish to choose a local center for each frame if the construction involves coordinates such as amino-acid atom locations (see, *e.g.*, Hanson & Thakur, 2012 ▸).

In 2D, 3D and 4D, there exist two-to-one quadratic maps from the topological spaces **S** ^1^, **S** ^3^ and **S** ^3^ × **S** ^3^ to the rotation matrices *R*_2_, *R*_3_ and *R*_4_. These are the quaternion-related objects that we will use to obtain elegant representations of the frame data-alignment problem. In 2D, a frame data element can be expressed as a complex phase, while in 3D the frame is a unit quaternion [see Hanson (2006 ▸) and Hanson & Thakur (2012 ▸)]. In 4D (see the supporting information), the frame is described by a *pair* of unit quaternions.

*Note*. Readers unfamiliar with the use of complex numbers and quaternions to obtain elegant representations of 2D and 3D orientation frames are encouraged to review the tutorial in Appendix B[App appb].

### 7.1. Overview   

We focus now on the problem of aligning corresponding sets of 3D orientation frames, just as we already studied the alignment of sets of 3D spatial coordinates by performing an optimal rotation. There will be more than one feasible method. We might assume we could just define the quaternion-frame alignment or ‘QFA’ problem by converting any list of frame orientation matrices to quaternions [see Hanson (2006 ▸), Hanson & Thakur (2012 ▸) and also Appendix C[App appc]] and writing down the quaternion equivalents of the RMSD treatment in equation (9) and equation (10). However, unlike the linear Euclidean problem, the preferred quaternion optimization function technically requires a *nonlinear* minimization of the squared sums of geodesic arc lengths connecting the points on the quaternion hypersphere **S** ^3^. The task of formulating this ideal problem as well as studying alternative approximations is the subject of its own branch of the literature, often known as the *quaternionic barycenter* problem or the *quaternion averaging* problem (see, *e.g.*, Brown & Worsey, 1992 ▸; Buss & Fillmore, 2001 ▸; Moakher, 2002 ▸; Markley *et al.*, 2007 ▸; Huynh, 2009 ▸; Hartley *et al.*, 2013 ▸; and also Appendix D[App appd]). We will focus on *L*_2_ norms (the aformentioned sums of squares of arc lengths), although alternative approaches to the rotation-averaging problem, such as employing *L*_1_ norms and using the Weiszfeld algorithm to find the optimal rotation numerically, have been advocated, *e.g.*, by Hartley *et al.* (2011 ▸). The computation of optimally aligning rotations, based on plausible exact or approximate measures relating collections of corresponding pairs of (quaternionic) orientation frames, is now our task.

Choices for the forms of the measures encoding the distance between orientation frames have been widely discussed, see, *e.g.*, Park & Ravani (1997 ▸), Moakher (2002 ▸), Markley *et al.* (2007 ▸), Huynh (2009 ▸), Hartley *et al.* (2011 ▸, 2013 ▸), and Huggins (2014*a* ▸). Since we are dealing primarily with quaternions, we will start with two measures dealing directly with the quaternion geometry, the geodesic arc length and the chord length, and later on examine some advantages of starting with quaternion-sign-independent rotation-matrix forms.

### 7.2. 3D geodesic arc-length distance   

**Figure 4:**
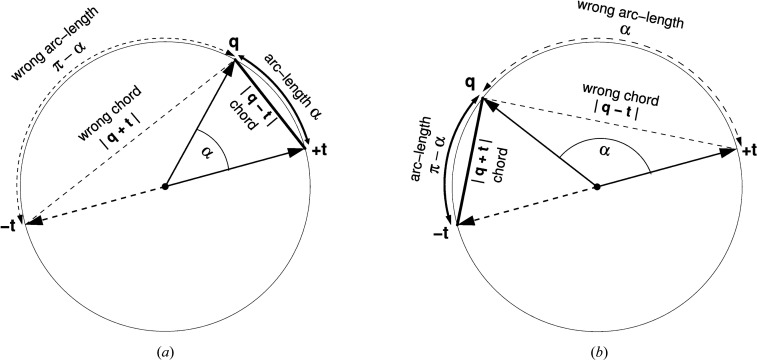
Geometric context involved in choosing a *quaternion distance* that will result in the correct *average rotation matrix* when the quaternion measures are optimized. Because the quaternion vectors represented by *t* and −*t* give the same rotation matrix, one must choose 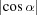 or the *minima*, that is 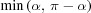 or 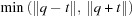, of the alternative distance measures to get the *correct* items in the arc-length or chord measure summations. (*a*) and (*b*) represent the cases when the first or second choice should be made, respectively.

First, we recall that the matrix equation (5) has three orthonormal columns that define a quadratic map from the quaternion three-sphere **S** ^3^, a smooth connected Riemannian manifold, to a 3D orientation frame. The squared geodesic arc-length distance between two quaternions lying on the three-sphere **S** ^3^ is generally agreed upon as the measure of orientation-frame proximity whose properties are the closest in principle to the ordinary squared Euclidean distance measure equation (9) between points (Huynh, 2009 ▸), and we will adopt this measure as our starting point. We begin by writing down a frame–frame distance measure between two unit quaternions *q*_1_ and *q*_2_, corresponding precisely to two orientation frames defined by the columns of *R*(*q*_1_) and *R*(*q*_2_). We define the geodesic arc length as an angle α on the hypersphere **S** ^3^ computed geometrically from 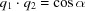. As pointed out by Huynh (2009 ▸) and Hartley *et al.* (2013 ▸), the geodesic arc length between a test quaternion *q*_1_ and a data-point quaternion *q*_2_ of ambiguous sign [since *R*(+*q*_2_) = *R*(−*q*_2_)] can take two values, and we want the minimum value. Furthermore, to work on a spherical manifold instead of a plane, we need basically to cluster the ambiguous points in a deterministic way. Starting with the bare angle between two quaternions on **S** ^3^, 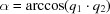, where we recall that α ≥ 0, we define a *pseudometric* (Huynh, 2009 ▸) for the geodesic arc-length distance as
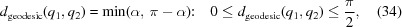
as illustrated in Fig. 4 ▸. An efficient implementation of this is to take
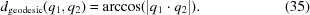
We now seek to define an ideal minimizing *L*_2_ orientation-frame measure, comparable to our minimizing Euclidean RMSD measure, but constructed from geodesic arc lengths on the quaternion hypersphere instead of Euclidean distances in space. Thus to compare a test quaternion-frame data set {*p*_*k*_} to a reference data set {*r*_*k*_}, we propose the geodesic based least-squares measure
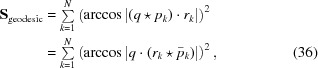
where we have used the identities of equation (3). When 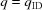, the individual measures correspond to equation (35), and otherwise ‘
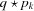
’ is the exact analog of ‘*R*(*q*) · *x*_*k*_’ in equation (9), and denotes the quaternion rotation *q* acting on the entire set {*p*_*k*_} to rotate it to a new orientation that we want to align optimally with the reference frames {*r*_*k*_}. Analogously, for points on a sphere, the arccosine of an inner product is equivalent to a distance between points in Euclidean space.

*Remark:* For improved numerical behavior in the computation of the quaternion inner-product angle between two quaternions, one may prefer to convert the arccosine to an arctangent form, 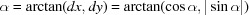 [remember the C math library uses the opposite argument order atan2(*dy*, *dx*)], with the parameters
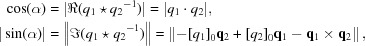
which is somewhat more stable.

### 7.3. Adopting the solvable chord measure   

Unfortunately, the geodesic arc-length measure does not fit into the linear algebra approach that we were able to use to obtain exact solutions for the Euclidean-data-alignment problem treated so far. Thus we are led to investigate instead a very close approximation to 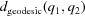 that *does* correspond closely to the Euclidean data case and does, with some contingencies, admit exact solutions. This approximate measure is the *chord distance*, whose individual distance terms analogous to equation (35) take the form of a closely related pseudometric (Huynh, 2009 ▸; Hartley *et al.*, 2013 ▸),
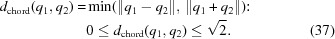
We compare the geometric origins for equation (35) and equation (37) in Fig. 4 ▸. Note that the crossover point between the two expressions in equation (37) is at π/2, so the hypotenuse of the right isosceles triangle at that point has length 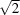.

The solvable approximate optimization function analogous to *∥R* · *x* − *y∥* ^2^ that we will now explore for the quaternion-frame alignment problem will thus take the form that must be minimized as
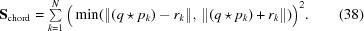
We can convert the sign ambiguity in equation (38) to a deterministic form like equation (35) by observing, with the help of Fig. 4 ▸, that
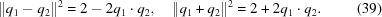
Clearly (2 − 2|*q*_1_ · *q*_2_|) is always the smallest of the two values. Thus minimizing equation (38) amounts to maximizing the now-familiar cross-term form, which we can write as
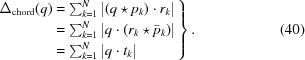
Here we have used the identity 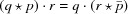 from equation (3) and defined the quaternion displacement or ‘attitude error’ (Markley *et al.*, 2007 ▸)
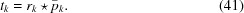
Note that we could have derived the same result using equation (2) to show that 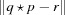 = 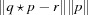 = 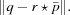


There are several ways to proceed to our final result at this point. The simplest is to pick a neighborhood in which we will choose the samples of *q* that include our expected optimal quaternion, and adjust the sign of each data value *t*_*k*_ to 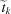 by the transformation
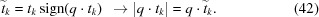
The neighborhood of *q* matters because, as argued by Hartley *et al.* (2013 ▸), even though the allowed range of 3D rotation angles is θ ∈ (−π, π) [or quaternion sphere angles α ∈ (−π/2, π/2)], convexity of the optimization problem cannot be guaranteed for collections outside local regions centered on some θ_0_ of size θ_0_ ∈ (−π/2, π/2) [or α_0_ ∈ (−π/4, π/4)]: beyond this range, local basins may exist that allow the mapping equation (42) to produce distinct local variations in the assignments of the 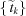 and in the solutions for 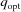. Within considerations of such constraints, equation (42) now allows us to take the summation outside the absolute value, and write the quaternion-frame optimization problem in terms of maximizing the cross-term expression
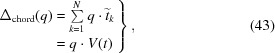
where 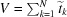 is proportional to the mean of the quaternion displacements 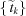, defining their chord-distance *quaternion average*. *V* also clearly plays a role analogous to the Euclidean RMSD profile matrix *M*. However, since equation (43) is *linear* in *q*, we have the remarkable result that, as noted in the treatment of Hartley *et al.* (2013 ▸) regarding the quaternion *L*_2_ chordal-distance norm, the solution is immediate, being simply
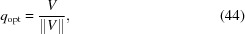
since that obviously maximizes the value of 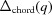. This gives the maximal value of the measure as
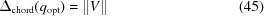
and thus *∥V∥* is the exact orientation-frame analog of the spatial RMSD maximal eigenvalue 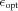, except it is far easier to compute.

### 7.4. Illustrative example   

**Figure 5:**
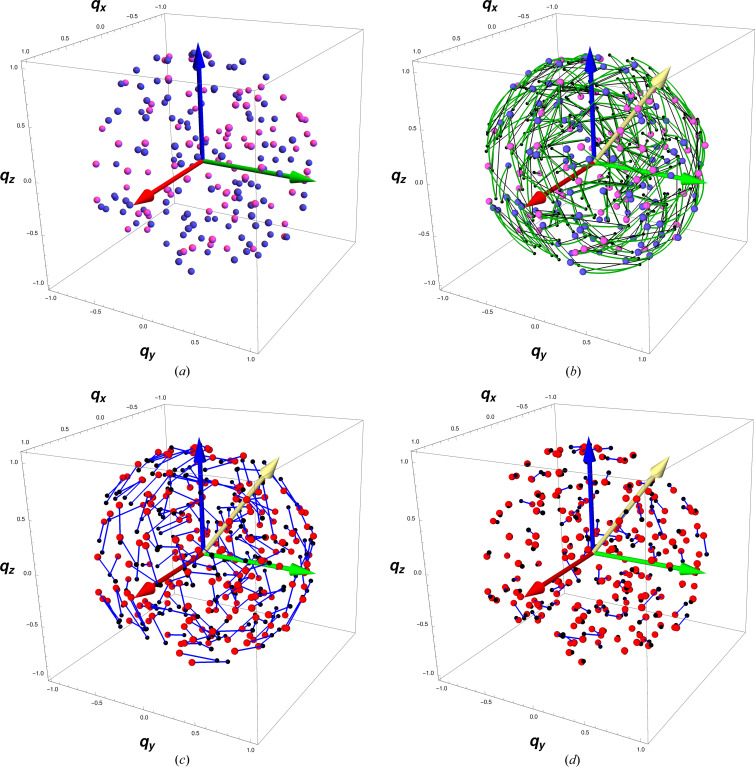
3D components of a quaternion orientation data set. (*a*) A quaternion reference set, color-coded by the sign of *q*_0_. (*b*) *Exact* quaternion arc-length distances (green arcs) versus chord distances (black lines) between the test points (black dots) and the reference points. (*c*) Part way from the starting state to the aligned state, at *s* = 0.5. (*d*) The final best alignment at *s* = 1.0. The yellow arrow is the direction of the quaternion eigenvector; when scaled, the length is the sine of half the optimal rotation angle.

Using the quaternion display method described in Appendix B[App appb] and illustrated in Fig. 12, we present in Fig. 5 ▸(*a*) a representative quaternion-frame reference data set, then in (*b*) we include a matching set of rotated noisy test data (small black dots), and draw the arc and chord distances (see also Fig. 4 ▸) connecting each test-reference point pair in the quaternion space. In Fig. 5 ▸(*c*,*d*), we show the results of the quaternion-frame alignment process using conceptually the same slerp of equation (15) to transition from the raw state at 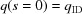 to *q*(*s* = 0.5) for (*c*) and 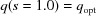 for (*d*). The yellow arrow is the axis of rotation specified by the spatial part of the optimal quaternion.

**Figure 6:**
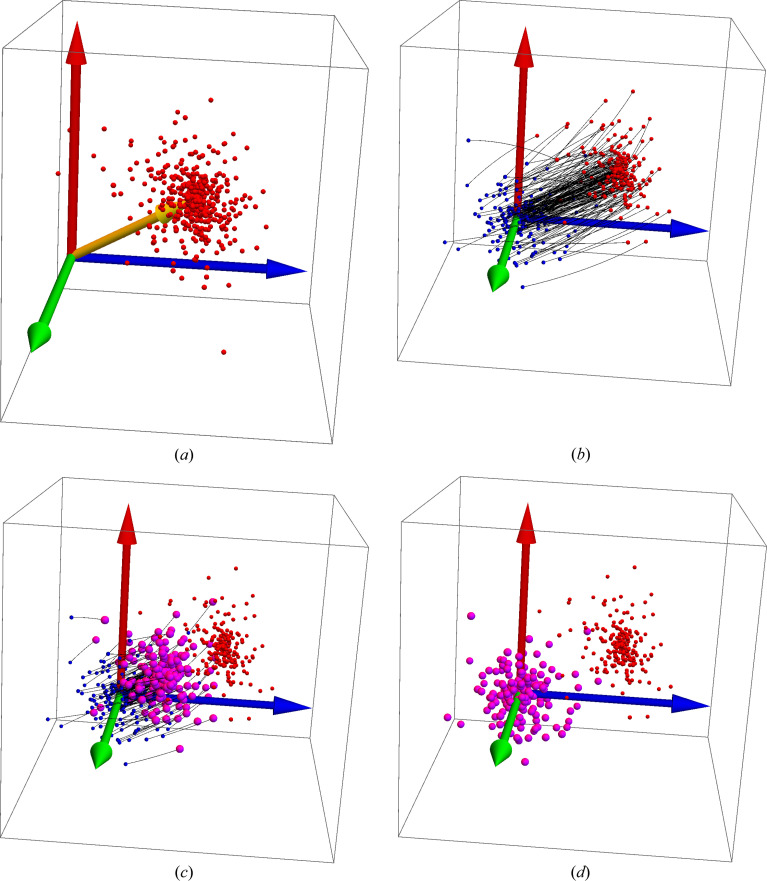
3D components of the rotation-average transformation of the quaternion orientation data set, with each point denoting the *displacement* between each pair of frames as a single quaternion, corresponding to the rotation taking the test frame to the reference frame. (*a*) The cluster of points 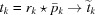 derived from the frame-matching problem using just the curved arcs in Fig. 5 ▸(*b*). If there were no alignment errors introduced in the simulation, these would all be a single point. The yellow arrow is the quaternion solution to the chord-distance centroid of this cluster and is identical to the optimal quaternion rotation transforming the test data to have the minimal chord measure relative to the reference data. (*b*) Choosing a less-cluttered subset of the data in (*a*), we display the geodesic paths from the initial quaternion displacements 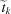 to the origin-centered set with minimal chord-measure distance relative to the origin. This is the result of applying the inverse of the quaternion 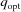 to each 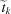. Note that the paths are curved geodesics lying properly within the quaternion sphere. (*c*,*d*) Rotating the cluster using a slerp between the quaternion barycenter of the initial misaligned data and the optimally aligned position, which is centered at the origin.

**Figure 7:**
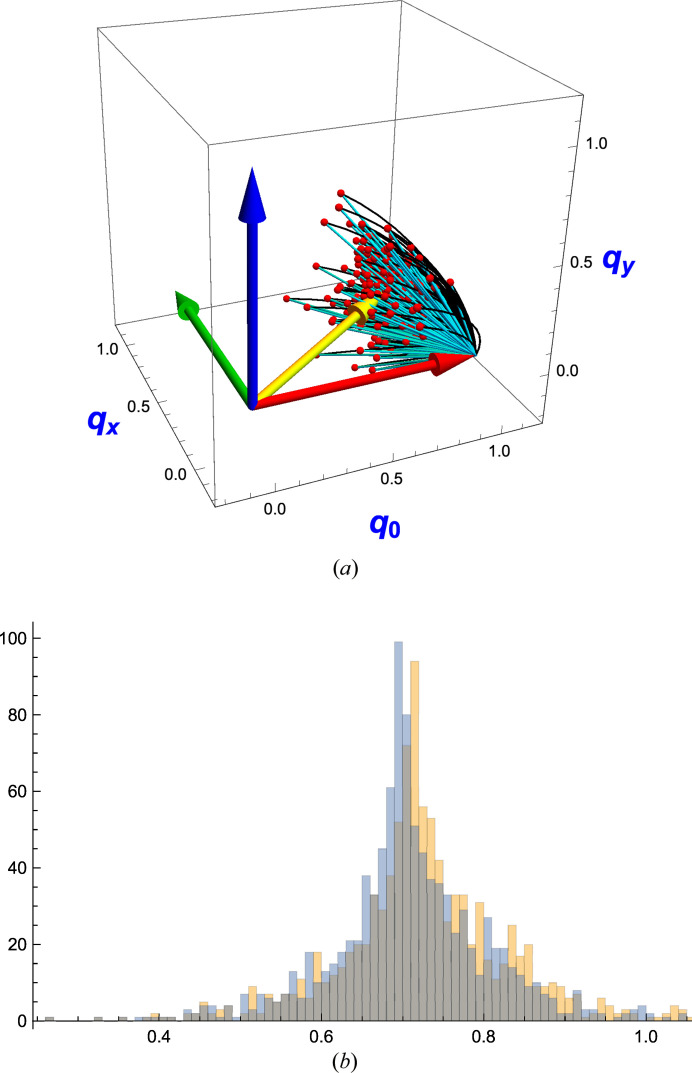
(*a*) Projecting the geodesic versus chord distances from the origin to sampled points in a set of frame-displacement data 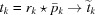. Since the **q** spatial quaternion paths project to a straight line from the origin, we use the (*q*_0_, *q*_*x*_, *q*_*y*_) coordinates instead of our standard **q** coordinates to expose the curvature in the arc-length distances to the origin. (*b*) Histogram of the chord-length distances to the origin (in blue) compared to the histogram of the geodesic arc-length distances (in yellow), sampled using a uniform distribution of random quaternions over a portion of **S** ^3^. If there were no errors, all the points would have the same distance from the origin located at *q* = (1, 0, 0, 0) [red axis in (*a*)], and there would be one blue spike, appearing at a slightly smaller position than the yellow spike because arc length is always longer than chord length. The arc-length method has a different distribution, as expected, and produces a very slightly better barycenter. However, the optimal quaternions for the arc-length versus chord-length measure for this simulated data set differ by only a fraction of a degree, so drawing the positions of the two distinct optimal quaternions would not reveal any noticeable difference in image (*a*).

The rotation-averaging visualization of the optimization process, though it has exactly the same optimal quaternion, is quite different, since all the quaternion data collapse to a list of single small quaternions 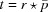. As illustrated in Fig. 6 ▸, with compatible sign choices, the 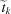
’s cluster around the optimal quaternion, which is clearly consistent with being the barycenter of the quaternion differences, intuitively the place to which all the quaternion frames need to be rotated to optimally coincide. As before, the yellow arrow is the axis of rotation specified by the spatial part of the optimal quaternion. Next, Fig. 7 ▸ addresses the question of how the rigorous arc-length measure is related to the chord-length measure that can be treated using the same methods as the spatial RMSD optimization. In parallel to Fig. 5 ▸(*b*), Fig. 7 ▸(*a*) shows essentially the same comparison for the 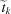 quaternion-displacement version of the same data. In Fig. 7 ▸(*b*), we show the histograms of the chord distances to a sample point, the origin in this case, versus the arc-length or geodesic distances. They obviously differ, but in fact for plausible simulations, the arc-length numerical optimal quaternion barycenter differs from the chord-length counterpart by only a fraction of a degree. These issues are studied in more detail in the supporting information.

**Figure 8:**
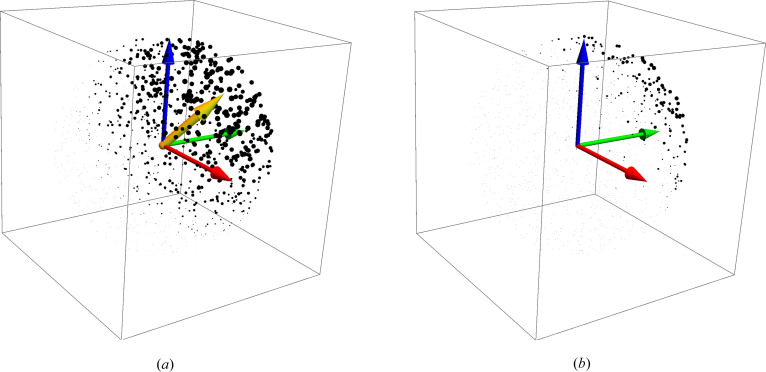
The values of Δ = *q* · *V* represented by the sizes of the dots placed at a random distribution of quaternion points. We display the data dots at the locations of their spatial quaternion components **q**. (*a*) is the northern hemisphere of **S** ^3^, with *q*_0_ ≥ 0, (*b*) is the southern hemisphere, with *q*_0_ ≤ 0, and we implicitly know that the value of *q*_0_ is 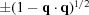. The points in these two solid balls represent the entire space of quaternions, and it is important to note that, even though *R*(*q*) = *R*(−*q*) so each ball alone actually represents all possible unique rotation matrices, our cost function covers the *entire* space of quaternions, so *q* and −*q* are distinct. The spatial component of the maximal eigenvector is shown by the yellow arrow, which clearly ends in the middle of the maximum values of Δ. The small cloud at the edge of (*b*) is simply the rest of the complete cloud around the tip of the yellow arrow, as *q*_0_ passes through the ‘equator’ at *q*_0_ = 0, going from a small positive value at the edge of (*a*) to a small negative value at the edge of (*b*).

**Figure 9:**
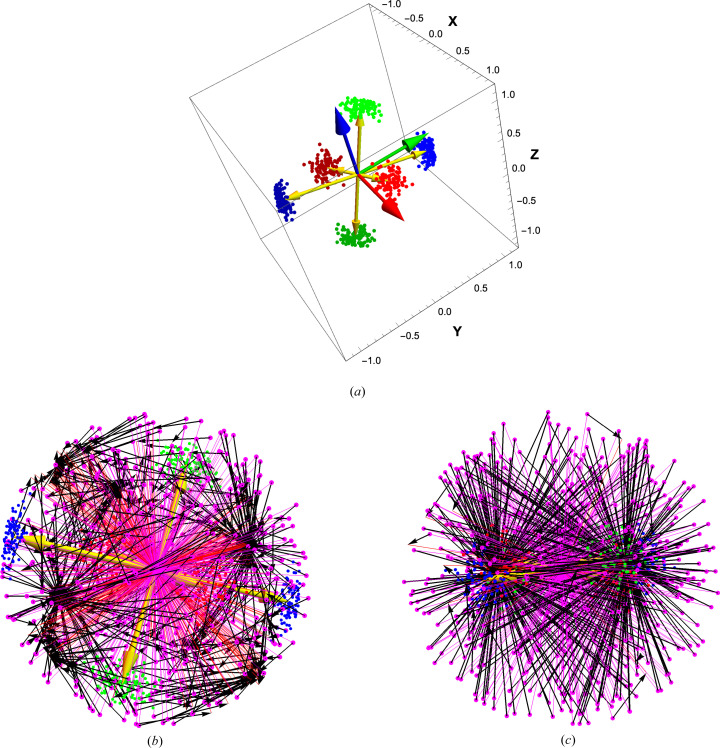
The behavior of the basins of attraction for the 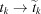 map is shown here, starting in (*a*) with the (*q*, −*q*) pairs for three movable clusters of quaternion-frame data, each having a well defined *local* quaternion average 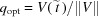 shown as the yellow arrows with their *q* → −*q* equivalents. Next we merge all three samples into one data set that can be smoothly interpolated between the data being outside the α = π/4 safe zone to all being together within that geometric boundary in quaternion space. Part (*b*) shows the results of taking 500 uniform samples of *q* and computing the set 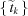 for *each* sample *q*, placed at the magenta dots, and *then* computing the resulting 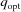; the black arrows follow the line from the sample point to the resultant 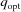. Clearly in (*b*), where the clusters are in their initial widely dispersed configuration, the black arrows (the ‘votes’ for the best 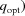 collect in several different basins of attraction, signifying the absence of a global solution. We then interpolate all the clusters close to each other, and show the new results of the voting in (*c*). Now almost all of the samplings of the full quaternion space converge to point their arrows densely to the two opposite values of 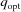, and there is just one effective basin of attraction.

**Figure 12:**
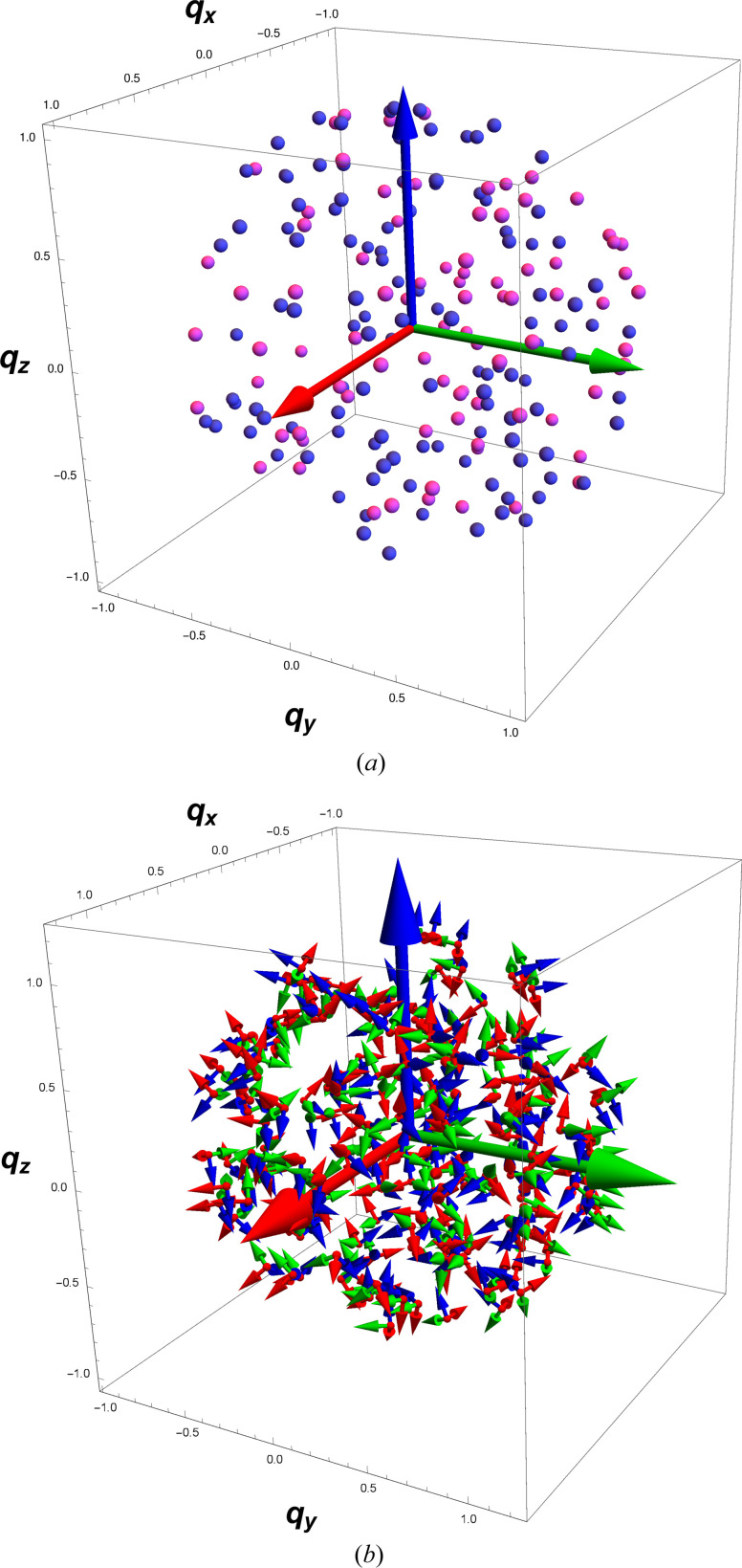
(*a*) The 3D portions of the quaternion reference-frame data *q* = (*q*_0_, *q*_*x*_, *q*_*y*_, *q*_*z*_), using different colors for *q*_0_ ≥ 0 and *q*_0_ < 0 in the unseen direction. Since 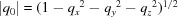, the complete quaternion can in principle be determined from the 3D display. (*b*) The 3D orientation-frame triads for each reference point (*q*_0_, *q*_*x*_, *q*_*y*_, *q*_*z*_) displayed at their associated **q** = (*q*_*x*_, *q*_*y*_, *q*_*z*_).

Next, in Fig. 8 ▸, we display the values of 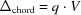 that parallel the RMSD version in Fig. 3 ▸. The dots show the size of the cost Δ(*q*) at randomly sampled points across the entire **S** ^3^, with *q*_0_ ≥ 0 in (*a*) and *q*_0_ ≤ 0 in (*b*). We have all the signs of the 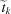 chosen to be centered in an appropriate local neighborhood, and so, unlike the quadratic Euclidean RMSD case, there is only one value for 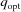, which is in the direction of *V*. Finally, in Fig. 9 ▸ we present an intuitive sketch of the convexity constraints for the QFA optimization related to Hartley *et al.* (2013 ▸). We start with a set of data in (*a*) [with both (*q*, −*q*) partners] that consists of three local clouds that can be smoothly deformed from dispersed to coinciding locations. Fig. 9(*b*) and (*c*) both contain a uniform sample of quaternion sample points *q* spread over all of quaternion space, shown as magenta dots, with positive and negative *q*_0_ plotted on top of each other. Then each sample *q* is used to compute *one* set of mappings 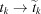 and the *one* value of 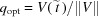 that results. The black arrows show the relation of 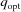 to each original sample *q*, effectively showing us their *votes* for the best quaternion average. Fig. 9(*b*) has the clusters positioned far enough apart that we can clearly see that there are several basins of attraction, with no unique solution for 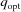, while in (*c*), we have interpolated the three clusters to lie in the same local neighborhood, roughly in a ball of quaternion radius α < π/4, and we see that almost all of the black arrows vote for one unique 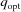 or its equivalent negative. This seems to be a useful exercise to gain intuition about the nature of the basins of attraction for the quaternion-averaging problem that is essential for quaternion-frame alignment.

### 7.5. Alternative matrix forms of the linear vector chord distance   

If the signs of the quaternions representing orientation frames are well behaved, and the frame problem is our only concern, equations (43) and (44) provide a simple solution to finding the optimal global rotation. If we are anticipating wanting to combine a spatial profile matrix *M*(*E*) with an orientation problem in a single 4 × 4 matrix, or we have problems defining a consistent quaternion sign, there are two further choices of orientation-frame measure we may consider.

(1) *Matrix form of the linear vector chord distance*. The first option uses the fact that the square of equation (43) will yield the same extremal solution for *q*_opt_, so we can choose a measure of the form
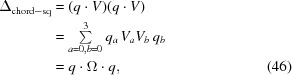
where Ω_*ab*_ = *V*_*a*_ *V*_*b*_ is a 4 × 4 rank-one symmetric matrix with 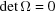, and 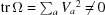. The eigensystem of Ω is just defined by the eigenvalue *∥V∥* ^2^, and combination with the spatial eigensystem can be achieved either numerically or algebraically. The sign issues for the sampled data remain unchanged since they appear inside the sums defining *V*. This form will acquire more importance in the 4D case.

(2) *Fixing the sign problem with the quadratic rotation matrix chord distance*. Our second approach has a very natural way to *eliminate sign dependence altogether* from the quaternion chord-distance method, and has a close relationship to 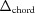. This measure is constructed starting from a minimized Fröbenius norm of the form [this approach is used by Sarlette & Sepulchre (2009 ▸); see also, *e.g.*, Huynh (2009 ▸), as well as Moakher (2002 ▸), Markley *et al.* (2007 ▸), and Hartley *et al.* (2013 ▸)]
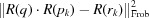
and then reducing to the cross-term as usual. The cross-term measure to be maximized, in terms of 3 × 3 (quaternion-sign-independent) rotation matrices, then becomes
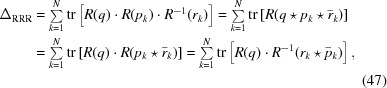
where 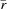 denotes the complex conjugate or inverse quaternion. We can verify that this is a chord distance by noting that each relevant *R* · *R* · *R* term reduces to the square of an individual chord distance appearing in 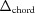:
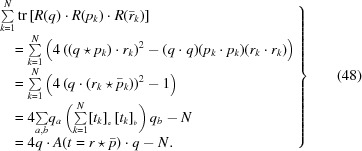
Here the non-conjugated ordinary *r* on the right-hand side is not a typographical error, and the 4 × 4 matrix *A*(*t*) is the alternative (equivalent) profile matrix that was introduced by Markley *et al.* (2007 ▸) and Hartley *et al.* (2013 ▸) for the chord-based quaternion-averaging problem. We can therefore use either the measure 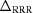 or
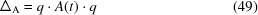
with 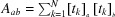 as our rotation-matrix-based sign-insensitive chord-distance optimization measure. Exactly like our usual spatial measure, these measures must be *maximized* to find the optimal *q*. It is, however, important to emphasize that the optimal quaternion will *differ* for the 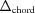, 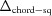 and 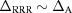 measures, though they will normally be very similar (see the discussion in the supporting information).

We now recognize that the sign-insensitive measures are all very closely related to our original spatial RMSD problem, and all can be solved by finding the optimal quaternion eigenvector 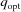 of a 4 × 4 matrix. The procedure for 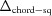 and 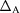 follows immediately, but it is useful to work out the options for 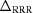 in a little more detail. Defining 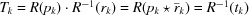, we can write our optimization measure as
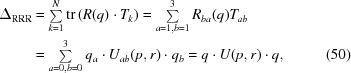
where the frame-based cross-covariance matrix is simply 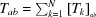 and *U*(*p*, *r*) = *U*(*T*) has the same relation to *T* as *M*(*E*) has to *E* in equation (13).

To compute the necessary 4 × 4 numerical profile matrix *U*, one need only substitute the appropriate 3D frame triads or their corresponding quaternions for the *k*th frame pair and sum over *k*. Since the orientation-frame profile matrix *U*(*p*, *r*) is symmetric and traceless just like the Euclidean profile matrix *M*, the same solution methods for the optimal quaternion rotation 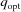 will work without alteration in this case, which is probably the preferable method for the general problem.

### 7.6. Evaluation   

The details of evaluating the properties of our quaternion-frame alignment algorithms, including comparison of the chord approximation to the arc-length measure, are available in the supporting information. The top-level result is that, even for quite large rotational differences, the mean difference between the optimal quaternion using the numerical arc-length measure and the optimal quaternion using the chord approximation for any of the three methods is on the order of small fractions of a degree for the random data distributions that we examined.

## 8. The 3D combined point + frame alignment problem   

Since we now have precise alignment procedures for both 3D spatial coordinates and 3D frame triad data (using the exact measure for the former and the approximate chord measure for the latter), we can consider the full 6 degree-of-freedom (6DOF) alignment problem for combined data from a single structure. As always, this problem can be solved either by numerical eigenvalue methods or in closed algebraic form using the eigensystem formulation of both alignment problems presented in the previous sections. While there are clearly appropriate domains of this type, *e.g.* any protein structure in the Protein Data Bank can be converted to a list of residue centers and their local frame triads (Hanson & Thakur, 2012 ▸), little is known at this time about the potential value of combined alignment. To establish the most complete possible picture, we now proceed to describe the details of our solution to the alignment problem for combined translational and rotational data, but we remark at the outset that the results of the combined system are not obviously very illuminating.

The most straightforward approach to the combined 6DOF measure is to equalize the scales of our spatial *M*(*E*) profile matrix and our orientation-frame *U*(*S*) profile matrix by imposing a unit-eigenvalue normalization, and then simply to perform a linear interpolation modified by a dimensional constant σ to adjust the relative importance of the orientation-frame portion:
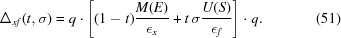
Because of the dimensional incompatibility of Δ_*x*_ and Δ_*f*_, we treat the ratio
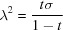
as a dimensional weight such as that adopted by Fogolari *et al.* (2016 ▸) in their entropy calculations, or implicit in the weights α and β employed in the error function of Walker *et al.* (1991 ▸). If we take *t* to be dimensionless, then σ carries the dimensional scale information.

Given the composite profile matrix of equation (51), we can now extract our optimal rotation solution by computing the maximal eigenvalue as usual, either numerically or algebraically (though we may need the extension to the non-vanishing trace case examined in the supporting information for some choices of *U*). The result is a parameterized eigensystem
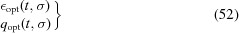
yielding the optimal values 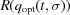, 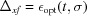 based on the data {*E*, *S*} no matter what we take as the values of the two variables (*t*, σ).

### 8.1. A simplified composite measure   

However, upon inspection of equation (51), one wonders what happens if we simply use the slerp defined in equation (8) to interpolate between the *separate* spatial and orientation-frame optimal quaternions. While the eigenvalues that correspond to the two scaled terms *M*/∊_*x*_ and *U*/∊_*f*_ in equation (51) are both unity, and thus differ from the eigenvalues of *M* and *U*, the individual normalized eigenvectors 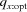 and 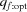 are the same. Thus, if we are happy with simply using a hand-tuned fraction of the combination of the two corresponding rotations, we can just choose a composite rotation *R*(*q*(*t*)) specified by
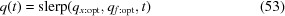
to study the composite 6DOF alignment problem. In fact, as detailed in the supporting information, if we simply plug this *q*(*t*) into equation (51) for any *t* (and σ = 1), we find *negligible differences* between the quaternions *q*(*t*) and 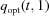 as a function of *t*. We suggest in addition that any particular effect of σ ≠ 1 could be achieved at some value of *t* in the interpolation. We thus conclude that, for all practical purposes, we might as well use equation (53) with the parameter *t* adjusted to achieve the objective of equation (51) to study composite translational and rotational alignment similarities.

## 9. Conclusion   

Our objective has been to explore quaternion-based treatments of the RMSD data-comparison problem as developed in the work of Davenport (1968 ▸), Faugeras & Hebert (1983 ▸), Horn (1987 ▸), Diamond (1988 ▸), Kearsley (1989 ▸), and Kneller (1991 ▸), among others, and to publicize the exact algebraic solutions, as well as extending the method to handle wider problems. We studied the intrinsic properties of the RMSD problem for comparing spatial-coordinate and orientation-frame data in quaternion-accessible domains, and we examined the nature of the solutions for the eigensystems of the 3D spatial-coordinate RMSD problem, as well as the corresponding 3D quaternion orientation-frame alignment problem (QFA). Extensions of both the spatial-coordinate and orientation-frame alignment problems and their solutions to 4D are detailed in the supporting information. We also examined solutions for the combined 3D spatial-coordinate and orientation-frame RMSD problem, arguing that a simple quaternion interpolation between the two individual solutions may well be sufficient for most purposes.

## Supplementary Material

Supporting information. DOI: 10.1107/S2053273320002648/ib5072sup1.pdf

## Figures and Tables

**Figure 10:**
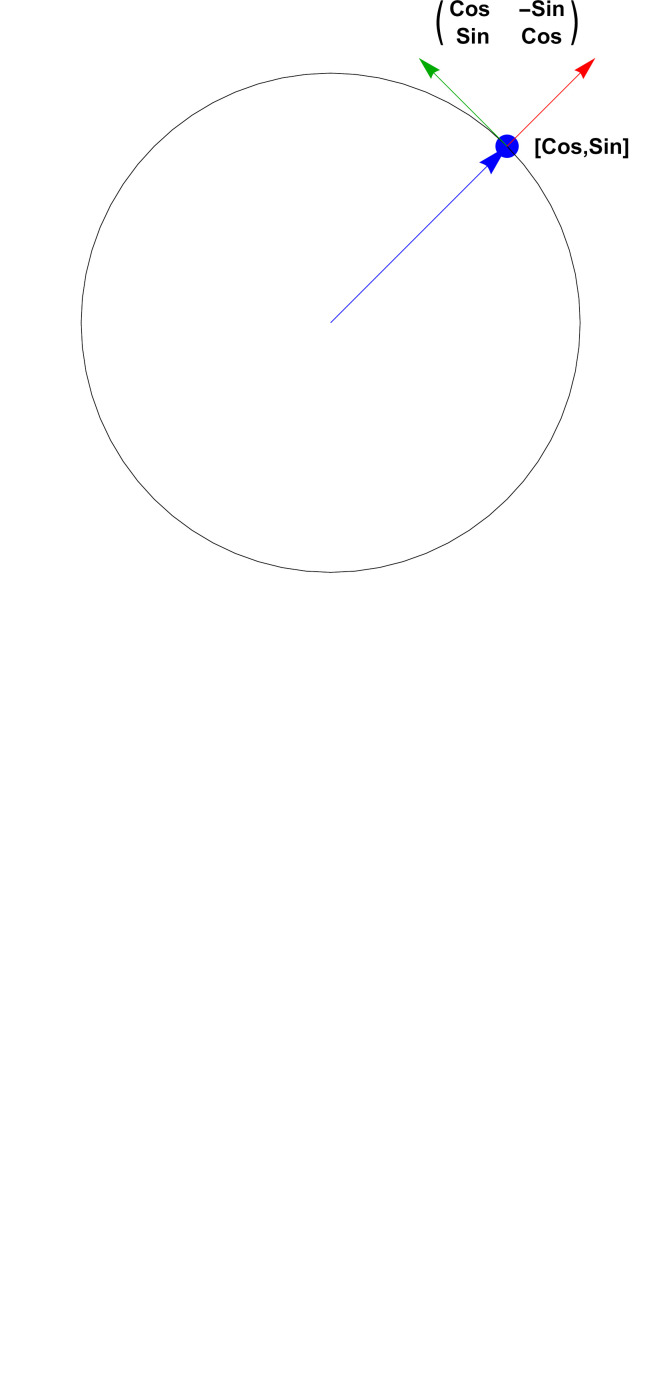
(*a*) Any standard 2D coordinate frame corresponds to the columns of an ordinary rotation matrix, and is associated to the point 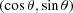 on a unit circle. (*b*) The standard 2D coordinate frames associated with a sampling of the entire circle of points 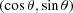.

**Figure 11:**
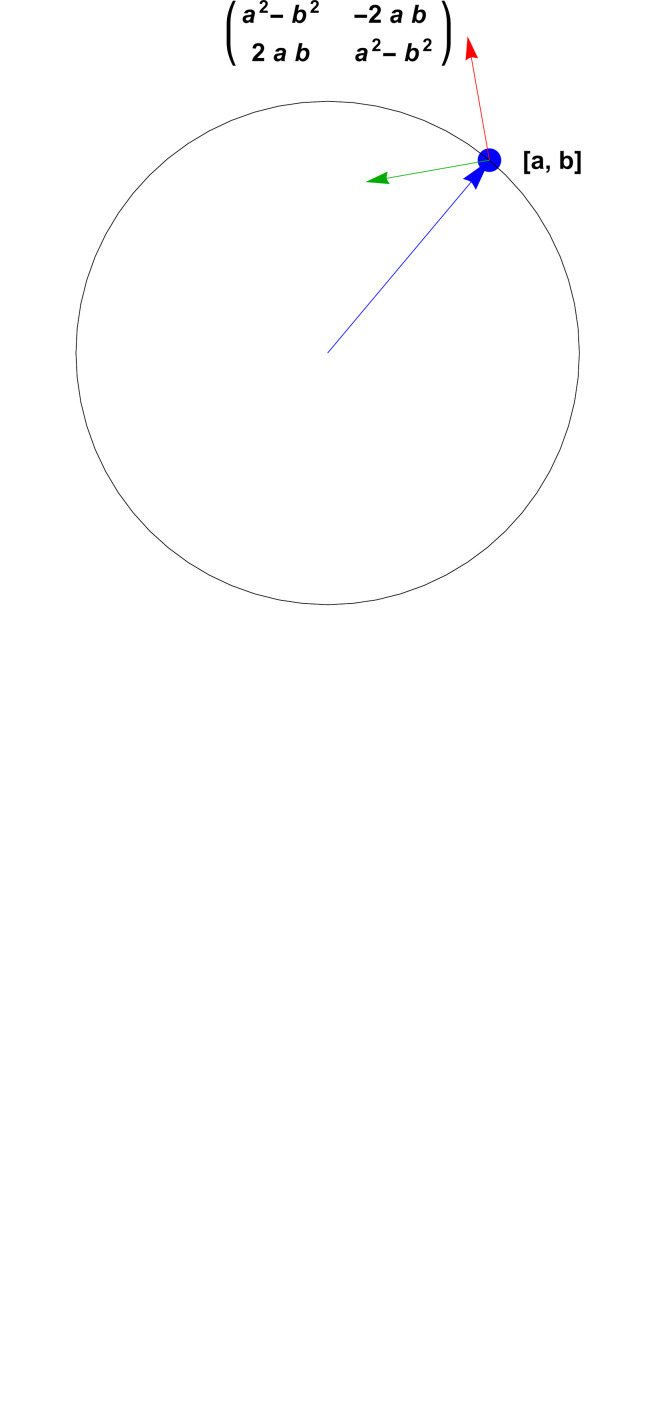
(*a*) The quaternion point (*a*, *b*), in contrast, corresponds via the double-angle formula to coordinate frames that rotate twice as rapidly as (*a*, *b*) progresses around the unit circle that is a simplified version of quaternion space. (*b*) The set of 2D frames associated with the entire circle of quaternion points (*a*, *b*); each diametrically opposite point corresponds to an identical frame. For later use in displaying full quaternions, we show how color coding can be used to encode the sign of one of the coordinates on the circle.

## Appendix A The 3D Euclidean-space least-squares matching function

This appendix works out the details of the long-form least-squares distance measure for the 3D Euclidean alignment problem using the method of Hebert and Faugeras (Hebert, 1983 ▸; Faugeras & Hebert, 1983 ▸, 1986 ▸). Starting with the 3D Euclidean minimizing distance measure equation (9), we can exploit equation (5) for *R*(*q*), along with equation (2), to produce an alternative quaternion eigenvalue problem whose *minimal* eigenvalue determines the eigenvector  specifying the matrix that rotates the test data into closest correspondence with the reference data.

Adopting the convenient notation **x** = (0, *x*_1_, *x*_2_, *x*_3_) for a pure imaginary quaternion, we employ the following steps:

Here we may write, for each *k*, the matrix *A*(**x**_*k*_, **y**_*k*_) as

where, with ‘*a*’ for ‘antisymmetric’ and ‘*s*’ for ‘symmetric,’

and, again for each *k*,

and . As using the full squared-difference minimization measure equation (9) requires the global minimal value, the solution for the optimal quaternion in equation (54) is the eigenvector of the *minimal* eigenvalue of *B* in equation (56). This is the approach used by Faugeras and Hebert in the earliest application of the quaternion method to scene alignment that we know of. While it is important to be aware of this alternative method, in the main text we have found it more useful to focus on the alternate form exploiting only the non-constant cross-term appearing in equation (9), as does most of the recent molecular structure literature. The cross-term requires the determination of the *maximal* eigenvalue rather than the *minimal* eigenvalue of the corresponding data matrix. Direct numerical calculation verifies that, though the minimal eigenvalue of equation (56) differs from the maximal eigenvalue of the cross-term approach, the exact same optimal eigenvector is obtained, a result that can presumably be proven algebraically but that we will not need to pursue here.

## Appendix B Introduction to quaternion orientation frames

### b1. What is a quaternion frame?

We will first present a bit of intuition about coordinate frames that may help some readers with our terminology. If we take the special case of a quaternion representing a rotation in the 2D (*x*, *y*) plane, the 3D rotation matrix equation (5) reduces to the standard right-handed 2D rotation

As shown in Fig. 10 ▸(*a*), we can use θ to define a unit direction in the complex plane defined by , and then the *columns* of the matrix *R*_2_(θ) naturally correspond to a unique associated 2D coordinate frame diad; an entire collection of points *z* and their corresponding frame diads are depicted in Fig. 10 ▸(*b*).

Starting from this context, we can get a clear intuitive picture of what we mean by a ‘quaternion frame’ before diving into the quaternion RMSD problem. The essential step is to look again at equation (5) for *n*_*x*_ = 1, and write the corresponding quaternion as (*a*, *b*, 0, 0) with *a* ^2^ + *b* ^2^ = 1, so this is a ‘2D quaternion’, and is indistinguishable from a complex phase like  that we just introduced. There is one significant difference, however, and that is that equation (5) shows us that *R*_2_(θ) takes a new form, quadratic in *a* and *b*,

Using either the formula (7) for  or just exploiting the trigonometric double-angle formulas, we see that equation (57) and equation (58) correspond and that

Our simplified 2D quaternion thus describes the *square root* of the usual Euclidean frame given by the columns of *R*_2_(θ). Thus the pair (*a*, *b*) (the reduced quaternion) itself corresponds to a frame. In Fig. 11 ▸(*a*), we show how a given ‘quaternion frame’, *i.e.* the columns of *R*_2_(*a*, *b*), corresponds to a point *u* = *a* + i*b* in the complex plane. Diametrically *opposite* points (*a*, *b*) and (−*a*, − *b*) now correspond to the *same* frame! Fig. 11 ▸(*b*) shows the corresponding frames for a large collection of points (*a*, *b*) in the complex plane, and we see the new and unfamiliar feature that the frames make *two* full rotations on the complex circle instead of just one as in Fig. 10 ▸(*b*).

This is what we have to keep in mind as we now pass to using a full quaternion to represent an arbitrary 3D frame triad via equation (5). The last step is to notice that in Fig. 11 ▸(*b*) we can represent the set of frames in one half of the complex circle, *a* ≥ 0 shown in magenta, as distinct from those in the other half, *a* < 0 shown in dark blue; for any value of *b*, the vertical axis, there is a *pair* of *a*’s with opposite signs and colors. In the quaternion case, we can display quaternion frames inside one single sphere, like displaying only the *b* coordinates in Fig. 11 ▸(*b*) projected to the vertical axis, realizing that if we know the sign-correlated coloring, we can determine both the magnitude of the dependent variable *a* = ±(1 − *b* ^2^)^1/2^ as well as its sign. The same holds true in the general case: if we display only a quaternion’s 3-vector part **q** = (*q*_*x*_, *q*_*y*_, *q*_*z*_) along with a color specifying the sign of *q*_0_, we implicitly know both the magnitude and sign of , and such a 3D plot therefore accurately depicts *any* quaternion. Another alternative employed in the main text is to use *two* solid balls, one a ‘northern hemisphere’ for the *q*_0_ ≥ 0 components and the other a ‘southern hemisphere’ for the *q*_0_ ≤ 0 components. Each may be useful in different contexts.

### b2. Example

We illustrate all this in Fig. 12 ▸(*a*), which shows a typical collection of quaternion reference-frame data displaying only the **q** components of (*q*_0_, **q**); the *q*_0_ ≥ 0 data are mixed with the *q*_0_ < 0 data, but are distinguished by their color coding. In Fig. 12 ▸(*b*), we show the frame triads resulting from applying equation (5) to each quaternion point and plotting the result at the associated point **q** in the display.

## Appendix C On obtaining quaternions from rotation matrices

The quaternion RMSD profile matrix method can be used to implement a singularity-free algorithm to obtain the (sign-ambiguous) quaternions corresponding to numerical 3D and 4D rotation matrices. There are many existing approaches to the 3D problem in the literature [see, *e.g.*, Shepperd (1978 ▸), Shuster & Natanson (1993 ▸), or Section 16.1 of Hanson (2006 ▸)]. In contrast to these approaches, Bar-Itzhack (2000 ▸) has observed, in essence, that if we simply replace the data matrix *E*_*ab*_ by a numerical 3D orthogonal rotation matrix *R*, the numeric quaternion *q* that corresponds to , as defined by equation (5), can be found by solving our familiar maximal quaternion eigenvalue problem. The initially unknown optimal matrix (technically its quaternion) computed by maximizing the similarity measure is equivalent to a single-element quaternion barycenter problem, and the construction is designed to yield a best approximation to *R* itself in quaternion form. To see this, take *S*(*r*) to be the sought-for optimal rotation matrix, with its own quaternion *r*, that must maximize the Bar-Itzhack measure. We start with the Fröbenius measure describing the match of two rotation matrices corresponding to the quaternion *r* for the unknown quaternion and the numeric matrix *R* containing the known 3 × 3 rotation matrix data:

Pulling out the cross-term as usual and converting to a maximization problem over the unknown quaternion *r*, we arrive at

where *R* is (approximately) an orthogonal matrix of numerical data and *K*(*R*) is analogous to the profile matrix *M*(*E*). Now *S* is an abstract rotation matrix and *R* is supposed to be a good numerical approximation to a rotation matrix, and thus the product  should also be a good approximation to an **SO**(3) rotation matrix; hence that product itself corresponds closely to some axis  and angle θ, where (supposing we knew *R*’s exact quaternion *q*)

The maximum is obviously close to *T* being the identity matrix, with the ideal value at θ = 0, corresponding to *S* ≈ *R*. Thus if we find the maximal quaternion eigenvalue  of the profile matrix *K*(*R*) in equation (61), our closest solution is well represented by the corresponding normalized eigenvector ,

This numerical solution for *q* will correspond to the targeted numerical rotation matrix, solving the problem. To complete the details of the computation, we replace the elements *E*_*ab*_ in equation (13) by a general orthonormal rotation matrix with columns **X** = (*x*_1_, *x*_2_, *x*_3_), **Y** = (*y*_1_, *y*_2_, *y*_3_) and **Z** = (*z*_1_, *z*_2_, *z*_3_), scaling by 1/3, thus obtaining the special 4 × 4 profile matrix *K* whose elements in terms of a known numerical matrix *R* = [**X**|**Y**|**Z**] (transposed in the algebraic expression for *K* owing to the ) are

Determining the algebraic eigensystem of equation (63) is a non-trivial task. However, as we know, any orthogonal 3D rotation matrix *R*(*q*), or equivalently, , can also be ideally expressed in terms of quaternions via equation (5), and this yields an alternate useful algebraic form:

This equation then allows us to quickly prove that *K* has the correct properties to solve for the appropriate quaternion corresponding to *R*. First we note that the coefficients *p*_*n*_ of the eigensystem are simply constants,

Computing the eigenvalues and eigenvectors using the symbolic quaternion form, we see that the eigenvalues are constant, with maximal eigenvalue exactly one, and the eigenvectors are almost trivial, with the maximal eigenvector being the quaternion *q* that corresponds to the (numerical) rotation matrix:

The first column is the quaternion , with . (This would be 3 if we had not divided by 3 in the definition of *K*.)

*Alternate version.* From the quaternion-barycenter work of Markley *et al.* (2007 ▸) and the natural form of the quaternion-extraction problem in 4D in the supporting information, we know that equation (64) actually has a much simpler form with the same unit eigenvalue and natural quaternion eigenvector. If we simply take equation (64) multiplied by 3, add the constant term , and divide by 4, we get a more compact quaternion form of the matrix, namely

This has vanishing determinant and trace , with all other *p*_*k*_ coefficients vanishing, leading to an eigensystem identical to equation (64):

As elegant as this is, in practice our numerical input data are from the 3 × 3 matrix *R* itself, and not the quaternions, so we will almost always just use those numbers in equation (63) to solve the problem.

### c1. Completing the solution

In typical applications, *the solution is immediate, requiring only trivial algebra*. The maximal eigenvalue is always known in advance to be unity for any valid rotation matrix, so we need only to compute the eigenvector from the numerical matrix equation (63) with unit eigenvalue. We simply compute any column of the adjugate matrix of [*K*(*R*) − *I*_4_], or solve the equivalent linear equations of the form

As always, one may need to check for degenerate special cases.

### c2. Non-ideal cases

It is important to note, as emphasized by Bar-Itzhack, that if there are *significant errors* in the numerical matrix *R*, then the actual non-unit maximal eigenvalue of *K*(*R*) can be computed numerically or algebraically as usual, and then that eigenvalue’s eigenvector determines the *closest* normalized quaternion to the errorful rotation matrix, which can be very useful since such a quaternion always produces a valid rotation matrix.

In any case, *up to an overall sign*,  is the desired numerical quaternion *q* corresponding to the target numerical rotation matrix *R* = *R*(*q*). In some circumstances one is looking for a uniform statistical distribution of quaternions, in which case the overall sign of *q* should be chosen *randomly*.

The Bar-Itzhack approach solves the problem of extracting the quaternion of an arbitrary numerical 3D rotation matrix in a fashion that involves no singularities and only trivial testing for special cases, thus essentially making the traditional methods obsolete. The extension of Bar-Itzhack’s method to the case of 4D rotations is provided in the supporting information.

## Appendix D On defining the quaternion barycenter

The notion of a *Riemannian barycenter* is generally associated with the work of Grove *et al.* (1974 ▸), and may also be referred to as the *Karcher mean* (Karcher, 1977 ▸), defined as the point that minimizes the sum of squared geodesic distances from the elements of a collection of fixed points on a manifold. The general class of such optimization problems has also been studied, *e.g.*, by Manton (2004 ▸). We are interested here in the case of quaternions, which we know are points on the spherical 3-manifold **S** ^3^ defined by the unit-quaternion subspace of  restricted to *q* · *q* = 1 for any point *q* in . This subject has been investigated by a number of authors, with Brown & Worsey (1992 ▸) discussing the problems with this computation in 1992, and Buss & Fillmore (2001 ▸) proposing a solution applicable to computer-graphics 3D orientation interpolation problems in 2001, inspired to some extent by Shoemake’s 1985 introduction of the quaternion slerp as a way to perform geodesic orientation interpolations in 3D using equation (5) for *R*(*q*). There are a variety of methods and studies related to the quaternion-barycenter problem. In 2002, Moahker published a rigorous account on averaging in the group of rotations (Moakher, 2002 ▸), while subsequent treatments included the work by Markley *et al.* (2007 ▸), focusing on aerospace and astronomy applications, and the comprehensive review by Hartley *et al.* (2013 ▸), aimed in particular at the machine vision and robotics community, with additional attention to conjugate rotation averaging (the ‘hand–eye calibration’ problem in robotics) and multiple rotation averaging. While we have focused on measures starting from sums of squares that lead to closed-form optimization problems, Hartley *et al.* (2011 ▸) have carefully studied the utility of the corresponding *L*_1_ norm and the iterative Weiszfeld algorithm for finding its optimal solution numerically.

The task at hand is basically to extend the Bar-Itzhack algorithm to an entire collection of frames instead of a single rotation. We need to find an optimal rotation matrix *R*(*q*) that corresponds to the quaternion point closest to the geodesic center of an *unordered* set of reference data. We already know that the case of the ‘barycenter’ of a single orientation frame is solved by the Bar-Itzhack algorithm of Appendix C[App appc], which finds the quaternion closest to a single item of rotation matrix data (the quaternion barycenter of a single rotation is itself). For two items of data, *R*_1_ = *R*(*q*_1_) and *R*_2_ = *R*(*q*_2_), the quaternion barycenter is determined by the slerp interpolator to be

For three or more items, no closed form is currently known.

We start with a data set of *N* rotation matrices *R*(*p*_*k*_) that are represented by the quaternions *p*_*k*_, and we want *R*(*q*) to be as close as possible to the set of *R*(*p*_*k*_). That rotation matrix, or its associated quaternion point, are the orientation-frame analogs of the Euclidean barycenter for a set of Euclidean points. As before, it is clear that the mathematically most justifiable measure employs the *geodesic arc length* on the quaternion sphere; but to the best of anyone’s knowledge, there is no way to apply linear algebra to find the corresponding . Achieving a numerical solution to that problem is the task solved by Buss & Fillmore (2001 ▸), as well as a number of others, including, *e.g.*, Moakher (2002 ▸), Markley *et al.* (2007 ▸), and Hartley *et al.* (2013 ▸). The problem that we can understand algebraically is, once again, the approximate *chord measure*. For the case treated in Section 7, with the assumption that we can consistently use the quaternions themselves, we could find a solution using just the Euclidean chord average *V*/*N* from , so the optimal quaternion for the measure  was simply .

However, if that is not an option, and we require an average rotation that is based more directly on the sign-insensitive rotation matrices themselves, we need to use the Δ_RRR_ method of Section 7.5. This turns out to be essentially an extension of the Bar-Itzhack algorithm from a single rotation to a collection of rotations, and that is the approach we present here. Our starting point is the sign-insensitive Fröbenius measure , giving us the following starting point:

Dropping the constants and converting as usual to maximize over the cross-term instead of minimizing the distance measure, we define a tentative spherical barycenter as the maximum of the variation over the quaternion *q* of the following:

where for each *k* = 1, …, *N* our first guess at the profile matrix is

But if, as pointed out by Markley *et al.* (2007 ▸), we simply add one copy of the identity matrix in the form  to the matrix *K*(*p*), we get a much simpler matrix that we can use instead because constants do not affect the optimization process. Our partial profile matrix for each *k* = 1, …, *N* is now

or to be precise, after the sum over *k*, the profile matrix in terms of the quaternion columns [*p*_*k*_] of **P** becomes

with quaternion indices (*a*, *b*) ranging from 0 to 3. Finally, we can write the expression for the chord-based barycentric measure to be optimized to get  as

We recognize this as equation (49) of Section 7, identified there as related to the rotation averaging problem, re-derived here as an extension of the Bar-Itzhack method. Note that we now have the added insight that since for the cases *N* = 1, 2, 3, the rank of *K*(**P**) is known to be 1, 2, 3, respectively, the chord-measure barycenter is computable with linear (Bar-Itzhack), quadratic and cubic algebraic forms. For larger *N*, the eigensystems are all quartic.

We also have another option: if, for some reason, we only have numerical 3 × 3 rotation matrices *R*_*k*_ and not their associated quaternions *p*_*k*_, we can recast equation (72) in terms of equation (63) for each matrix *R*_*k*_ and use the sum over *k* of *those* numerical matrices to extract our optimal quaternion. This is non-trivial because the simple eigensystem form of the profile matrix *K* for the Bar-Itzhack task was valid only for *one* rotation data matrix, and as soon as we start summing over additional matrices all of that simplicity disappears, though the eigensystem problem remains intact.

Optimizing the approximate chord measure for the ‘average rotation’, the ‘quaternion average’, or the spherical barycenter of the quaternion orientation-frame data set {*p*_*k*_} (or {*R*_*k*_}) now just reduces, as before, to finding the (normalized) eigenvector corresponding to the largest eigenvalue of *K*. It is also significant that the initial *K*_trial_ matrix in equation (73) is *traceless*, and so the traceless algebraic eigenvalue methods would apply, while the simpler *K* matrix in equation (75) is *not traceless*, and thus, in order to apply the algebraic eigenvalue method, we would have to use the generalization presented in the supporting information that includes an arbitrary trace term.
