# Supplementary material for: The quaternion-based spatial-coordinate and orientation-frame alignment problems
Source: Acta Crystallogr A Found Adv. 2020 Jun 18;76(Pt 4):432–57. doi: 10.1107/S2053273320002648 (PMC7330932; doi:10.1107/S2053273320002648)
Supplement: Supplementary file 1 [file a-76-00432-sup1.pdf]

FOUNDATIONS
ADVANCES

Volume 76 (2020)

Supporting information for article:

The quaternion-based spatial-coordinate and orientation-frame alignment problems

Andrew J. Hanson

Acta Crystallographica Section A
Foundations and Advances
ISSN 2053-2733

Supporting information: The quaternion-based spatial-coordinate and orientation-frame alignment problems

Andrew J. Hanson *

Luddy School of Informatics, Computing, and Engineering
Indiana University, Bloomington, Indiana United States. Correspondence e-mail: hansona@indiana.edu

Supporting information for the paper *The quaternion-based spatial-coordinate and orientation-frame alignment problems* is presented here. The most significant additional result is the extension of the 3D treatment in the main text to four dimensions. Following a review of quaternion properties now including the representation of 4D rotations using quaternion pairs, we give a detailed study of the 4D quaternion-based spatial-coordinate alignment problem, which is significantly different from the 3D problem in the main text. Next, we use the 4D quaternion rotation method to extend our treatment to 4D orientation-frame alignment. The 3D Bar-Itzhak profile-matrix method for extracting a quaternion from a 3D numerical rotation matrix is extended to 4D numerical rotation matrices, followed by a look at the algebraic solutions of 2D alignment problems, whose deceptive simplicity does not carry over to the 3D and 4D cases. Finally, we supplement the 3D orientation alignment section of the main text with careful studies of the properties, limitations, and features of our 3D orientation frame alignment methods, followed by an extended exposition and analysis of the strengths and weaknesses of the 6DOF combined spatial and orientation frame alignment techniques. The Appendix provides a comprehensive study of the quartic equation solutions to eigenvalue problems, focusing on applications to the eigensystems of real symmetric matrices.

© 2020 International Union of Crystallography

1. Foundations of Quaternions for 3D and 4D Problems

We begin with a review of quaternion properties used in the 3D analysis, folding in some additional details, and then systematically add the extensions that are exploited to handle the 4D case. The treatment here is designed to be self-contained, repeating any relevant material from the main paper, thus avoiding any confusion involving cross-references to the main paper for equations and conceptual background.

Quaternions for 3D Analysis. We take a quaternion to be a point $q = (q_0, q_1, q_2, q_3) = (q_0, \mathbf{q})$ in 4D Euclidean space with unit norm, $q \cdot q = 1$ (see, e.g., (Hanson, 2006) for further details about quaternions). The last three terms,

About the Author: *Andrew J. Hanson is an Emeritus Professor of Computer Science at Indiana University. He earned a bachelor's degree in Chemistry and Physics from Harvard University in 1966 and a PhD in Theoretical Physics from MIT in 1971. His interests range from general relativity to computer graphics, artificial intelligence, and bioinformatics; he is particularly concerned with applications of quaternions and with exploitation of higher-dimensional graphics for the visualization of complex scientific contexts such as Calabi-Yau spaces. He is the co-discoverer of the Eguchi-Hanson "gravitational instanton" Einstein metric (1978), author of* Visualizing Quaternions *(Elsevier, 2006), and designer of the iPhone Apps* 4Dice *and* 4DRoom *(2012) for interacting with four-dimensional virtual reality.*

$\mathbf{q}$, play the role of a generalized imaginary number, so the conjugation operation is $\bar{q} = (q_0, -\mathbf{q})$. Quaternions obey a multiplication operation denoted by $\star$ and defined as follows:

$$q \star p = Q(q) \cdot p = \begin{bmatrix} q_0 & -q_1 & -q_2 & -q_3 \\ q_1 & q_0 & -q_3 & q_2 \\ q_2 & q_3 & q_0 & -q_1 \\ q_3 & -q_2 & q_1 & q_0 \end{bmatrix} \cdot \begin{bmatrix} p_0 \\ p_1 \\ p_2 \\ p_3 \end{bmatrix} = (q_0 p_0 - \mathbf{q} \cdot \mathbf{p},\ q_0 \mathbf{p} + p_0 \mathbf{q} + \mathbf{q} \times \mathbf{p}) \ , \tag{1}$$

where the orthonormal matrix $Q(q)$ is an alternative form of quaternion multiplication that explicitly demonstrates that the action of q on p by quaternion multiplication *literally* rotates the quaternion unit vector p in 4D Euclidean space. Another non-trivial matrix form of quaternion multiplication that is useful in some calculations is the left-acting matrix $\tilde{Q}$ producing a *right* multiplication,

$$q \star p = \tilde{Q}(p) \cdot q = \begin{bmatrix} p_0 & -p_1 & -p_2 & -p_3 \\ p_1 & p_0 & p_3 & -p_2 \\ p_2 & -p_3 & p_0 & p_1 \\ p_3 & p_2 & -p_1 & p_0 \end{bmatrix} \cdot \begin{bmatrix} q_0 \\ q_1 \\ q_2 \\ q_3 \end{bmatrix} . \tag{2}$$

Choosing exactly one of the three imaginary components in both q and p to be nonzero gives back the classic complex algebra $(q_0 + \mathrm{i} q_1)(p_0 + \mathrm{i} p_1) = (q_0 p_0 - q_1 p_1) + \mathrm{i}\,(q_0 p_1 + p_0 q_1)$, so there are three copies of the complex numbers embedded in the quaternion algebra; the difference is that in general the final term $\mathbf{q} \times \mathbf{p}$ changes sign if one reverses the order, making the quaternion product order-dependent, unlike the complex product. Quaternions also satisfy the nontrivial "multiplicative norm" relation

$$\|q\|\,\|p\| = \|q \star p\| \ , \tag{3}$$

where $\|q\|^2 = q \cdot q = \Re(q \star \bar{q})$, that uniquely characterizes the real, complex, quaternion, and octonion number systems comprising the Hurwitz algebras. Quaternions also obey a number of interesting scalar triple-product identities,

$$r \cdot (q \star p) = q \cdot (r \star \bar{p}) = \bar{p} \cdot (\bar{r} \star q) = \bar{r} \cdot (\bar{p} \star \bar{q}) \ , \tag{4}$$

where the complex conjugate entries are the natural consequences of the sign changes occurring only in the (imaginary) 3D part.

Conjugating a vector $\mathbf{x} = (x, y, z)$ written as a purely "imaginary" quaternion $(0, \mathbf{x})$ by quaternion multiplication is isomorphic to the construction of a 3D Euclidean rotation $R(q)$ generating all possible elements of the special orthogonal group $\mathbf{SO}(3)$. If we compute

$$q \star (c,\ x,\ y,\ z) \star \bar{q} = (c,\ R_3(q) \cdot \mathbf{x}) \ , \tag{5}$$

we see that only the purely imaginary part is affected, whether or not the arbitrary real constant $c = 0$. Collecting coefficients gives this fundamental form of an arbitrary 3D rotation expressed in terms of quaternions,

$$\left.\begin{aligned} [R_3(q)]_{ij} &= \delta_{ij}\left(q_0{}^2 - \mathbf{q}^2\right) + 2 q_i q_j - 2\epsilon_{ijk} q_0 q_k \\ R_3(q) &= \begin{bmatrix} q_0{}^2 + q_1{}^2 - q_2{}^2 - q_3{}^2 & 2q_1q_2 - 2q_0q_3 & 2q_1q_3 + 2q_0q_2 \\ 2q_1q_2 + 2q_0q_3 & q_0{}^2 - q_1{}^2 + q_2{}^2 - q_3{}^2 & 2q_2q_3 - 2q_0q_1 \\ 2q_1q_3 - 2q_0q_2 & 2q_2q_3 + 2q_0q_1 & q_0{}^2 - q_1{}^2 - q_2{}^2 + q_3{}^2 \end{bmatrix} \end{aligned}\right\} , \tag{6}$$

where the mapping from q to $R_3(q)$ is two-to-one because $R_3(q) = R_3(-q)$. Note that $R_3(q)$ is a *proper* rotation, with determinant $\det R_3(q) = (q \cdot q)^3 = +1$, and that the identity quaternion $q_{\mathrm{ID}} = (1, 0, 0, 0) \equiv q \star \bar{q}$ corresponds to the identity rotation matrix, as does $-q_{\mathrm{ID}} = (-1, 0, 0, 0)$. The *columns* of $R_3(q)$ are exactly the needed quaternion

representation of the *frame triad* describing the orientation of a body in 3D space, i.e., the columns are the vectors of the frame's local x, y, and z axes relative to an initial identity frame. Choosing the following parameterization preserving $q \cdot q = 1$ (with $\hat{\mathbf{n}} \cdot \hat{\mathbf{n}} = 1$),

$$q = (\cos(\theta/2),\ \hat{n}_1 \sin(\theta/2),\ \hat{n}_2 \sin(\theta/2),\ \hat{n}_3 \sin(\theta/2)) \ , \tag{7}$$

gives the "axis-angle" form of the rotation matrix,

$$R_3(q) = R_3(\theta, \hat{\mathbf{n}}) = \begin{bmatrix} \cos\theta + (1-\cos\theta)\,\hat{n}_1^2 & (1-\cos\theta)\,\hat{n}_1\hat{n}_2 - \sin\theta\,\hat{n}_3 & (1-\cos\theta)\,\hat{n}_1\hat{n}_3 + \sin\theta\,\hat{n}_2 \\ (1-\cos\theta)\,\hat{n}_1\hat{n}_2 + \sin\theta\,\hat{n}_3 & \cos\theta + (1-\cos\theta)\,\hat{n}_2^2 & (1-\cos\theta)\,\hat{n}_2\hat{n}_3 - \sin\theta\,\hat{n}_1 \\ (1-\cos\theta)\,\hat{n}_1\hat{n}_3 - \sin\theta\,\hat{n}_2 & (1-\cos\theta)\,\hat{n}_2\hat{n}_3 + \sin\theta\,\hat{n}_1 & \cos\theta + (1-\cos\theta)\,\hat{n}_3^2 \end{bmatrix} . \tag{8}$$

This form of the 3D rotation exposes the fact that the direction $\hat{\mathbf{n}}$ is fixed, so $\hat{\mathbf{n}}$ is the lone real eigenvector of R_3. Multiplying a quaternion p by the quaternion q to get a new quaternion $p' = q \star p$ simply *rotates* the 3D frame corresponding to p by the matrix Eq. (6) written in terms of q, so

$$R_3(q \star p) = R_3(q) \cdot R_3(p) \ , \tag{9}$$

and this collapse of repeated rotation matrices into a single rotation matrix with a quaternion-product argument can be continued indefinitely.

Remark: *Eigensystem and properties of* R_3*:* One of our themes is constructing and understanding eigensystems of interesting matrices, so here, as an aside, we expand the content of the previous paragraph to include some additional details. First, note that we have two ways of writing the 3D rotation, as $R_3(\theta, \hat{\mathbf{n}})$ and as $R_3(q)$. Thus there are two ways to write the eigenvalues, which we can compute to be

$$\left\{ \begin{array}{c} 1 \\ e^{\mathrm{i}\theta} \\ e^{-\mathrm{i}\theta} \end{array} \right\} \qquad \left\{ \begin{array}{c} 1 \\ (q_0{}^2 - q_1{}^2 - q_2{}^2 - q_3{}^2 + 2\mathrm{i}q_0\sqrt{q_1{}^2 + q_2{}^2 + q_3{}^2}) \\ (q_0{}^2 - q_1{}^2 - q_2{}^2 - q_3{}^2 - 2\mathrm{i}q_0\sqrt{q_1{}^2 + q_2{}^2 + q_3{}^2}) \end{array} \right\} , \tag{10}$$

respectively, where the two columns are of course identical, but we have chosen expressions in q (along with an implicit choice of square root sign determining $\sin(\theta/2)$) that match exactly with the $R_3(q)$ eigenvectors. Those eigenvectors (unnormalized for notational clarity) can be written as:

$$\left\{ \begin{bmatrix} n_1 \\ n_2 \\ n_3 \end{bmatrix} \begin{bmatrix} -\mathrm{i}\,n_2 - n_1 n_3 \\ \mathrm{i}\,n_1 - n_2 n_3 \\ n_1{}^2 + n_2{}^2 \end{bmatrix} \begin{bmatrix} +\mathrm{i}\,n_2 - n_1 n_3 \\ -\mathrm{i}\,n_1 - n_2 n_3 \\ n_1{}^2 + n_2{}^2 \end{bmatrix} \right\}$$

$$\left\{ \begin{bmatrix} q_1 \\ q_2 \\ q_3 \end{bmatrix} \begin{bmatrix} -\,q_1 q_3 - \mathrm{i}\,q_2\sqrt{q_1{}^2 + q_2{}^2 + q_3{}^2} \\ -\,q_2 q_3 + \mathrm{i}\,q_1\sqrt{q_1{}^2 + q_2{}^2 + q_3{}^2} \\ q_1{}^2 + q_2{}^2 \end{bmatrix} \begin{bmatrix} -\,q_1 q_3 + \mathrm{i}\,q_2\sqrt{q_1{}^2 + q_2{}^2 + q_3{}^2} \\ -\,q_2 q_3 - \mathrm{i}\,q_1\sqrt{q_1{}^2 + q_2{}^2 + q_3{}^2} \\ q_1{}^2 + q_2{}^2 \end{bmatrix} \right\} \tag{11}$$

where we emphasize that in general R_3 has only one real eigenvalue (which is unity), whose eigenvector is the direction of the 3D axis $\hat{\mathbf{n}}$ invariant under that particular rotation. Since any quaternion can be written in the form Eq. (7), the trace of any rotation can be written as

$$\operatorname{tr} R_3 = 3q_0{}^2 - q_1{}^2 - q_2{}^2 - q_3{}^2 \ = \ 4q_0{}^2 - 1 \ = \ 1 + 2\cos\theta \ , \tag{12}$$

which follows from the half-angle formula. This means that, in the RMSD formula maximizing $\operatorname{tr}(R_3 \cdot E)$, if E is an identity matrix, the rotation giving the maximal trace corresponds to R_3 being the identity matrix, $\theta = 0$, and if E is a

rotation matrix, the maximal trace occurs when the product of the two matrices has vanishing angle θ for the *composite* matrix produced by the product of their two quaternions, so the optimal rotation matrix R_3 is the inverse of E. This property is exploited in the Bar-Itzhack algorithm given in Section 4.

The Slerp. Relationships among quaternions can be studied using the *slerp*, or "spherical linear interpolation" (Shoemake, 1985; Jupp & Kent, 1987), that smoothly parameterizes the points on the shortest geodesic quaternion path between two constant (unit) quaternions, q_0 and q_1, as

$$slerp(q_0, q_1, s) \equiv q(s)[q_0, q_1] \quad = \quad q_0 \frac{\sin((1-s)\phi)}{\sin\phi} + q_1 \frac{\sin(s\,\phi)}{\sin\phi} \; . \qquad (13)$$

Here $\cos\phi = q_0 \cdot q_1$ defines the angle ϕ between the two given quaternions, while $q(s=0) = q_0$ and $q(s=1) = q_1$. The "long" geodesic can be obtained for $1 \leq s \leq 2\pi/\phi$. For small ϕ, this reduces to the standard linear interpolation $(1-s)\,q_0 + s\,q_1$. The unit norm is preserved, $q(s) \cdot q(s) = 1$ for all s, so $q(s)$ is always a valid quaternion and $R(q(s))$ defined by Eq. (6) is always a valid 3D rotation matrix. We note that one can formally write Eq. (13) as an exponential of the form $q_0 \star (\bar{q}_0 \star q_1)^s$, but since this requires computing a logarithm and an exponential whose most efficient reduction to a practical computer program is Eq. (13), this is mostly of pedagogical interest.

Double Quaternions and 4D Rotations. We now extend Eq. (6) from three Euclidean dimensions to four Euclidean dimensions by choosing two *distinct* quaternions and generalizing Eq. (5) to 4D points $\mathbf{x}_4 = (w, x, y, z)$ as follows:

$$p \star (w,\ x,\ y,\ z) \star \bar{q} = R_4(p, q) \cdot \mathbf{x}_4 \; . \qquad (14)$$

Here R_4 turns out to be an orthonormal 4D rotation matrix that is quadratic in the *pair* (p, q) of unit quaternion elements, which together have exactly the six degrees of freedom required for the most general 4D Euclidean rotation in the special orthogonal group $\mathbf{SO}(4)$. The algebraic form of this 4D rotation matrix is

$$R_4(p,q) = \left[\begin{array}{cccc} p_0q_0 + p_1q_1 + p_2q_2 + p_3q_3 & -p_1q_0 + p_0q_1 + p_3q_2 - p_2q_3 & -p_2q_0 - p_3q_1 + p_0q_2 + p_1q_3 & -p_3q_0 + p_2q_1 - p_1q_2 + p_0q_3 \\ p_1q_0 - p_0q_1 + p_3q_2 - p_2q_3 & p_0q_0 + p_1q_1 - p_2q_2 - p_3q_3 & -p_3q_0 + p_2q_1 + p_1q_2 - p_0q_3 & p_2q_0 + p_3q_1 + p_0q_2 + p_1q_3 \\ p_2q_0 - p_3q_1 - p_0q_2 + p_1q_3 & p_3q_0 + p_2q_1 + p_1q_2 + p_0q_3 & p_0q_0 - p_1q_1 + p_2q_2 - p_3q_3 & -p_1q_0 - p_0q_1 + p_3q_2 + p_2q_3 \\ p_3q_0 + p_2q_1 - p_1q_2 - p_0q_3 & -p_2q_0 + p_3q_1 - p_0q_2 + p_1q_3 & p_1q_0 + p_0q_1 + p_3q_2 + p_2q_3 & p_0q_0 - p_1q_1 - p_2q_2 + p_3q_3 \end{array}\right] , \qquad (15)$$

where $\det R_4(p,q) = (p \cdot p)^2 (q \cdot q)^2 = +1$ and $\operatorname{tr} R_4(p,q) = 4p_0q_0$. Since this is a quadratic form in p and q, the rotation is unchanged under $(p,q) \to (-p,-q)$, and the quaternions are again a double covering. If we set $p = q$, we recover a matrix that leaves the w component invariant, and is just the rotation Eq. (6) for the $\mathbf{x}_3 = (x, y, z)$ component. We also find the interesting result that $R_4(q, q_{\mathrm{ID}}) = Q(q)$ from Eq. (1), and $R_4(q_{\mathrm{ID}}, \bar{p}) = \widetilde{Q}\,(p)$ from Eq. (2).

Rotations in 4D can be composed in quaternion form parallel to the 3D case, with

$$R_4(p,q) \cdot R_4(p', q') = R_4(p \star p',\ q \star q') \; .$$

We observe that the 4D columns of Eq. (15) can be used to define 4D Euclidean orientation frames in the same fashion as the 3D columns of Eq. (6), and we will exploit this to treat the 4D orientation-frame alignment problem below.

Remark: *Eigensystem and properties of* R_4*:* We can also compute the eigenvalues of our 4D rotation matrix $R_4(p,q)$ from Eq. (15). The 3D form of $R_3(q)$ in terms of explicit fixed axes that we used does not have an exact analog in 4D because 4D rotations leave a *plane* invariant, not an axis. Nevertheless, we can still find very compact form for the 4D

eigenvalues. Our exact 4D analog of Eq. (10), after applying the transformations ${q_1}^2 + {q_2}^2 + {q_3}^2 \to 1 - {q_0}^2$ for q and p to simplify the expression, is just

$$\left\{ \begin{array}{l} p_0 q_0 - \sigma(p_0,q_0)\left(+\sqrt{(1-{p_0}^2)\,(1-{q_0}^2)} + \mathrm{i}\sqrt{(1-{p_0}^2)\,{q_0}^2} + \mathrm{i}\sqrt{{p_0}^2\,(1-{q_0}^2)}\right) \\ p_0 q_0 - \sigma(p_0,q_0)\left(+\sqrt{(1-{p_0}^2)\,(1-{q_0}^2)} - \mathrm{i}\sqrt{(1-{p_0}^2)\,{q_0}^2} - \mathrm{i}\sqrt{{p_0}^2\,(1-{q_0}^2)}\right) \\ p_0 q_0 - \sigma(p_0,q_0)\left(-\sqrt{(1-{p_0}^2)\,(1-{q_0}^2)} + \mathrm{i}\sqrt{(1-{p_0}^2)\,{q_0}^2} - \mathrm{i}\sqrt{{p_0}^2\,(1-{q_0}^2)}\right) \\ p_0 q_0 - \sigma(p_0,q_0)\left(-\sqrt{(1-{p_0}^2)\,(1-{q_0}^2)} - \mathrm{i}\sqrt{(1-{p_0}^2)\,{q_0}^2} + \mathrm{i}\sqrt{{p_0}^2\,(1-{q_0}^2)}\right) \end{array} \right\} , \qquad (16)$$

where the overall sign in the right-hand terms depends on the sign of $p_0 q_0 = (1/4)\,\mathrm{tr}\,R_4(p,q)$,

$$\sigma(p_0,q_0) = \mathrm{sign}(p_0 q_0) \; .$$

This feature is subtle, and arises in the process of removing a spurious apparent asymmetry between p_0 and q_0 in the eigenvalue expressions associated with the appearance of $\sqrt{{q_0}^2}$ and $\sqrt{{p_0}^2}$; incorrect signs arise in removing the square roots without $\sigma(p_0,q_0)$, which is required to make the determinant equal to the products of the eigenvalues. The eigenvectors can be computed in the usual way, but we know of no informative simple algebraic form. Interestingly, the eigensystem of the *profile matrix* of $R_4(p,q)$, discussed later in Section 4, is much simpler.

2. Double-Quaternion Approach to the 4D RMSD Problem

Here we present the nontrivial steps needed to understand and solve the 4D spatial and orientation-frame RMSD optimization problems in the quaternion framework. We extend our solutions for 4×4 symmetric, traceless profile matrices M_3 arising from 3D Euclidean data to the case of unconstrained 4×4 profile matrices M_4, which arise naturally for 4D Euclidean data.

While we might expect the quaternion eigensystem of the 4D profile matrix to allow us to solve the 4D RMSD problem in exactly the same fashion as in 3D, this is, interestingly, false. We will need several stages of analysis to actually find the correct way to exploit quaternions in the 4D RMSD optimization context. In this Section, we study the problem by itself, in a way that can be easily solved using a quaternion approach with the *numerical* methods traditional in the 3D problem. We devote the Appendix to a detailed treatment of the alternative *algebraic* solutions to the eigensystems of the 4×4 symmetric real matrices that are relevant to our quaternion-based spatial-coordinate and orientation-frame alignment problems in 3D and 4D.

2.1. Review of the Notation for the RMSD Problem

Our starting point for all alignment analysis is the minimization of the difference measure quantifying the rotational alignment of a D-dimensional set of point test data $\{x_k\}$ relative to a reference data set $\{y_k\}$,

$$\mathbf{S}_D = \sum_{k=1}^{N} \| R_D \cdot x_k - y_k \|^2 \; , \qquad (17)$$

which we replace by a maximization of its cross-term

$$\Delta_D = \sum_{k=1}^{N} (R_D \cdot x_k) \cdot y_k = \sum_{a=1,b=1}^{D} {R_D}^{ba} E_{ab} \; = \; \operatorname{tr} R_D \cdot E, \tag{18}$$

where E is the cross-covariance matrix

$$E_{ab} = \sum_{k=1}^{N} [x_k]_a \, [y_k]_b = \left[\mathbf{X} \cdot \mathbf{Y}^{\mathsf{T}}\right]_{ab} \; , \tag{19}$$

and $[x_k]$ denotes the kth column of $\mathbf{X}$.

For 3D data, we convert this to a quaternion matrix problem by applying Eq. (6) to get

$$\Delta(q) = \operatorname{tr} R_3(q) \cdot E = (q_0, q_1, q_2, q_3) \cdot M_3(E) \cdot (q_0, q_1, q_2, q_3)^{\mathsf{T}} \equiv q \cdot M_3(E) \cdot q \; , \tag{20}$$

Choosing the traditional 3D indexing $\{x, y, z\}$ for (a, b), the traceless, symmetric profile matrix takes the form

$$M_3(E) = \begin{bmatrix} E_{xx} + E_{yy} + E_{zz} & E_{yz} - E_{zy} & E_{zx} - E_{xz} & E_{xy} - E_{yx} \\ E_{yz} - E_{zy} & E_{xx} - E_{yy} - E_{zz} & E_{xy} + E_{yx} & E_{zx} + E_{xz} \\ E_{zx} - E_{xz} & E_{xy} + E_{yx} & -E_{xx} + E_{yy} - E_{zz} & E_{yz} + E_{zy} \\ E_{xy} - E_{yx} & E_{zx} + E_{xz} & E_{yz} + E_{zy} & -E_{xx} - E_{yy} + E_{zz} \end{bmatrix} . \tag{21}$$

The maximal measure is given by the eigensystem of the maximal eigenvalue ϵ_{opt} of M_3 and the corresponding quaternion eigenvector q_{opt}, with the result

$$\left. \begin{array}{rcl} \Delta_{\mathrm{opt}} & = & \operatorname{tr}[R_3(q_{\mathrm{opt}}) \cdot E] \\ & = & q_{\mathrm{opt}} \cdot M_3 \cdot q_{\mathrm{opt}} \\ & = & q_{\mathrm{opt}} \cdot \left(\epsilon_{\mathrm{opt}} \, q_{\mathrm{opt}}\right) \\ & = & \epsilon_{\mathrm{opt}} \end{array} \right\} . \tag{22}$$

2.2. Starting Point for the 4D RMSD Problem.

The 4D double quaternion matrix Eq. (15) provides the most general quaternion context that we know of for expressing an RMSD problem. We start with the RMSD minimization problem for 4D Euclidean point data expressed as the maximization problem for the by-now-familiar cross-term expression

$$\Delta_4 = \sum_{k=1}^{N} (R_4 \cdot x_k) \cdot y_k = \sum_{a=0,b=0}^{3} {R_4}^{ba} E_{4:ab} \; = \; \operatorname{tr} R_4 \cdot E_4, \tag{23}$$

where

$$E_{4:ab} = \sum_{k=1}^{N} [x_k]_a \, [y_k]_b = \left[\mathbf{X} \cdot \mathbf{Y}^{\mathsf{T}}\right]_{ab} \tag{24}$$

is the cross-covariance matrix whose (a, b) indices we will usually write as (w, x, y, z) in the manner of Eq. (21).

Using Eq. (15) in Eq. (23) to perform the 4D version of the rearrangement of the similarity function, we can rewrite our measure as

$$\Delta_4 = \operatorname{tr} R_4(p, q) \cdot E_4 \; = \; (p_0, p_1, p_2, p_3) \cdot M_4(E_4) \cdot (q_0, q_1, q_2, q_3)^{\mathsf{T}} \; \equiv \; p \cdot M_4(E_4) \cdot q \; , \tag{25}$$

where the profile matrix for the 4D data now becomes

$$M_4(E_4) = \begin{bmatrix} E_{ww}+E_{xx}+E_{yy}+E_{zz} & E_{yz}-E_{zy}-E_{wx}+E_{xw} & E_{zx}-E_{xz}-E_{wy}+E_{yw} & E_{xy}-E_{yx}-E_{wz}+E_{zw} \\ E_{yz}-E_{zy}+E_{wx}-E_{xw} & E_{ww}+E_{xx}-E_{yy}-E_{zz} & E_{xy}+E_{yx}-E_{wz}-E_{zw} & E_{zx}+E_{xz}+E_{wy}+E_{yw} \\ E_{zx}-E_{xz}+E_{wy}-E_{yw} & E_{xy}+E_{yx}+E_{wz}+E_{zw} & E_{ww}-E_{xx}+E_{yy}-E_{zz} & E_{yz}+E_{zy}-E_{wx}-E_{xw} \\ E_{xy}-E_{yx}+E_{wz}-E_{zw} & E_{zx}+E_{xz}-E_{wy}-E_{yw} & E_{yz}+E_{zy}+E_{wx}+E_{xw} & E_{ww}-E_{xx}-E_{yy}+E_{zz} \end{bmatrix} \tag{26}$$

and we note that, in contrast to $M_3(E_3)$, $M_4(E_4)$ is *neither* traceless nor symmetric.

2.3. A Tentative 4D Eigensystem

Our task is now to find an algorithm that allows us to successfully compute the quaternion pair $(p_{\rm opt}, q_{\rm opt})$, or, equivalently, the global rotation $R_4(p_{\rm opt}, q_{\rm opt})$, that maximizes the measure

$$\Delta_4 = \operatorname{tr} R_4(p,q)\cdot E_4 \;=\; p\cdot M_4(E_4)\cdot q\ , \tag{27}$$

with $M_4(E_4)$ a general real matrix with a generic trace and no symmetry conditions. Note that now we can have *both* left and right eigenvectors p and q for a single eigenvalue of the profile matrix M_4: q would correspond to the eigenvectors of M_4, and p would correspond to the eigenvectors of the transpose $M_4{}^{\rm T}$. *Warning:* The eigensystem of M_4 typically has some complex eigenvalues and is furthermore *insufficient* by itself to solve the 4D RMSD optimization problem, so additional refinements will be necessary. We now explore a path to an optimal solution amenable to quaternion-based numerical evaluation, with applicable algebraic approaches elaborated in the Appendix.

For some types of calculations, we may find it useful to decompose M_4 in a way that isolates particular features using the form

$$M_4(w,x,y,z,\ldots) = \begin{bmatrix} w+x+y+z & a-a_w & b-b_w & c-c_w \\ a+a_w & w+x-y-z & C-C_w & B+B_w \\ b+b_w & C+C_w & w-x+y-z & A-A_w \\ c+c_w & B-B_w & A+A_w & w-x-y+z \end{bmatrix}\ , \tag{28}$$

where $(w,x,y,z) = (E_{ww}, E_{xx}, E_{yy}, E_{zz})$, $a = E_{yz}-E_{zy}$, cyclic, $A = E_{yz}+E_{zy}$, cyclic, $a_w = E_{wx}-E_{xw}$, cyclic, $A_w = E_{wx}+E_{xw}$, cyclic, and $\operatorname{tr}(M_4) = 4w$. This effectively exposes the structural symmetries of M_4.

We next review the properties of the eigenvalue equation $\det[M_4 - eI_4] = 0$, where e is the variable we solve for to obtain the four eigenvalues ϵ_k, and I_4 denotes the 4D identity matrix; transposing M_4 does not change the eigenvalues but does interchange the distinct left and right eigenvectors. While M_4 itself has new properties, the corresponding expressions in terms of e and ϵ_k, along with the outcome of eliminating e (Abramowitz & Stegun, 1970), are by now familiar:

$$\det[M_4 - eI_4] \;=\; e^4 + e^3 p_1 + e^2 p_2 + e p_3 + p_4 \;=\; 0 \tag{29}$$

$$(e-\epsilon_1)(e-\epsilon_2)(e-\epsilon_3)(e-\epsilon_4) \;=\; 0 \tag{30}$$

$$\left.\begin{array}{lcl} p_1 &=& (-\epsilon_1-\epsilon_2-\epsilon_3-\epsilon_4) \\ p_2 &=& (\epsilon_1\epsilon_2+\epsilon_1\epsilon_3+\epsilon_2\epsilon_3+\epsilon_1\epsilon_4+\epsilon_2\epsilon_4+\epsilon_3\epsilon_4) \\ p_3 &=& (-\epsilon_1\epsilon_2\epsilon_3-\epsilon_1\epsilon_2\epsilon_4-\epsilon_1\epsilon_3\epsilon_4-\epsilon_2\epsilon_3\epsilon_4) \\ p_4 &=& \epsilon_1\epsilon_2\epsilon_3\epsilon_4 \end{array}\right\}\ . \tag{31}$$

We make no assumptions about M_4, so its structure includes a trace term $4w = -p_1$ as well as the possible antisymmetric components shown in Eq. (28), yielding the following expressions for the $p_k(E_4)$ following from the expansion of $\det[M_4 - eI_4]$:

$$p_1(E_4) \;=\; -\operatorname{tr}[M_4] = -4w \tag{32}$$

$$
\begin{aligned}
p_2(E_4) &= \frac{1}{2}\left(\mathrm{tr}\,[M_4]\right)^2 - \frac{1}{2}\,\mathrm{tr}\,[M_4 \cdot M_4] \\
&= 6w^2 - 2(x^2+y^2+z^2) - A^2 - a^2 - B^2 - b^2 - C^2 - c^2 \\
&\quad + {A_w}^2 + {a_w}^2 + {B_w}^2 + {b_w}^2 + {C_w}^2 + {c_w}^2 \qquad (33) \\
p_3(E_4) &= -\frac{1}{6}\left(\mathrm{tr}\,[M_4]\right)^3 + \frac{1}{2}\,\mathrm{tr}\,[M_4 \cdot M_4]\,\mathrm{tr}\,[M_4] - \frac{1}{3}\,\mathrm{tr}\,[M_4 \cdot M_4 \cdot M_4] \\
&= -8xyz + 4w(x^2+y^2+z^2) \\
&\quad - 2ABC - 2Abc - 2aBc - 2abC \\
&\quad + 2A^2x - 2a^2x + 2B^2y - 2b^2y + 2C^2z - 2c^2z \\
&\quad - 2AB_wC_w + 2Ab_wc_w - 2aB_wc_w + 2ab_wC_w \\
&\qquad - 2A_wBC_w + 2a_wBc_w - 2a_wbC_w + 2A_wbc_w \\
&\qquad - 2A_wB_wC + 2a_wb_wC - 2A_wb_wc + 2a_wB_wc \\
&\quad + 2a^2w + 2A^2w - 2A_w^2w - 2A_w^2x - 2a_w^2w + 2a_w^2x \\
&\qquad + 2b^2w + 2B^2w - 2B_w^2w - 2B_w^2y - 2b_w^2w + 2b_w^2y \\
&\qquad + 2c^2w + 2C^2w - 2C_w^2w - 2C_w^2z - 2c_w^2w + 2c_w^2z \qquad (34) \\
p_4(E_4) &= \det\,[M_4]\ . \qquad (35)
\end{aligned}
$$

2.4. Issues with the Naive 4D Approach

We previously found that we could maximize $\Delta_3 = \mathrm{tr}(R_3 \cdot E_3)$ over the 3D rotation matrices R_3 by mapping E_3 to the profile matrix M_3, with $\Delta_3 = q \cdot M_3 \cdot q$, solving for the maximal eigenvalue ϵ_{opt} of the symmetric matrix M_3, and choosing $R_{\mathrm{opt}} = R_3(q_{\mathrm{opt}})$ with q_{opt} the normalized quaternion eigenvector corresponding to $\Delta_3(\mathrm{opt}) = \epsilon_{\mathrm{opt}}$. The obvious 4D extension of the 3D quaternion RMSD problem would be to examine $\Delta_4 = \mathrm{tr}\,(R_4 \cdot E_4) = q_\lambda \cdot M_4 \cdot q_\rho$. This is defined over the 4D rotation matrices R_4, where M_4 in Eq. (26) turns out no longer to be symmetric, so we must split the eigenvector space into a separate left-quaternion q_λ and right-quaternion q_ρ. We might guess that as in the 3D case, M_4 would have a maximal eigenvalue ϵ_{opt} (already a problem – it may be complex), and we could use the “optimal” left and right eigenvectors $q_{\lambda:\mathrm{opt}}$ and $q_{\rho:\mathrm{opt}}$ that could be obtained as the corresponding eigenvectors of M_4 and ${M_4}^{\mathrm{T}}$. Then the solution to the 4D optimization problem would look like this:

$$\Delta_4(\mathrm{opt}) \stackrel{?}{=} q_{\lambda:\mathrm{opt}} \cdot M_4 \cdot q_{\rho:\mathrm{opt}} = (q_{\lambda:\mathrm{opt}} \cdot q_{\rho:\mathrm{opt}})\,\epsilon_{\mathrm{opt}}\ . \qquad (36)$$

Unfortunately, this is wrong. First, even when this result is real, Eq. (36) is typically smaller than the actual maximum of $\mathrm{tr}(R_4(q_\lambda, q_\rho) \cdot E_4)$ over the space of 4D rotation matrices (or their equivalent representations in terms of a search through q_λ and q_ρ). Even a simple *slerp* through q_{ID} and just beyond the apparent optimal eigenvectors $q_{\lambda:\mathrm{opt}}$ and $q_{\rho:\mathrm{opt}}$ from an eigenvalue of M_4 can yield *larger* values of Δ_4! And, to add insult to injury, starting with those eigenvectors $q_{\lambda:\mathrm{opt}}$ and $q_{\rho:\mathrm{opt}}$, one does not in general even find a *basis* for some normalized linear combination that yields the true optimal result. What is going wrong, and what is the path to our hoped-for quaternionic solution to the 4D RMSD problem, which seems so close to the 3D RMSD problem, but then fails so spectacularly to correspond to the obvious hypothesis?

2.5. Insights from the Singular Value Decomposition

We know that the 3D version of Eq. (36) is certainly correct with ϵ_{opt} the maximal eigenvalue of $M_3(E_3)$, and we know also that there is *some* rotation matrix $R_4(q_\lambda, q_\rho)$ that maximizes $\mathrm{tr}(R_4(q_\lambda, q_\rho) \cdot E_4)$, and therefore the 4D expression Eq. (36) must describe $\Delta_4(\mathrm{opt})$ for *some* non-trivial pair of quaternions (q_λ, q_ρ). The crucial issue is that the 3D RMSD problem and the 4D RMSD problem differ, with 3D being a special case due to the symmetry of the

4×4 profile matrix. We know also that the SVD form of the optimal rotation matrix is valid in *any* dimension, so we conjecture that the key is to look at the commonality of the SVD solutions in 3D and 4D, and work backwards to see how those non-quaternion-driven equations might relate to what we know is *in principle* a quaternion approach to the 4D problem that looks like Eq. (36).

Therefore, we first look at the general singular-value decomposition for the spatial alignment problem (Schönemann, 1966; Golub & van Loan, 1983) and then analyze the 3D and 4D problems to understand how we can recover a quaternion-based construction of the 4D spatial RMSD solution. For 3D and 4D, the basic SVD construction of the optimal rotation for a cross-covariance matrix E takes the form

$$\{U,\, S,\, V\} = \text{SingularValueDecomposition}\,(E) \tag{37}$$

where

$$E(U,S,V) = U \cdot S \cdot V^{\mathsf{T}} \tag{38}$$

$$R_{\text{opt}}(U,D,V) = V \cdot D \cdot U^{\mathsf{T}} \tag{39}$$

$$D_3 = \text{Diagonal}\left(1, 1, \operatorname{sign}\det(V \cdot U^{\mathsf{T}})\right) \tag{40}$$

$$D_4 = \text{Diagonal}\left(1, 1, 1, \operatorname{sign}\det(V \cdot U^{\mathsf{T}})\right) \;. \tag{41}$$

Here U and V are orthogonal matrices that are usually ordinary rotations, while D is usually the identity matrix but can be nontrivial in more situations than one might think. A critical component for this analysis is the diagonal matrix S, whose elements are the all-positive square roots of the eigenvalues of the symmetric matrix ${E_4}^{\mathsf{T}} \cdot E_4$ (the trace of this matrix is the squared Fröbenius norm of E). The first key fact is that in any dimension the RMSD cross-term obeys the following sequence of transformations following from the SVD relations of Eqs. (38) –(41):

$$\left.\begin{array}{rcl} \Delta(\text{opt}) \;=\; \operatorname{tr}(R_{\text{opt}} \cdot E) & = & \operatorname{tr}(R_{\text{opt}} \cdot [U \cdot S \cdot V^{\mathsf{T}}]) \\ & = & \operatorname{tr}\left([V \cdot D \cdot U^{\mathsf{T}}] \cdot [U \cdot S \cdot V^{\mathsf{T}}]\right) \\ & = & \operatorname{tr}(D \cdot S) \end{array}\right\} \;. \tag{42}$$

Note the appearance of D in the SVD formula for the optimal measure; we found in numerical experiments that including this term is absolutely essential to guaranteeing agreement with brute force verification of the optimization results, particularly in 4D.

3D Context. Thus an alternative to considering the 3D optimization of $\operatorname{tr}(R \cdot E)$ in the context of E alone is to look at the 3×3 matrices

$$\left.\begin{array}{rcl} F & = & E^{\mathsf{T}} \cdot E \\ F' & = & E \cdot E^{\mathsf{T}} \end{array}\right\} \tag{43}$$

and to note that, although E itself will not in general be symmetric, F and F' are intrinsically symmetric. Thus they have the same eigenvalues, and like all nonsingular matrices of this form, and unlike E itself, will have real positive eigenvalues (Golub & van Loan, 1983) that we can write as $(\gamma_1, \gamma_2, \gamma_3)$. From Eq. (38), we can show that $\operatorname{tr} F = \operatorname{tr} F' = \operatorname{tr}(S \cdot S)$, and since the trace is the sum of the eigenvalues, the eigensystem of F or F' determines S. The diagonal elements that enter naturally into the SVD are therefore just the square roots

$$S(E) = \text{Diagonal}\left(\sqrt{\gamma_1}, \sqrt{\gamma_2}, \sqrt{\gamma_3}\right) \;. \tag{44}$$

So far, this has no obvious connection to the quaternion system. For our next step, let us now examine how the 3D SVD system relates to the profile matrix $M_3(E_3)$ derived from the quaternion decomposition to give the form in Eq. (21). We define the analogs of Eq. (43) for a profile matrix as

$$\left.\begin{array}{rcl} G & = & M^{\mathsf{T}} \cdot M \\ G' & = & M \cdot M^{\mathsf{T}} \end{array}\right\} \;, \tag{45}$$

where we recall that in 3D, $\epsilon_{\rm opt}$ is just the maximal eigenvalue of $M_3(E)$. Thus if we arrange the eigenvalues of $M_3(E)$ in descending order as $(\epsilon_1, \epsilon_2, \epsilon_3, \epsilon_4)$, we obviously have

$$\text{Eigenvalues}(G) = \text{Eigenvalues}(G') = (\alpha_1, \alpha_2, \alpha_3, \alpha_4) = ({\epsilon_1}^2, {\epsilon_2}^2, {\epsilon_3}^2, {\epsilon_4}^2) \ . \tag{46}$$

Therefore, since we already know that $\epsilon_1(M) = \Delta(\text{opt})$, we have precisely the sought-for connection,

$$\sqrt{\text{Max Eigenvalue}(G)} = \sqrt{\alpha_1} = \text{tr}(D \cdot S) = \Delta(\text{opt}) = \epsilon_1(M) \ . \tag{47}$$

That is, given E, compute $M(E)$ from the quaternion decomposition, and, instead of examining the eigensystem of $M(E)$ itself, take the square root of the maximal eigenvalue of the manifestly symmetric, positive-definite real matrix $G = M^{\mathsf{T}} \cdot M$. This is the quaternion-based translation of the 3D application of the SVD method to obtaining the optimal rotation: numerical methods in particular do not care whether you are computing the maximal eigenvalue of a symmetric quaternion-motivated matrix M_3 or of the associated symmetric matrix ${M_3}^{\mathsf{T}} \cdot M_3$.

Note: In 3D, we can compute *all four* of the eigenvalues of G from the *three* elements of S (Coutsias *et al.*, 2004): defining

$$\text{Diagonal}(D \cdot S) = (\lambda_1, \lambda_2, \lambda_3) \ , \tag{48}$$

then we can write

$$\begin{bmatrix} \alpha_1 \\ \alpha_2 \\ \alpha_3 \\ \alpha_4 \end{bmatrix} = \begin{bmatrix} (+\lambda_1 + \lambda_2 + \lambda_3)^2 \\ (-\lambda_1 - \lambda_2 + \lambda_3)^2 \\ (-\lambda_1 + \lambda_2 - \lambda_3)^2 \\ (+\lambda_1 - \lambda_2 - \lambda_3)^2 \end{bmatrix} \ , \tag{49}$$

where obviously $\sqrt{\alpha_1} = \text{tr}(D \cdot S)$ is maximal.

The final step is to connect $R_3(\text{opt})$ to a quaternion via $R_3(q_{\rm opt})$ without requiring prior knowledge of the SVD solution Eq. (39). We know that the square root of the maximal eigenvalue of $G = M^{\mathsf{T}} \cdot M$, which depends only on the quaternion decomposition, gives us $\text{tr}(D \cdot S) = \Delta(\text{opt})$ without using the SVD, and we know that in 3D the profile matrix M is symmetric, so G and G' share a single maximal eigenvector v corresponding to $\alpha_1 = (\text{tr}(D \cdot S))^2 = (\Delta(\text{opt}))^2$. Using this eigenvector we thus have

$$v \cdot G \cdot v = (M \cdot v)^{\mathsf{T}} \cdot (M \cdot v) = v \cdot \left((\text{tr}(D \cdot S))^2 \, v\right) = (\Delta(\text{opt}))^2 \ ,$$

so in this case $v = q_{\rm opt}$ is itself the optimal eigenvector determining $R_3(q_{\rm opt})$.

4D Context. The 4D case, as we are now aware, cannot be solved using the non-symmetric profile matrix $M_4(E_4)$ directly. But now we can see a more general way to exploit the 4D quaternion decomposition of Eq. (26) by constructing the *manifestly symmetric products*

$$\left. \begin{array}{rcl} G & = & {M_4}^{\mathsf{T}} \cdot M_4 \\ G' & = & M_4 \cdot {M_4}^{\mathsf{T}} \end{array} \right\} \ . \tag{50}$$

Although this superficially extends Eq. (45) to 4D, it is quite different because M_4 is not itself symmetric (as M_3 was), and so, while G and G' have the same eigenvalues, they have *distinct eigenvectors* q_ρ and q_λ, respectively. If we use the maximal eigenvalue α_1 to solve for q_ρ and q_λ as follows, these in fact will produce the optimal quaternion system. First we solve these equations using the maximal eigenvalue α_1 of G,

$$\left. \begin{array}{rcccl} G \cdot q_\rho & = & \alpha_1 \, q_\rho & = & (\text{tr}(D \cdot S))^2 \, q_\rho \\ G' \cdot q_\lambda & = & \alpha_1 \, q_\lambda & = & (\text{tr}(D \cdot S))^2 \, q_\lambda \end{array} \right\} \ . \tag{51}$$

At this point, the *signs* of the eigenvectors have to be checked for a correction, since the eigenvector is still correct whatever its sign or scale. But we know that the value of $q_\lambda \cdot M_4(E_4) \cdot q_\rho$ must be positive, so we simply check that sign, and change, say, $q_\lambda \to -q_\lambda$ if needed to make the sign positive. There is still an *overall* sign ambiguity, but that is natural and an intrinsic part of the rotation $R_4(q_\lambda, q_\rho)$, so now we can use these eigenvectors to generate the optimal measure for the 4D translational RMSD problem using *only* the quaternion-based data, giving finally the whole spectrum of ways to write $\Delta_4(\text{opt})$:

$$\Delta_4(\text{opt}) = \text{tr}(R_{4:\text{opt}}(q_\lambda, q_\rho) \cdot E_4) = q_\lambda \cdot M_4(E_4) \cdot q_\rho = \sqrt{\alpha_1} \; . \tag{52}$$

Note: In 4D, we can compute all the eigenvalues of G from the *four* elements of S: defining

$$\text{Diagonal}(D \cdot S) = (\lambda_1, \lambda_2, \lambda_3, \lambda_4) \; , \tag{53}$$

then we can write

$$\begin{bmatrix} \alpha_1 \\ \alpha_2 \\ \alpha_3 \\ \alpha_4 \end{bmatrix} = \begin{bmatrix} (+\lambda_1 + \lambda_2 + \lambda_3 + \lambda_4)^2 \\ (+\lambda_1 + \lambda_2 - \lambda_3 - \lambda_4)^2 \\ (+\lambda_1 - \lambda_2 + \lambda_3 - \lambda_4)^2 \\ (+\lambda_1 - \lambda_2 - \lambda_3 + \lambda_4)^2 \end{bmatrix} \; , \tag{54}$$

where again $\sqrt{\alpha_1} = \text{tr}(D \cdot S)$ is maximal.

Summary: Now we have the entire algorithm for solving the RMSD spatial alignment problem in 4D by exploiting the quaternion decomposition of Eq. (25) and Eq. (26), based on Eq. (15), inspired by, but in no way dependent upon knowing, the SVD solution to the problem:

- **Compute the profile matrix.** Using the quaternion decomposition Eq. (15) of the general 4D rotation matrix $R_4(p, q)$, extract the 4D profile matrix $M_4(E_4)$ of Eq. (26) from the initial proximity measure

$$\Delta_4 = \text{tr}(R_4(p, q) \cdot E_4) = p \cdot M_4(E_4) \cdot q \; . \tag{55}$$

  So far all we know is the numerical value of M_4 and the fact the Δ_4 can be maximized by exploring the entire space of the quaternion pair $(p, \, q)$.
- **Construct the symmetric matrices and extract the optimal eigenvalue.** The maximal eigenvalue α_1 of the 4×4 symmetric matrix $G = {M_4}^{\mathsf{T}} \cdot M_4$ is itself easily obtained by numerical means, just as one has done traditionally for M_3. If all we need is the optimal value of the proximity measure for comparison, we are done:

$$\Delta_4(\text{opt}) \; = \; \sqrt{\text{Max Eigenvalue}\left(G = {M_4}^{\mathsf{T}} \cdot M_4\right)} \; = \; \sqrt{\alpha_1} \; . \tag{56}$$

  The alternative algebraic methods for computing the eigenvalues are discussed in the Appendix.
- **If needed, compute the left and right eigenvectors of G:** Our two distinct symmetric matrices, $G = {M_4}^{\mathsf{T}} \cdot M_4$ and $G' = M_4 \cdot {M_4}^{\mathsf{T}}$ have their own distinct maximal eigenvectors, both corresponding to the maximal eigenvalue α_1 shared by G and G', so we can easily use this common maximal numerical eigenvalue to solve

$$\left.\begin{array}{rcl} (G - \alpha_1 I_4) \cdot q_{\text{opt}:\rho} & = & 0 \\ (G' - \alpha_1 I_4) \cdot q_{\text{opt}:\lambda} & = & 0 \end{array}\right\} \tag{57}$$

  for the numerical values of $q_{\text{opt}:\lambda}$ and $q_{\text{opt}:\rho}$. We correct the signs so that $q_{\text{opt}:\lambda} \cdot M_4(E_4) \cdot q_{\text{opt}:\rho} > 0$, and then these in turn yield the required 4D rotation matrix

$$R_{4:\text{opt}}\left(q_{\text{opt}:\lambda}, \, q_{\text{opt}:\rho}\right)$$

  from Eq. (15).

If everything is in order, all of the following ways of expressing $\Delta_4(\text{opt})$ should now be equivalent,

$$\Delta_4(\text{opt}) \;=\; \text{tr}\big(R_{4:\text{opt}}\left(q_{\text{opt}:\lambda},\, q_{\text{opt}:\rho}\right)\cdot E_4\big) \;=\; q_{\text{opt}:\lambda}\cdot M_4(E_4)\cdot q_{\text{opt}:\rho} \;=\; \sqrt{\alpha_1}\;, \tag{58}$$

independently of the fact that one knows from the SVD decomposition of E_4 that $\Delta_4(\text{opt}) = \text{tr}(D\cdot S) = \sqrt{\alpha_1}$.

3. 4D Orientation-Frame Alignment

In this section, we review and slightly expand the details of the 3D orientation-frame in the main text. Then we extend that treatment to handle the case of 4D orientation-frame alignment to complete the picture we started in Section 2 on the 4D spatial frame alignment problem. A detailed evaluation of the accuracy of the 3D chord measure compared to the arc-length measure, along with other questions, is given separately in Section 6.

3.1. Details of the 3D Orientation-Frame alignment Problem

We first review the basic structure of our 3D orientation-frame method and then proceed to present some additional details.

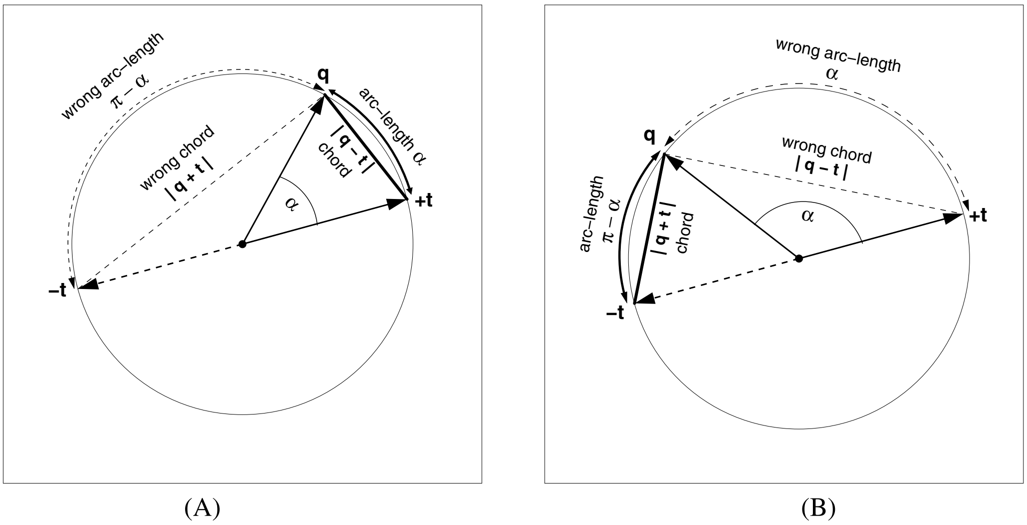

Figure 1
Geometric context involved in choosing a *quaternion distance* that will result in the correct *average rotation matrix* when the quaternion measures are optimized. Because the quaternion vectors represented by t and $-t$ give the same rotation matrix, one must choose $|\cos\alpha|$ or the *minima*, that is $\min(\alpha,\, \pi-\alpha)$ or $\min(\|q-t\|,\, \|q+t\|)$, of the alternative distance measures to get the *correct* items in the arc-length or chord measure summations. (A) and (B) represent the cases when the first or second choice should be made, respectively.

Review of Orientation Frames in 3D. The ideal optimization problem for 3D orientation frames requires a measure constructed from the geodesic arc lengths on the quaternion hypersphere. Starting with the bare angle between two quaternions on $\mathbf{S}^3$, $\alpha = \arccos(q_1 \cdot q_2)$, where we recall that $\alpha \geq 0$, we define a *pseudometric* (Huynh, 2009) for the geodesic arc-length distance as

$$d_{\text{geodesic}}(q_1, q_2) = \min(\alpha,\ \pi - \alpha) : \ \ 0 \leq d_{\text{geodesic}}(q_1, q_2) \leq \frac{\pi}{2}\ , \tag{59}$$

as illustrated in Fig. (1). An efficient implementation of this is to take

$$d_{\text{geodesic}}(q_1, q_2) = \arccos(|q_1 \cdot q_2|)\ , \tag{60}$$

which we now exploit to construct a measure from geodesic arc-lengths on the quaternion hypersphere instead of Euclidean distances in space. Thus to compare a test quaternion-frame data set $\{p_k\}$ to a reference data set $\{r_k\}$, we employ the geodesic-based least squares measure

$$\mathbf{S}_{\text{geodesic}} \ = \ \sum_{k=1}^{N} \left(\arccos|(q \star p_k) \cdot r_k|\right)^2 \ = \ \sum_{k=1}^{N} \left(\arccos|q \cdot (r_k \star \bar{p}_k)|\right)^2\ , \tag{61}$$

where the alternative second form follows from Eq. (4).

Since this does not easily fit into a linear algebra approach to construct optimal solutions to the orientation-frame alignment problem, we choose to approximate the measure of Eq. (61) by the linearizable *chord distance* measure, which does, under certain conditions, permit a valid closed form solution. We take as our approximate measure the chordal pseudometric (Huynh, 2009; Hartley *et al.*, 2013),

$$d_{\text{chord}}(q_1, q_2) = \min(\|q_1 - q_2\|,\ \|q_1 + q_2\|) : \ \ 0 \leq d_{\text{chord}}(q_1, q_2) \leq \sqrt{2}\ . \tag{62}$$

We compare the geometric origins for Eq. (60) and Eq. (62) in Fig. (1). Note that the crossover point between the two expressions in Eq. (62) is at $\pi/2$, so the hypotenuse of the right isosceles triangle at that point has length $\sqrt{2}$.

The solvable approximate optimization function analogous to $\|R\cdot x - y\|^2$ that we will now explore for the quaternion-frame alignment problem will thus take the form that must be minimized as

$$\mathbf{S}_{\text{chord}} = \sum_{k=1}^{N} \left(\min(\|(q \star p_k) - r_k\|,\ \|(q \star p_k) + r_k\|)\right)^2\ . \tag{63}$$

We can convert the sign ambiguity in Eq. (63) to a deterministic form like Eq. (60) by observing, with the help of Fig. (1), that

$$\|q_1 - q_2\|^2 = 2 - 2q_1 \cdot q_2, \qquad \|q_1 + q_2\|^2 = 2 + 2q_1 \cdot q_2\ . \tag{64}$$

Clearly $(2 - 2|q_1 \cdot q_2|)$ is always the smallest of the two values. Thus minimizing Eq. (63) amounts to maximizing the now-familiar cross-term form, which we can write as

$$\left.\begin{array}{rcl} \Delta_{\text{chord}}(q) & = & \sum_{k=1}^{N} |(q \star p_k) \cdot r_k| \\ & = & \sum_{k=1}^{N} |q \cdot (r_k \star \bar{p}_k)| \\ & = & \sum_{k=1}^{N} |q \cdot t_k| \end{array}\right\}\ . \tag{65}$$

Here we have used the identity $(q \star p) \cdot r = q \cdot (r \star \bar{p})$ from Eq. (4) and defined the quaternion displacement or "attitude error" (Markley *et al.*, 2007)

$$t_k = r_k \star \bar{p}_k\ . \tag{66}$$

Note that we could have derived the same result using Eq. (3) to show that $\|q \star p - r\| = \|q \star p - r\| \|p\| = \|q - r \star \bar{p}\|$.

The final step is to choose the samples of q that include our expected optimal quaternion, and adjust the sign of each data value t_k to $\tilde{t}_k$ by the transformation

$$\tilde{t}_k = t_k \ \mathrm{sign}(q \cdot t_k) \quad \rightarrow |q \cdot t_k| = q \cdot \tilde{t}_k \ . \tag{67}$$

The neighborhood of q matters because, as argued by (Hartley *et al.*, 2013), even though the allowed range of 3D rotation angles is $\theta \in (-\pi, \pi)$ (or quaternion sphere angles $\alpha \in (-\pi/2, \pi/2)$), convexity of the optimization problem cannot be guaranteed for collections outside local regions centered on some θ_0 of size $\theta_0 \in (-\pi/2, \pi/2)$ (or $\alpha_0 \in (-\pi/4, \pi/4)$): beyond this range, local basins may exist that allow the mapping Eq. (67) to produce distinct local variations in the assignments of the $\{\tilde{t}_k\}$ and in the solutions for q_{opt}. Within considerations of such constraints, Eq. (67) now allows us to take the summation outside the absolute value, and write the quaternion-frame optimization problem in terms of maximizing the cross-term expression

$$\left.\begin{array}{rcl} \Delta_{\mathrm{chord}}(q) & = & \displaystyle\sum_{k=1}^{N} q \cdot \tilde{t}_k \\ & = & q \cdot V(t) \end{array}\right\} \tag{68}$$

where $V = \sum_{k=1}^{N} \tilde{t}_k$ is the analog of the Euclidean RMSD profile matrix M. However, since this is *linear* in q, we have the remarkable result that, as noted in the treatment of (Hartley *et al.*, 2013) regarding the quaternion L_2 chordal-distance norm, the solution is immediate. We have simply

$$q_{\mathrm{opt}} = \frac{V}{\|V\|} \ , \tag{69}$$

since that immediately maximizes the value of $\Delta_{\mathrm{chord}}(q)$ in Eq. (68). This gives the maximal value of the measure as

$$\Delta_{\mathrm{chord}}(q_{\mathrm{opt}}) = \|V\| \ , \tag{70}$$

and thus $\|V\|$ is the exact orientation frame analog of the spatial RMSD maximal eigenvalue ϵ_{opt}, except it is far easier to compute.

Alternative chord-measure approach parallel to the Euclidean case. Having understood the chordal distance approach for the orientation-alignment problem in terms of the pseudometric Eq. (62) and the measure Eq. (65) transformed into the form Eq. (68) involving the corrected quaternion displacements $\{\tilde{t}_k\}$, we now observe that we can also express the problem in a form much closer to our Euclidean RMSD optimization problem. Returning to the form

$$\mathbf{S}_{\mathrm{chord}} = \sum_{k=1}^{N} \|q \star p_k - r_k\|^2 \ . \tag{71}$$

we see that we can effectively transform the sign of *only* $p_k \rightarrow \tilde{p}_k$ using the same test as Eq. (67) to make Eq. (71) valid as it stands; we then proceed, in the same fashion as the spatial alignment problem but with the modification required by Eq. (65), to convert to a cross-term form as follows:

$$\begin{array}{rcl} \Delta_{\mathrm{chord}}(q) & = & \displaystyle\sum_{k=1}^{N} |(q \star p_k) \cdot r_k| \ = \ \sum_{k=1}^{N} (q \star \tilde{p}_k) \cdot r_k \\ & = & \displaystyle\sum_{a=0,b=0}^{3} Q(q)_{ba} \sum_{k=1}^{N} [\tilde{p}_k]_a \ [r_k]_b \\ & = & \mathrm{tr}\, Q(q) \cdot W \ . \end{array} \tag{72}$$

Here W is essentially a cross-covariance matrix in the quaternion data elements and $Q(q)$ is the quaternion matrix of Eq. (1). Since $Q(q)$ is linear in q, we can simply pull out their coefficients, yielding

$$\Delta_{\text{chord}}(q) = q \cdot V(W) \ , \tag{73}$$

where V is a four-vector corresponding to the profile matrix in the spatial problem:

$$V(W) = \begin{bmatrix} +W_{00} + W_{11} + W_{22} + W_{33} \\ +W_{01} - W_{10} + W_{23} - W_{32} \\ +W_{02} - W_{20} + W_{31} - W_{13} \\ +W_{03} - W_{30} + W_{12} - W_{21} \end{bmatrix} . \tag{74}$$

This is of course exactly the same as the quaternion difference transformation Eq. (66), expressed as a profile matrix transformation, and Eq. (73) leads, assuming consistent data localization, to the same optimal unit quaternion

$$q_{\text{opt}} = \frac{V}{\|V\|} \ , \tag{75}$$

that maximizes the value of Δ_{chord} in Eq. (68), and the maximal value of the measure is again $\Delta_{\text{chord}}(q_{\text{opt}}) = \|V\|$.

Matrix Form of the Linear Vector Chord Distance. While Eq. (68) (or Eq. (73)) does not immediately fit into the eigensystem-based RMSD matrix method used in the spatial problem, it can in fact be easily transformed from a system linear in q to an equivalent matrix system *quadratic* in q. Since any power of the optimization measure will yield the same extremal solution, we can simply *square* the right-hand side of Eq. (68) and write the result in the form

$$\begin{aligned} \Delta_{\text{chord-sq}} &= (q \cdot V)(q \cdot V) \\ &= \sum_{a=0,b=0}^{3} q_a \, V_a V_b \, q_b \\ &= q \cdot \Omega \cdot q \ , \end{aligned} \tag{76}$$

where $\Omega_{ab} = V_a V_b$ is a 4×4 symmetric matrix with $\det \Omega = 0$, and $\operatorname{tr} \Omega = \sum_a {V_a}^2 \neq 0$. The eigensystem of Ω is just defined by the eigenvalue $\|V\|^2$, and combination with the spatial eigensystem can be achieved either numerically or algebraically using the trace $\neq 0$ case of our quartic solution. The process differs dramatically from what we did with Δ_{chord}, but the forms of the eigenvectors are necessarily *identical*. Thus it is in fact possible to merge the QFA system for Δ_{chord} into the matrix method of the spatial RMSD using Eq. (76).

Fixing Sign Problem with Quadratic Rotation Matrix Chord Distance. However, there is another approach that has a very natural way to incorporate manifestly *sign-independent* quaternion chord distances into our general context, and which has a very interesting close relationship to Δ_{chord}. The method begins with the observation that full 3D rotation matrices like Eq. (6) can be arranged to rotate the set of frames of the $\{p_k\}$ to be as close as possible to the reference frame $\{r_k\}$ by employing a measure that is a particular product of rotation matrices. The essence is to notice that the trace of any 3D rotation matrix expressed in axis-angle form (rotation about a fixed axis $\hat{\mathbf{n}}$ by θ) can be expressed in two equivalent forms:

$$\operatorname{tr} R(\theta, \hat{\mathbf{n}}) = 1 + 2\cos\theta \tag{77}$$

$$\operatorname{tr} R(q) = 3{q_0}^2 - {q_1}^2 - {q_2}^2 - {q_3}^2 = 4{q_0}^2 - 1 \ , \tag{78}$$

and therefore traces of rotation matrices can be turned into maximizable functions of the angles appearing in the trace. Noting that the squared Fröbenius norm of a matrix M is the trace $\operatorname{tr} M \cdot M^{\mathsf{T}}$, we begin with the goal of minimizing a Fröbenius norm of the form

$$\|R(q) \cdot R(p_k) - R(r_k)\|^2_{\text{Frob.}} \ ,$$

and then convert from a minimization problem in this norm to a maximization of the cross-term as usual. The result is, remarkably, an explicitly symmetric and traceless profile matrix in the quaternions. We thus begin with this form of the orientation-frame measure (see, e.g., (Huynh, 2009; Moakher, 2002; Hartley *et al.*, 2013)),

$$\begin{aligned}\Delta_{\mathrm{RRR}} &= \sum_{k=1}^{N} \operatorname{tr}\left[R(q)\cdot R(p_k)\cdot R^{-1}(r_k)\right] = \sum_{k=1}^{N} \operatorname{tr}\left[R(q\star p_k\star \bar{r}_k)\right] \\ &= \sum_{k=1}^{N} \operatorname{tr}\left[R(q)\cdot R(p_k\star \bar{r}_k)\right] = \sum_{k=1}^{N} \operatorname{tr}\left[R(q)\cdot R^{-1}(r_k\star \bar{p}_k)\right] ,\end{aligned} \quad (79)$$

where $\bar{r}$ denotes the complex conjugate or inverse quaternion. We note that due to the correspondence of Δ_{RRR} with a cosine measure (via Eq. (77)), this must be *maximized* to find the optimal q, so both Δ_{chord} and Δ_{RRR} correspond naturally to the cross-term measure we used for Euclidean point data, which we will later refer to as Δ_x when necessary to distinguish it.

We next observe that the formulas for Δ_{RRR} and the pre-summation arguments of Δ_{chord} are related as follows:

$$\sum_{k=1}^{N} \operatorname{tr}\left[R(q)\cdot R(p_k)\cdot R(\bar{r}_k)\right] = \sum_{k=1}^{N} \left(4\left((q\star p_k)\cdot r_k\right)^2 - (q\cdot q)(p_k\cdot p_k)(r_k\cdot r_k)\right) , \quad (80)$$

where of course the last term reduces to a constant since we apply the unit-length constraint to all the quaternions, but is algebraically essential to the construction. The odd form of Eq. (80) is not a typographical error: the conjugate $\bar{r}$ of the reference data must be used in the $R\cdot R\cdot R$ expression, and the ordinary r must be used in both terms on the right-hand. We conclude that using the $R\cdot R\cdot R$ measure and replacing the argument of Δ_{chord} by its square *before* summing over k are equivalent maximizing measures that eliminate the quaternion sign dependence. Now using the quaternion triple-term identity $(q\star p)\cdot r = q\cdot(r\star\bar{p})$ of Eq. (4), we see that each term of Δ_{RRR} reduces to a quaternion product that is a quaternion difference, or a "quaternion displacement" $t_k = r_k\star\bar{p}_k$, i.e., the rotation mapping each individual test frame to its corresponding reference frame,

$$\left.\begin{aligned}\Delta_{\mathrm{RRR}} = \sum_{k=1}^{N} \operatorname{tr}\left[R(q)\cdot R(p_k)\cdot R(\bar{r}_k)\right] &= \sum_{k=1}^{N} \left(4\left((q\star p_k)\cdot r_k\right)^2 - (q\cdot q)(p_k\cdot p_k)(r_k\cdot r_k)\right) \\ &= \sum_{k=1}^{N} \left(4\left(q\cdot(r_k\star\bar{p}_k)\right)^2 - 1\right) \\ &= 4\sum_{a,b} q_a \left(\sum_{k=1}^{N} [t_k]_a\,[t_k]_b\right) q_b - N \\ &= 4\,q\cdot A(t)\cdot q - N .\end{aligned}\right\} \quad (81)$$

Here the 4×4 matrix $A(t)_{ab} = \sum_{k=1}^{N}[t_k]_a\,[t_k]_b$ is the alternative (equivalent) profile matrix that was introduced by (Markley *et al.*, 2007; Hartley *et al.*, 2013) for the chord-based *quaternion-averaging* problem. We can therefore use either the measure Δ_{RRR} or

$$\Delta_{\mathrm{A}} = q\cdot A(t)\cdot q \quad (82)$$

as our rotation-matrix-based sign-insensitive chord-distance optimization measure. Exactly like our usual spatial measure, these measures must be *maximized* to find the optimal q. It is, however, important to emphasize that the optimal quaternion will *differ* for the Δ_{chord} , $\Delta_{\mathrm{chord\text{-}sq}}$, and $\Delta_{\mathrm{RRR}} \sim \Delta_{\mathrm{A}}$ measures, though they will normally be very similar. More details are explored in Section 6.

Details of Rotation Matrix Form. We now recognize that the sign-insensitive measures are all very closely related to our original spatial RMSD problem, and all can be solved by finding the optimal quaternion eigenvector $q_{\rm opt}$ of a 4×4 matrix. The procedure for $\Delta_{\text{chord-sq}}$ and $\Delta_{\rm A}$ follows immediately, but it is useful to work out the options for $\Delta_{\rm RRR}$ in a little more detail.

Choosing Eq. (79) has the remarkable feature of producing, via Eq. (6) for $R(q)$, an expression *quadratic* in q, with a symmetric, traceless profile matrix $U(p,r)$ that is *quartic* in the quaternion elements p_k and r_k. This variant of the chord-based QFA problem thus falls into the same category as the standard RMSD problem, and permits the application of the same exact solution (or, indeed, the traditional numerical solution method if that is more efficient). The profile matrix equation is unwieldy to write down explicitly in terms of the quaternion elements quartic in $\{p, r\}$, but we actually have several options for expressing the content in a simpler form. One is to write the matrices in abstract canonical 3×3 form, e.g.,

$$R(p) = [P] = \begin{bmatrix} p_{xx} & p_{xy} & p_{xz} \\ p_{yx} & p_{yy} & p_{yz} \\ p_{zx} & p_{zy} & p_{zz} \end{bmatrix} , \tag{83}$$

where the *columns* of this matrix are just the three axes of each data element's frame triad. This is often exactly what our original data look like, for example, if the residue orientation frames of a protein are computed from cross-products of atom-atom vectors (Hanson & Thakur, 2012). Then we can define for each data element the 3×3 matrix

$$[T_k] = R(p_k) \cdot R(\bar{r}_k)] = R(p_k \star \bar{r}_k) = R^{-1}(t_k) ,$$

so we can write T either in terms of a 3×3 matrix like Eq. (83) derived from the actual frame-column data, or in terms of Eq. (6) and the quaternion frame data $t_k = r_k \star \bar{p}_k$. We then may write the frame measure in general as

$$\Delta_{\rm RRR} = \sum_{k=1}^{N} \operatorname{tr}\left(R(q) \cdot T_k\right) = \sum_{a=1,b=1}^{3} R_{ba}(q) T_{ab} , \tag{84}$$

where the frame-based cross-covariance matrix is simply $T_{ab} = \sum_{k=1}^{N} [T_k]_{ab}$. As before, we can easily expand $R(q)$ using Eq. (6) to convert the measure to a 4D linear algebra problem of the form

$$\Delta_{\rm RRR} = \sum_{a=0,b=0}^{3} q_a \cdot U_{ab}(p,r) \cdot q_b = q \cdot U(p,r) \cdot q . \tag{85}$$

Here $U(p,r) = U(T)$ has the same relation to T as $M(E)$ does to E in Eq. (21). We may choose to write the profile matrix $U = \sum_k U_k$ appearing in $\Delta_{\rm RRR}$ either in terms of the individual k-th components of the numerical 3D rotation matrix $T = R^{-1}(t)$ or using the composite quaternion $t = r \star \bar{p}$:

$$U_k(T) \equiv U(t_k)$$
$$= \begin{bmatrix} T_{xx}+T_{yy}+T_{zz} & T_{yz}-T_{zy} & T_{zx}-T_{xz} & T_{xy}-T_{yx} \\ T_{yz}-T_{zy} & T_{xx}-T_{yy}-T_{zz} & T_{xy}+T_{yx} & T_{xz}+T_{zx} \\ T_{zx}-T_{xz} & T_{xy}+T_{yx} & -T_{xx}+T_{yy}-T_{zz} & T_{yz}+T_{zy} \\ T_{xy}-T_{yx} & T_{xz}+T_{zx} & T_{yz}+T_{zy} & -T_{xx}-T_{yy}+T_{zz} \end{bmatrix}_k \tag{86}$$

$$= \begin{bmatrix} 3{t_0}^2-{t_1}^2-{t_2}^2-{t_3}^2 & 4t_0t_1 & 4t_0t_2 & 4t_0t_3 \\ 4t_0t_1 & -{t_0}^2+3{t_1}^2-{t_2}^2-{t_3}^2 & 4t_1t_2 & 4t_1t_3 \\ 4t_0t_2 & 4t_1t_2 & -{t_0}^2-{t_1}^2+3{t_2}^2-{t_3}^2 & 4t_2t_3 \\ 4t_0t_3 & 4t_1t_3 & 4t_2t_3 & -{t_0}^2-{t_1}^2-{t_2}^2+3{t_3}^2 \end{bmatrix}_k . \tag{87}$$

Both Eq. (86) and Eq. (87) are *quartic* (and identical) when expanded in terms of the quaternion data $\{p_k, r_k\}$. To compute the necessary 4×4 numerical profile matrix U, one need only substitute the appropriate 3D frame triads or their corresponding quaternions for the kth frame pair and sum over k . Since the orientation-frame profile matrix U is symmetric and traceless just like the Euclidean profile matrix M, the same solution methods for the optimal quaternion rotation q_{opt} will work without alteration in this case, which is probably the preferable method for the general problem.

Evaluation. The details of evaluating the properties of our quaternion-frame alignment algorithms, and especially comparing the chord approximation to the arc-length measure, are tedious and are available separately in Section 6. The top-level result is that, even for quite large rotational differences, the mean differences between the arc-length measure's numerical optimal angle and the various chord approximations are on the order of a fraction of a degree.

3.2. The 4D Orientation-Frame alignment Problem

Orientation frames in four dimensions have axes that are the columns of a 4D rotation matrix taking the identity frame to the new orientation frame. Therefore, in parallel with the 3D case, such frames can be represented either as 4D rotation matrices (the action on a 4D identity frame to get a new set of 4 orthogonal axes), or as the pair of quaternions (q, q') used in Eq. (15) to define $R_4(q, q')$. As in the 3D frame case, we will take advantage of the chord-distance linearization of the geodesic angular measure, and we shall present two alternative approaches to the optimization measure.

Quadratic Form. In 3D, with Eq. (68) having a single quaternion involved in the rotation, we were able to write down Δ_{chord} in terms of a simple expression linear in the quaternion q and the cumulative data V, and we observed that a quadratic expression $(q \cdot V)^2$ would also produce the same optimal eigenvector $q = V/\|V\|$. The optimal frame problem in 4D, in contrast, already requires a pair of quaternions, and one strategy is to split the analogs of the 3D quadratic expression into two parts, yielding

$$\Delta_{4:\mathrm{chord\text{-}sq}}(q, q') = (q \cdot V)\,(q' \cdot V') = q_a\,(V_a V'_b)\,q'_b = q \cdot \Omega_4 \cdot q' \tag{88}$$

as the generalization from 3D to 4D. Here, each 4D test frame consists of frames denoted by the quaternion pair (p, p'), and each reference frame employs a pair (r, r'), so we build the data coefficients starting from

$$\left.\begin{array}{rcl} V &=& \displaystyle\sum_{k=1}^{N} (r_k \star \bar{p}_k) = \textstyle\sum_{k=1}^{N} t_k \\ V' &=& \displaystyle\sum_{k=1}^{N} (r'_k \star \bar{p}'_k) = \textstyle\sum_{k=1}^{N} t'_k \end{array}\right\} \tag{89}$$

and then applying the transformation

$$\left.\begin{array}{rcl} t_k \to \tilde{t}_k &=& t_k\ \mathrm{sign}(q \cdot t_k) \\ t'_k \to \tilde{t'}_k &=& t'_k\ \mathrm{sign}(q' \cdot t'_k) \end{array}\right\} \tag{90}$$

to achieve consistent (local) signs. According to Eq. (74), V could also be constructed from $W_{ab} = \sum_{k=1}^{N} [\tilde{p}_k]_a\ [r_k]_b$, and V' from $W'_{ab} = \sum_{k=1}^{N} [\tilde{p'}_k]_a\ [r'_k]_b$, noting that here p is transformed by the "tilde" of Eq. (90). Now, for the 4D frame pairs, the solution for the optimal quaternions must achieve the maximum for *both* elements of the pair, and so

we obtain as a solution maximizing Eq. (88)

$$\left.\begin{array}{rcl} q_{\rm opt} & = & \dfrac{V}{\|V\|} \\ q'_{\rm opt} & = & \dfrac{V'}{\|V'\|} \\ \Delta_{\text{4:chord-sq}}(\text{opt}) & = & \|V\|\|V'\| \end{array}\right\} . \tag{91}$$

Remark: There is a particular reason to prefer Eq. (88) for the 4D orientation frame problem: in the next section, we will see that the separate *pre-summation* arguments for V and V', gathered together, are *exactly* equal to the joint summand of the 4D triple rotation pre-summation arguments, following the pattern seen in Eq. (80) for the 3D orientation-frame analysis.

Quartic Triple Rotation Form. One can also eliminate the sign choice step altogether by defining a 4D frame similarity measure that is the exact analog of Eq. (79) in 3D as follows:

$$\Delta_{\text{RRR4}} = \sum_{k=1}^{N} \operatorname{tr}\left[R(q,q')\cdot R(p_k,{p'}_k)\cdot R^{-1}(r_k,{r'}_k)\right] \tag{92}$$

$$= \sum_{k=1}^{N} \operatorname{tr}\left[R(q,q')\cdot R(p_k\star\bar{r}_k,\ {p'}_k\star\bar{r}'_k)\right] \tag{93}$$

$$= \sum_{k=1}^{N} \operatorname{tr}\left[R(q,q')\cdot R^{-1}(t_k,\ t'_k)\right] \tag{94}$$

$$= q\cdot U(p,p';\, r,r')\cdot q' . \tag{95}$$

Remarkably, there is a 4D version of the 3D identity Eq. (80) relating the triple rotation measure to the quadratic realizations of the linear quaternion rotation measures, namely

$$\left.\begin{array}{rcl} \displaystyle\sum_{k=1}^{N} \operatorname{tr}\left[R(q,q')\cdot R(p_k,p'_k)\cdot R(\bar{r}_k,\bar{r}'_k)\right] & = & \displaystyle 4\sum_{k=1}^{N}\left((q\star p_k)\cdot r_k\right)\left((q'\star p'_k)\cdot r'_k\right) \\ & = & \displaystyle 4\sum_{k=1}^{N}\left(q\cdot(r_k\star\bar{p}_k)\right)\left(q'\cdot(r'_k\star\bar{p}'_k)\right) \\ & = & \displaystyle 4\sum_{k=1}^{N}(q\cdot t_k)(q'\cdot t'_k) \\ & = & \displaystyle 4\sum_{a,b} q_a\left(\sum_{k=1}^{N}[t_k]_a\,[t'_k]_b\right)q'_b \\ & = & 4\,q\cdot A(t=r\star\bar{p},\ t'=r'\star\bar{p}')\cdot q' \end{array}\right\} , \tag{96}$$

Thus the pre-summation version of the arguments in the $(q\cdot V)(q'\cdot V')$ version of the 4D chord measure turns out to be *exactly* the same as the triple-matrix product measure summand without the additional trace term that is present in 3D. Furthermore, as long as one follows the rules of changing *both* the primed and unprimed signs together (the condition for $R_4(q,q')$'s invariance), this measure is sign-independent. The 4×4 matrix $A(t,t')$ is the 4D profile matrix

equivalent to that of (Markley *et al.*, 2007; Hartley *et al.*, 2013) for the 3D chord-based quaternion-averaging problem. We can therefore use either the measure Δ_{RRR4} or

$$\Delta_{\mathrm{A4}} = q \cdot A(t,t') \cdot q' \tag{97}$$

with $A(t,t')_{ab} = \sum_{k=1}^{N} [t_k]_a \, [t'_k]_b$ as our rotation-matrix-based sign-insensitive chord-distance optimization measure.

To get an expression in terms of R, we now use Eq. (15) for $R(q,q')$ to decompose the measure Eq. (93) into the rotation-averaging form

$$\Delta_{\mathrm{RRR4}} = \operatorname{tr}\left[R(q,q') \cdot T(p,p';r,r')\right] \tag{98}$$

$$= q \cdot U(T) \cdot q' \,, \tag{99}$$

where $T(p,p';r,r') = \sum_{k=1}^{N} R^{-1}(t_k,\, t'_k)$ and $U(T)$ has the same relationship to T as the 4D profile matrix $M(E)$ in Eq. (26) does to the cross-correlation matrix E. In the next section, we will see that the singleton version of this map is unusually degenerate, with rank one, though that feature does not persist for data sets with $N > 1$.

Now, as in the 4D spatial RMSD analysis, we might naturally assume that we could follow the 3D case by determining the maximal eigenvalue ϵ_0 of U and its left and right eigenvectors q_λ and q_ρ, which would give

$$\Delta_{\mathrm{RRR4}} \stackrel{?}{=} q_\lambda \cdot U \cdot q_\rho = (q_\lambda \cdot q_\rho)\, \epsilon_0 \,.$$

As before, this is not a maximal value for the measure Δ_{RRR4} over the possible range of $R(q,q')$. To solve the optimization correctly, we must again be very careful, and work with the maximal eigenvalue $\alpha(\mathrm{RRR4{:}opt})$ of $G = U^{\top} \cdot U$ and $G' = U \cdot U^{\top}$, which we can get numerically as usual, or algebraically from the quartic solution for the eigenvalues for symmetric 4×4 matrices with a trace, yielding

$$\Delta_{\mathrm{RRR4}}(\mathrm{opt}) = \sqrt{\text{max eigenvalue } (U^{\top} \cdot U)} = \sqrt{\alpha(\mathrm{RRR4{:}opt})} \,.$$

If we need the actual optimal rotation matrix solving

$$\Delta_{\mathrm{RRR4}}(\mathrm{opt}) = \operatorname{tr}\left(R_4(q_{\mathrm{opt}}, q'_{\mathrm{opt}}) \cdot S\right) = q_{\mathrm{opt}} \cdot U \cdot q'_{\mathrm{opt}} = \sqrt{\alpha(\mathrm{RRR4{:}opt})} \,,$$

then we just use our optimal eigenvalue to solve

$$\begin{aligned} (G - \alpha(\mathrm{RRR4{:}opt}) I_4) \cdot q &= 0 \\ (G' - \alpha(\mathrm{RRR4{:}opt}) I_4) \cdot q' &= 0 \end{aligned}$$

for q_{opt} and q'_{opt}, or use the equivalent adjugate-column method to extract the eigenvectors. That gives the desired 4D rotation matrix $R_4(q_{\mathrm{opt}}, q'_{\mathrm{opt}})$ explicitly via Eq. (15). The same approach applies to the solution of $\Delta_{\mathrm{A4}} = q \cdot A(t,t') \cdot q'$, Note that this can all be accomplished numerically, directly as above or with Singular Value Decomposition, or using the quaternion eigenvalue decomposition on the symmetric matrices either numerically or algebraically,

4. On Obtaining Quaternions and Quaternion Pairs from 3D and 4D Rotation Matrices

4.1. Extracting a Quaternion from 3D Rotation Matrices

The quaternion RMSD profile matrix method can be used to implement a singularity-free algorithm to obtain the (sign-ambiguous) quaternions corresponding to numerical 3D and 4D rotation matrices. There are many existing approaches to the 3D problem in the literature (see, e.g., (Shepperd, 1978), (Shuster & Natanson, 1993), or Section 16.1 of (Hanson, 2006)). In contrast to these approaches, Bar-Itzhack (Bar-Itzhack, 2000) has observed, in essence, that if we simply replace the data matrix E_{ab} by a numerical 3D orthogonal rotation matrix R, the numerical quaternion q that corresponds to $R_{\text{numeric}} = R(q)$, as defined by Eq. (6), can be found by solving our familiar maximal quaternion eigenvalue problem. The initially unknown optimal matrix (technically its quaternion) computed by maximizing the similarity measure turns out to be computable as a single-element quaternion barycenter problem. To see this, take $S(r)$ to be the sought-for optimal rotation matrix, with its own quaternion r, that must maximize the Bar-Itzhack measure. We start with the Fröbenius measure describing the match of two rotation matrices corresponding to the quaternion r for the unknown quaternion and the numeric matrix R containing the known 3×3 rotation matrix data:

$$\begin{aligned} \mathbf{S}_{\text{BI}} &= \|S(r) - R\|^2_{\text{Frob}} = \operatorname{tr}\left([S(r) - R] \cdot [S^{\top}(r) - R^{\top}]\right) \\ &= \operatorname{tr}\left(I_3 + I_3 - 2\left(S(r) \cdot R^{\top}\right)\right) \\ &= \text{const} - 2 \operatorname{tr} S(r) \cdot R^{\top} . \end{aligned}$$

Pulling out the cross-term as usual and converting to a maximization problem over the unknown quaternion r, we arrive at

$$\Delta_{\text{BI}} = \operatorname{tr} S(r) \cdot R^{\top} = r \cdot K(R) \cdot r , \tag{100}$$

where R is (approximately) an orthogonal matrix of numerical data, and $K(R)$ is analogous to the profile matrix $M(E)$. Since both S and R are $\mathbf{SO}(3)$ rotation matrices, so is their product $T = S \cdot R^{\top}$, and thus that product itself corresponds to some axis $\hat{\mathbf{n}}$ and angle θ, where

$$\operatorname{tr} S(r) \cdot R^{\top}(q) = \operatorname{tr} T(r \star \bar{q}) = \operatorname{tr} T(\theta, \hat{\mathbf{n}}) = 1 + 2\cos\theta .$$

The maximum is obviously close to the ideal value $\theta = 0$, which corresponds to $S \approx R$. Thus if we find the maximal quaternion eigenvalue ϵ_{opt} of the profile matrix $K(R)$ in Eq. (100), our closest solution is well-represented by the corresponding normalized quaternion eigenvector r_{opt},

$$q = r_{\text{opt}} . \tag{101}$$

This numerical solution for q will correspond to the targeted numerical rotation matrix, solving the problem. To complete the details of the computation, we replace the elements E_{ab} in Eq. (21) by a general orthonormal rotation matrix with columns $\mathbf{X} = (x_1, x_2, x_3)$, $\mathbf{Y}$, and $\mathbf{Z}$, scaling by 1/3, thus obtaining the special 4×4 profile matrix K whose elements in terms of a known numerical matrix $R = [\mathbf{X}|\mathbf{Y}|\mathbf{Z}]$ (transposed in the algebraic expression for K due to the $R^{\top}$ in Δ_{BI}) are

$$K(R) = \frac{1}{3}\begin{bmatrix} x_1 + y_2 + z_3 & y_3 - z_2 & z_1 - x_3 & x_2 - y_1 \\ y_3 - z_2 & x_1 - y_2 - z_3 & x_2 + y_1 & x_3 + z_1 \\ z_1 - x_3 & x_2 + y_1 & -x_1 + y_2 - z_3 & y_3 + z_2 \\ x_2 - y_1 & x_3 + z_1 & y_3 + z_2 & -x_1 - y_2 + z_3 \end{bmatrix} . \tag{102}$$

Determining the algebraic eigensystem of Eq. (102) is a nontrivial task. However, as we know, any orthogonal 3D rotation matrix $R(q)$, or equivalently, $R^{\mathrm{T}}(q) = R(\bar{q})$, can also be ideally expressed in terms of quaternions via Eq. (6), and this yields an alternate useful algebraic form

$$K(q) = \frac{1}{3}\begin{bmatrix} 3q_0^2 - q_1^2 - q_2^2 - q_3^2 & 4q_0q_1 & 4q_0q_2 & 4q_0q_3 \\ 4q_0q_1 & -q_0^2 + 3q_1^2 - q_2^2 - q_3^2 & 4q_1q_2 & 4q_1q_3 \\ 4q_0q_2 & 4q_1q_2 & -q_0^2 - q_1^2 + 3q_2^2 - q_3^2 & 4q_2q_3 \\ 4q_0q_3 & 4q_1q_3 & 4q_2q_3 & -q_0^2 - q_1^2 - q_2^2 + 3q_3^2 \end{bmatrix} \quad (103)$$

This equation then allows us to quickly prove that K has the correct properties to solve for the appropriate quaternion corresponding to R. First we note that the coefficients p_n of the eigensystem are simply constants,

$$p_1 = 0 \qquad p_2 = -\tfrac{2}{3} \qquad p_3 = -\tfrac{8}{27} \qquad p_4 = -\tfrac{1}{27} \quad .$$

Computing the eigenvalues and eigenvectors using the symbolic quaternion form, we see that the eigenvalues are constant, with maximal eigenvalue exactly one, and the eigenvectors are almost trivial, with the maximal eigenvector being the quaternion q that corresponds to the (numerical) rotation matrix:

$$\epsilon = \{1, -\frac{1}{3}, -\frac{1}{3}, -\frac{1}{3}\} \quad (104)$$

$$r = \left\{ \begin{bmatrix} q_0 \\ q_1 \\ q_2 \\ q_3 \end{bmatrix}, \begin{bmatrix} -q_1 \\ q_0 \\ 0 \\ 0 \end{bmatrix}, \begin{bmatrix} -q_2 \\ 0 \\ q_0 \\ 0 \end{bmatrix}, \begin{bmatrix} -q_3 \\ 0 \\ 0 \\ q_0 \end{bmatrix} \right\} . \quad (105)$$

The first column is the quaternion r_{opt}, with $\Delta_{\mathrm{BI}}(r_{\mathrm{opt}}) = 1$. (This would be 3 if we had not divided by 3 in the definition of K.)

Alternate version. From the quaternion barycenter work of Markley et al. (Markley *et al.*, 2007), we know that Eq. (103) actually has a much simpler form with the same unit eigenvalue and natural quaternion eigenvector. (This form appears naturally below in the 4D extension of the Bar-Itzhack algorithm.) If we simply take Eq. (103) multiplied by 3, add the constant term $I_4 = (q_0^2 + q_1^2 + q_2^2 + q_3^2)I_4$, and divide by 4, we get a more compact quaternion form of the matrix, namely

$$K'(q) = \begin{bmatrix} q_0^2 & q_0q_1 & q_0q_2 & q_0q_3 \\ q_0q_1 & q_1^2 & q_1q_2 & q_1q_3 \\ q_0q_2 & q_1q_2 & q_2^2 & q_2q_3 \\ q_0q_3 & q_1q_3 & q_2q_3 & q_3^2 \end{bmatrix} . \quad (106)$$

This has vanishing determinant and trace $\operatorname{tr} K' = 1 = -p_1$, with all other p_k coefficients vanishing, and eigensystem with eigenvalues identical to Eq. (103):

$$\epsilon = \{1, 0, 0, 0\} \quad (107)$$

$$r = \left\{ \begin{bmatrix} q_0 \\ q_1 \\ q_2 \\ q_3 \end{bmatrix}, \begin{bmatrix} -q_1 \\ q_0 \\ 0 \\ 0 \end{bmatrix}, \begin{bmatrix} -q_2 \\ 0 \\ q_0 \\ 0 \end{bmatrix}, \begin{bmatrix} -q_3 \\ 0 \\ 0 \\ q_0 \end{bmatrix} \right\} . \quad (108)$$

As elegant as this is, in practice, our numerical input data are from the 3×3 matrix R itself, and not the quaternions, so we will almost always just use those numbers in Eq. (102) to solve the problem.

Completing the solution. In typical applications, *the solution is immediate, requiring only trivial algebra*. The maximal eigenvalue is always known in advance to be unity for any valid rotation matrix, so we need only to compute the eigenvector from the numerical matrix Eq. (102) with unit eigenvalue. We simply compute any column of the adjugate matrix of $K(R) - I_4$, or solve the equivalent linear equations of the form

$$(K(R) - 1 * I_4) \cdot \begin{bmatrix} 1 \\ v_1 \\ v_2 \\ v_3 \end{bmatrix} = 0 \qquad q = r_{\text{opt}} = \text{normalize} \begin{bmatrix} 1 \\ v_1 \\ v_2 \\ v_3 \end{bmatrix} . \tag{109}$$

As always, one may need to check for degenerate special cases.

Non-ideal cases. It is important to note, as emphasized by Bar-Itzhack, that if there are *significant errors* in the numerical matrix R, then the actual non-unit maximal eigenvalue of $K(R)$ can be computed numerically or algebraically as usual, and then that eigenvalue's eigenvector determines the *closest* normalized quaternion to the errorful rotation matrix, which can be very useful since such a quaternion always produces a valid rotation matrix.

In any case, *up to an overall sign*, r_{opt} is the desired numerical quaternion q corresponding to the target numerical rotation matrix $R = R(q)$. In some circumstances, one is looking for a uniform statistical distribution of quaternions, in which case the overall sign of q should be chosen *randomly*.

The Bar-Itzhack approach solves the problem of extracting the quaternion of an arbitrary numerical 3D rotation matrix in a fashion that involves no singularities and only trivial testing for special cases, thus essentially making the traditional methods obsolete.

4.2. Extracting Quaternion Pairs from 4D Rotation Matrices

We know from Eq. (15) that any 4D orthogonal matrix $R_4(p,q)$ can be expressed as a quadratic form in two independent unit quaternions. This is a consequence of the fact that the 6-parameter orthogonal group $\mathbf{SO}(4)$ is double covered by the composition of two smaller 3-parameter unitary groups, that is $\mathbf{SU}(2) \times \mathbf{SU}(2)$; the group $\mathbf{SU}(2)$ has essentially the same properties as a single quaternion, so it is not surprising that $\mathbf{SO}(4)$ should be related to a pair of quaternions.

We begin our treatment of the 4D case by extending Eq. (100) to 4D with a numerical $\mathbf{SO}(4)$ matrix R_4, giving us a Bar-Itzhack measure to maximize of the form

$$\Delta_{4:\text{BI}} = \operatorname{tr} S(\ell, r) \cdot {R_4}^{\mathsf{T}} = \ell \cdot K_4(R_4) \cdot r = \ell \cdot K_4(p,q) \cdot r \, . \tag{110}$$

Here (ℓ, r) are the left and right quaternions over which we are varying the measure, and $K_4(R_4)$ is the 4D generalization of Eq. (102). To compute $K_4(R_4)$, we define a general 4D orthonormal rotation matrix with columns $\mathbf{W} = (w_0, w_1, w_2, w_3)$, etc., so the matrix takes the form $R_4 = [\mathbf{W}|\mathbf{X}|\mathbf{Y}|\mathbf{Z}]$, producing a numerical profile matrix of the form (taking into account the transpose in Eq. (110))

$$K_4(R_4) = \frac{1}{4} \begin{bmatrix} w_0 + x_1 + y_2 + z_3 & -w_1 + x_0 + y_3 - z_2 & -w_2 - x_3 + y_0 + z_1 & -w_3 + x_2 - y_1 + z_0 \\ w_1 - x_0 + y_3 - z_2 & w_0 + x_1 - y_2 - z_3 & -w_3 + x_2 + y_1 - z_0 & w_2 + x_3 + y_0 + z_1 \\ w_2 - x_3 - y_0 + z_1 & w_3 + x_2 + y_1 + z_0 & w_0 - x_1 + y_2 - z_3 & -w_1 - x_0 + y_3 + z_2 \\ w_3 + x_2 - y_1 - z_0 & -w_2 + x_3 - y_0 + z_1 & w_1 + x_0 + y_3 + z_2 & w_0 - x_1 - y_2 + z_3 \end{bmatrix} . \tag{111}$$

Now, from Eq. (15), we know that we also have an analog to Eq. (103), and for $R_4(p,q)$ this takes the remarkably compact algebraic form

$$K_4(p,q) = \begin{bmatrix} p_0q_0 & p_0q_1 & p_0q_2 & p_0q_3 \\ p_1q_0 & p_1q_1 & p_1q_2 & p_1q_3 \\ p_2q_0 & p_2q_1 & p_2q_2 & p_2q_3 \\ p_3q_0 & p_3q_1 & p_3q_2 & p_3q_3 \end{bmatrix} . \tag{112}$$

We see that Eq. (112) is exactly the outer product of p and q, with vanishing determinant, rank one, and a lone eigenvalue equal to its trace $(p \cdot q)$. Its deceptively beautiful simple eigensystem is

$$\begin{aligned}
\epsilon &= \{p \cdot q,\ 0,\ 0,\ 0\} && (113)\\
r_{\text{right}} &= \left\{ \begin{bmatrix} p_0 \\ p_1 \\ p_2 \\ p_3 \end{bmatrix}, \begin{bmatrix} -q_1 \\ q_0 \\ 0 \\ 0 \end{bmatrix}, \begin{bmatrix} -q_2 \\ 0 \\ q_0 \\ 0 \end{bmatrix}, \begin{bmatrix} -q_3 \\ 0 \\ 0 \\ q_0 \end{bmatrix} \right\} && (114)\\
\ell_{\text{left}} &= \left\{ \begin{bmatrix} q_0 \\ q_1 \\ q_2 \\ q_3 \end{bmatrix}, \begin{bmatrix} -p_1 \\ p_0 \\ 0 \\ 0 \end{bmatrix}, \begin{bmatrix} -p_2 \\ 0 \\ p_0 \\ 0 \end{bmatrix}, \begin{bmatrix} -p_3 \\ 0 \\ 0 \\ p_0 \end{bmatrix} \right\} . && (115)
\end{aligned}$$

However, we have seen this before in Section 2: the maximal eigensystems of *non-symmetric* 4D matrices are *not optimal.* We can in fact see that Eqs. (114) and (115) give $\Delta_{4:\mathrm{BI}} = (p \cdot q)^2$, whereas we know that ideally we must have a value that corresponds to the identity matrix, or eigenvalue unity. That is, the right and left eigenvectors in Eqs. (114) and (115) appear to be *reversed*.

The key to fixing this is now familiar: we must construct the *symmetric* matrices

$$G(p,q) = K_4^{\mathsf{T}} \cdot K_4 = \begin{bmatrix} {q_0}^2 & q_0 q_1 & q_0 q_2 & q_0 q_3 \\ q_0 q_1 & {q_1}^2 & q_1 q_2 & q_1 q_3 \\ q_0 q_2 & q_1 q_2 & {q_2}^2 & q_2 q_3 \\ q_0 q_3 & q_1 q_3 & q_2 q_3 & {q_3}^2 \end{bmatrix},\ G'(p,q) = K_4 \cdot K_4^{\mathsf{T}} = \begin{bmatrix} {p_0}^2 & p_0 p_1 & p_0 p_2 & p_0 p_3 \\ p_0 p_1 & {p_1}^2 & p_1 p_2 & p_1 p_3 \\ p_0 p_2 & p_1 p_2 & {p_2}^2 & p_2 p_3 \\ p_0 p_3 & p_1 p_3 & p_2 p_3 & {p_3}^2 \end{bmatrix}. \tag{116}$$

These both have a lone eigenvalue equal to one, that is $\epsilon = \operatorname{tr} G = \operatorname{tr} G' = 1$, and eigenvectors that are the *reverse* of Eqs. (114) and (115), that is

$$\left.\begin{aligned} r_{\text{right}} &= q \\ \ell_{\text{left}} &= p \end{aligned}\right\} . \tag{117}$$

Note: One can easily see the subtle emergence of the eigensystems of G and G' from the following calculation:

$$\begin{aligned} G \cdot q = &\ K_4^{\mathsf{T}} \cdot K_4 \cdot q &= K_4^{\mathsf{T}} \cdot (q \cdot q) p &= (p \cdot p)(q \cdot q) q = q \\ G' \cdot p = &\ K_4 \cdot K_4^{\mathsf{T}} \cdot p &= K_4 \cdot (p \cdot p) q &= (q \cdot q)(p \cdot p) p = p \end{aligned} .$$

So q and p are clearly the right and left quaternion vectors that maximize $\Delta_{4:\mathrm{BI}}$, with eigenvalue unity, always greater than or equal to the false candidate eigenvalue $(p \cdot q)$.

To solve the problem, we thus use our numerical data, e.g., the 4D rotation matrix data in Eq. (111), and compute the right and left normalized numerical eigenvectors q and p from G and G', respectively, assuming eigenvalue equal to one, and use those to optimally describe $R_4(p,q)$. Again, if a statistical distribution in the double quaternion space is desired, the signs can be chosen randomly, consistent with the sign of $\operatorname{tr} K_4(R_4)$. Explicitly, we can either use any (normalized) adjugate column or just solve some permutation of the following linear equations directly for the eigenvectors. *No further computation is required.*

$$(G(R) - 1 * I_4) \cdot \begin{bmatrix} 1 \\ v_1 \\ v_2 \\ v_3 \end{bmatrix} = 0 \qquad r_{\text{opt}} = q = \text{normalize} \begin{bmatrix} 1 \\ v_1 \\ v_2 \\ v_3 \end{bmatrix} \tag{118}$$

$$(G'(R) - 1 * I_4) \cdot \begin{bmatrix} 1 \\ v'_1 \\ v'_2 \\ v'_3 \end{bmatrix} = 0 \qquad \ell_{\mathrm{opt}} = p = \text{normalize} \begin{bmatrix} 1 \\ v'_1 \\ v'_2 \\ v'_3 \end{bmatrix} . \tag{119}$$

The solution to our problem is thus $R_4(p,q) = R_4(\ell_{\mathrm{opt}}, r_{\mathrm{opt}})$. As in 3D, if the numerical matrix R_4 has some moderate errors and the numerical maximum eigenvalues of (G, G') *differ* significantly from unity, we can solve for the actual maximal eigenvalues and insert those into Eqs. (118) and (119) to find the left and right eigenvectors numerically.

There is one important caveat: the 3D quaternion rotation $R_3(q)$ does not care what the sign of q is, but the 4D quaternion rotation $R_4(p,q)$ is only invariant under *both* $p \to -p$ and $q \to -q$ in tandem. To ensure that $R_4(p,q)$ is the same matrix, the signs of the quaternions must be adjusted after the initial computation so that the sign of $(\ell_{\mathrm{opt}} \cdot r_{\mathrm{opt}})$ matches the sign of the numerical input value of $R_{4(1,1)} = \mathrm{tr}\, K_4(R_4)$, which corresponds in the ideal case to $p \cdot q$. That guarantees that the solution describes the same matrix that we used as input, and not its negative.

5. Two-Dimensional Limit of 3D Problem

All rotations of the type we have been trying to optimize reduce to a rotation in a 2D plane, which in 3D is defined by the plane perpendicular to the eigenvector $\hat{\mathbf{n}}$ of the rotation matrix Eq. (6). Data sets that are highly linear, determining a robust straight line from least squares, can even circumvent the RMSD problem entirely: a very good rotation matrix can be calculated from the direction $\hat{\mathbf{x}}$ determined by the line fitted to the data set $\{x_i\}$, and the similar direction $\hat{\mathbf{y}}$ corresponding to the reference data set $\{y_i\}$. An optimal rotation matrix in 3D is then simply

$$R(\theta, \hat{\mathbf{n}}) = R(\arccos(\hat{\mathbf{x}} \cdot \hat{\mathbf{y}}), \widehat{\hat{\mathbf{x}} \times \hat{\mathbf{y}}}) , \tag{120}$$

which is easily generalized to any dimension by isolating just the projections of vectors to the plane determined by $\hat{\mathbf{x}}$ and $\hat{\mathbf{y}}$, and rotating in that 2D basis. Thus we conclude that, in general, if we had access to a prescient preconditioning rotation of the proper form, the entire RMSD problem would reduce to a very simple rotation in some 2D plane parameterized by a single angle. We can simulate this, giving a massively simpler set of expressions, by assuming the data are coplanar, all having $z = 0$ (or more conditions in higher dimensions) and thus lying in the canonical $\{\hat{\mathbf{x}}, \hat{\mathbf{y}}\}$ plane, for example. This reduces our fundamental RMSD profile matrix Eq. (21) for M to

$$M_{z=0} = \begin{bmatrix} d & 0 & 0 & c \\ 0 & D & C & 0 \\ 0 & C & -D & 0 \\ c & 0 & 0 & -d \end{bmatrix} , \tag{121}$$

where $d = E_{xx} + E_{yy}$, $D = E_{xx} - E_{yy}$, $c = E_{xy} - E_{yx}$, and $C = E_{xy} + E_{yx}$. Then $p_2 = -c^2 - C^2 - d^2 - D^2$, $p_3 = 0$, and $p_4 = (c^2 + d^2)(C^2 + D^2)$, and similarly for the other cyclic cases, $x = 0$ and $y = 0$. The p_2 and p_4 are obviously functions of only two variables, $u = c^2 + d^2$ and $v = C^2 + D^2$, so we can write in general $p_2 = -u - v$ and $p_4 = uv$. The eigenvalue equation $\det[M - eI_4] = e^4 + e^3 p_1 + e^2 p_2 + e p_3 + p_4 = 0$ reduces to $e^4 + e^2 p_2 + p_4 = 0$ and the eigenvalues become $\epsilon = (\sqrt{u}, \sqrt{v}, -\sqrt{v}, -\sqrt{u})$, while, as an initial form, we may write the normalized (quaternion)

eigenvectors as

$$q = \left\{ \begin{bmatrix} \frac{d+\sqrt{u}}{\sqrt{c^2+\left(d+\sqrt{u}\right)^2}} \\ 0 \\ 0 \\ \frac{c}{\sqrt{c^2+\left(d+\sqrt{u}\right)^2}} \end{bmatrix}, \begin{bmatrix} 0 \\ \frac{D+\sqrt{v}}{\sqrt{C^2+\left(D+\sqrt{v}\right)^2}} \\ \frac{C}{\sqrt{C^2+\left(D+\sqrt{v}\right)^2}} \\ 0 \end{bmatrix}, \begin{bmatrix} 0 \\ \frac{D-\sqrt{v}}{\sqrt{C^2+\left(D-\sqrt{v}\right)^2}} \\ \frac{C}{\sqrt{C^2+\left(D-\sqrt{v}\right)^2}} \\ 0 \end{bmatrix}, \begin{bmatrix} \frac{d-\sqrt{u}}{\sqrt{c^2+\left(d-\sqrt{u}\right)^2}} \\ 0 \\ 0 \\ \frac{c}{\sqrt{c^2+\left(d-\sqrt{u}\right)^2}} \end{bmatrix} \right\} . \tag{122}$$

However, there is an important simplification that can be made; we observe that we can write the 2D part of the leading eigenvector (the one of interest to the optimization problem with largest eigenvalue $\epsilon = \sqrt{u}$) as

$$q = \begin{bmatrix} \sqrt{\frac{\sqrt{u}+d}{2\sqrt{u}}} \\ \operatorname{sign} c \sqrt{\frac{\sqrt{u}-d}{2\sqrt{u}}} \end{bmatrix} = \begin{bmatrix} a \\ b \end{bmatrix} = \begin{bmatrix} \cos(\theta/2) \\ \sin(\theta/2) \end{bmatrix} . \tag{123}$$

(*Note* that the $(\operatorname{sign} c)$ coefficient, a remnant of the c appearing in Eq. (122), is absolutely essential, and the eigenvalue equations are not satisfied without it.) The leading quaternion eigenvalue then corresponds, using the 2D limit of Eq. (6), to the optimal rotation in the $\{\hat{\mathbf{x}}, \hat{\mathbf{y}}\}$ plane given by

$$R_2 = \begin{bmatrix} \frac{d}{\sqrt{u}} & -\frac{c}{\sqrt{u}} \\ \frac{c}{\sqrt{u}} & \frac{d}{\sqrt{u}} \end{bmatrix} = \begin{bmatrix} a^2 - b^2 & -2ab \\ 2ab & a^2 - b^2 \end{bmatrix} = \begin{bmatrix} \cos\theta & -\sin\theta \\ \sin\theta & \cos\theta \end{bmatrix} . \tag{124}$$

Yet Another Form. However, we have neglected something. How does this look if we simply go back to the data matrices for 2D? Let us first write down the 2D version of Eq. (17), taking $E_{ab} = \sum_{k=1}^{N} [x_k]_a \, [y_k]_b$ for $a, b = \{1, 2\}$. Let us assume that we do not yet know the solution for θ given in Eq. (124) above, so the raw form for the spatial RMSD task is to determine the unknown rotation matrix

$$R_2(\theta) = \begin{bmatrix} \cos\theta & -\sin\theta \\ \sin\theta & \cos\theta \end{bmatrix}$$

starting from the 2D cross-covariance data E_{ab}, maximizing

$$\begin{aligned} \Delta_2 = \textstyle\sum_{k=1}^{N} (R_2 \cdot x_k) \cdot y_k = \sum_{a=1,b=1}^{2} {R_2}^{ba} E_{ab} &= (E_{xx} + E_{yy}) \cos\theta + (E_{xy} - E_{yx}) \sin\theta \\ &= d\cos\theta + c\sin\theta \end{aligned} . \tag{125}$$

We can either differentiate with respect to θ and set $\Delta_2'(\theta) = 0$, or simply observe directly that $\Delta_2(\theta)$ is largest when the vector $(\cos\theta, \sin\theta)$ is parallel to its coefficients; both arguments lead to the solution

$$\tan\theta = \frac{E_{xy} - E_{yx}}{E_{xx} + E_{yy}} = \frac{c}{d} \tag{126}$$

$$(\cos\theta,\ \sin\theta) = \left(\frac{d}{\sqrt{c^2 + d^2}}, \frac{c}{\sqrt{c^2 + d^2}} \right) , \tag{127}$$

where of course $\sqrt{c^2 + d^2} = \sqrt{u}$, in agreement with Eq. (124), and $\Delta_{2:\text{opt}} = (d^2 + c^2)/\sqrt{u} = \sqrt{u}$. All of this is of course exactly equivalent to our initial quaternion 2D limit with the profile matrix

$$M_2 = \begin{bmatrix} d & c \\ c & -d \end{bmatrix} , \tag{128}$$

its leading eigenvalue $\epsilon = \sqrt{c^2 + d^2} = \sqrt{u}$, and the optimal eigenvector (a, b) given in Eq. (123), yielding

$$\Delta_{2:\mathrm{opt}} = \begin{bmatrix} a & b \end{bmatrix} \cdot M_2 \cdot \begin{bmatrix} a \\ b \end{bmatrix} . = \sqrt{u} \tag{129}$$

These results are interesting to study because, despite the complexity of the general solution, the intrinsic algebraic structure of any RMSD problem is entirely characterized by a planar rotation such as that described by Eq. (124).

6. Evaluating the 3D Orientation Frame Solution.

The validity of our approximate chord-measures for determining the optimal global frame rotation can be evaluated by comparing their outcomes to the precise geodesic arc-length measure of Eq. (61). The latter is tricky to optimize, but choosing appropriate techniques, e.g., in the Mathematica `FindMinimum[]` utility, it is possible to determine good numerical solutions without writing custom code; in our experiments, fluctuations due to numerical precision limitations were noticeable, but presumably conventional conditioning techniques, which we have not attempted to explore, could improve that significantly. We employed a collection of 1000 simulated quaternion data sets of length 100 for the reference cases, then imposed a normal distribution of random noise on the reference data, followed by a global rotation of all those noisy data points distributed around 45° to produce a corresponding collection of corresponding quaternion test data sets to be aligned. (Observe that we do *not* expect the optimal rotation angles to match the exact global rotations, though they will be nearby.)

We then collected the optimal quaternions for the following cases:

(a) **Arc-Length (numerical).** This is the "gold standard," modulo the occasional data pair that seems to challenge the numerical stability of the computation (which was to be expected). We obtained the data set (a) of quaternions that numerically minimized the nonlinear geodesic arc-length-squared measure of Eq. (61); this is in principle the best estimate one can possibly get for the optimal quaternion rotations to align a set of 3D test-frame triads with a corresponding set of reference-frame triads. There is no known way to find this set of optimal quaternions using our linear algebra methods.

(b) **Chord-Length (numerical and algebraic).** This approach, designated as the data set (b), is based on the approximation to Eq. (17) illustrated in Fig 1 , replacing the arc-length by the chord-length, which amounts to removing the arccosine and using the effective maximal cosines ($t \to \tilde{t}$) to define the measure. The form given in Eq. (71) is a minimization problem that is exactly the quaternion analog of the RMSD problem definition in Eq. (17) for spatial data, with the additional constraint that all the spatial data must be unit-length 4-vectors (which have only 3 degrees of freedom) instead of arbitrary 3-vectors. In addition, the convergence condition for clustering of the data within the ball should in principle be satisfied for the optimal solution of Eq. (71) to be global; our data simulation pushes these limits, but in practice the convergence is typically satisfied. Just as Eq. (17) and its cross-term form Eq. (18) give exactly the same results for spatial data when the measures are minimized and maximized, respectively, the orientation-problem equations Eq. (71) and Eq. (72) do the same for the quaternion measure. Finally, the two cross-term forms Eq. (73) and Eq. (76) give the same optimal quaternions, with the interesting fact that Eq. (73) yields the optimal quaternion from a linear equation, and Eq. (76) gives an identical result from a quadratic matrix equation that works the same way as the RMSD matrix optimization, except that the symmetric profile matrix is no longer traceless.

Thus there are in fact four ways of looking at the chord-length measure and obtaining exactly the same optimal quaternions, and we have checked these using two numerical optimizations and two algebraic optimizations. These options are:

- **Minimizing Euclidean Chord-Length Squared.** Here we write the chord-approximation to the QFA problem using Eq. (71), which is exactly parallel to the RMSD problem employing Eq. (17), modulo the sign ambiguity issue. We test this by performing a numerical minimization.
- **Maximizing Chord-Length Cross-Term.** Just as the RMSD cross-term maximization problem Eq. (18) is equivalent to the RMSD minimization problem of Eq. (17), we can use maximization of the quaternion cross-term Eq. (72) equivalently with the minimization of the chord-length Eq. (71). We test this by performing a numerical maximization.
- **Linear Reduction of Chord-Length Cross-Term.** Pulling out the linear coefficients of the each quaternion component in Eq. (72) generates Eq. (73), where the 4-vector $V_a(W)$ of Eq. (74) plays the role of the RMSD profile matrix $M_{ab}(E)$ in Eq. (20). Here we test the optimization by algebraically solving the linear expression Eq. (73).
- **Quadratic Equivalent Matrix Form of the Chord-Length Cross-Term.** Finally, there is in fact a maximal matrix eigenvalue problem Eq. (76) that works like Eq. (20) by squaring Eq. (73) to get a matrix problem $q \cdot \Omega \cdot q$ with $\Omega_{ab} = V_a V_b$. Despite the presence of a nonvanishing trace, the maximal quaternion eigenvectors are the same as the other three cases above. This produces the same optimal quaternion solutions as solving the (much, much simpler) linear problem of Eq. (73). This can also be checked algebraically.

(c) $(\mathrm{tr}\,\mathbf{R}(\mathbf{q}) \cdot \mathbf{R}(\mathbf{p}) \cdot \mathbf{R}(\bar{\mathbf{r}}))$ **Chord-Length (algebraic).** Finally, the most rigorous method if consistency of quaternion signs cannot be guaranteed is to use a measure in which algebraic squares occur throughout and enforce rigorous sign-independence. This is our (c) data set. Such measures must of necessity be *quartic* in the quaternion test and reference data, and thus are distinct from the measures of (b) that are *quadratic* in the data elements. This $(\mathrm{tr}\,R(q) \cdot R(p) \cdot R(\bar{r}))$ measure is the form that is most easily integrated into the combined rotational-translational problem treated in the next section, because the combined matrices are both symmetric and traceless like the original RMSD profile matrices. Furthermore, it is obvious from Eq. (80) that this measure is exactly the same as the one obtained from Eq. (72) if we squared *each term in k* before summing the cross-term data elements in option (b). Thus, whichever actual formula we choose, we appear to have exhausted the options for quaternion-sign-independent quartic measures for the orientation data problem.

The task now is simply to evaluate how close the optimal quaternion solutions for the arc-length measure (a) are to the quadratic chord-length measures (b) and the quartic chord-length measures (c). In addition, we would like to know how close the fragile but very elegant quadratic measures (b) are to the rigorously sign-insensitive quartic measures (c); we expect them to be similar, but we do not expect them to be identical.

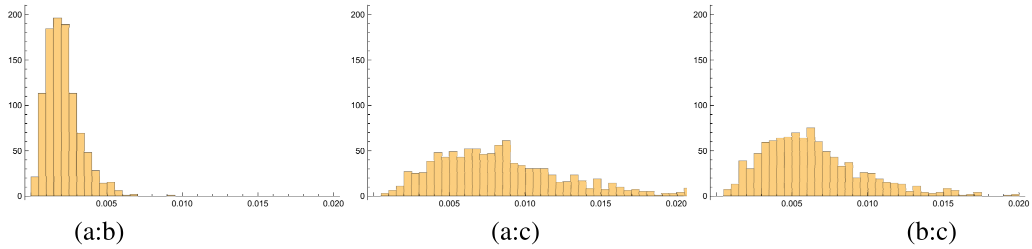

Figure 2
Spectrum in degrees of angular differences between optimal quaternion alignment rotations for quaternion frames. (a:b): (a) vs (b), true arc-length vs approximate quadratic chord-length measure. (a:c): (a) vs (c), true arc-length vs approximate quartic chord-length measure. (b:c): (b) vs (c), approximate quadratic vs approximate quartic chord-length measure.

To quantify the closeness of the measures, we took the magnitude of the inner products between competing optimal quaternions for the same data set, which is essentially a cosine measure, took the arccosines, and converted to degrees.

The results were histogrammed for 1000 random samples consisting of $N = 100$ data points, and are presented in Fig 2. The means and standard deviations of the optimal total rotations relative to the identity frame for the three cases are:

Measure Type	Mean(deg)	Std Dev(deg)
**(a) arc-length**	44.8062	11.2307
**(b) chord quadratic**	44.8063	11.2308
**(c) chord quartic**	44.8065	11.2310

.

One can see that our simulated data set involved a large range of global rotations, and that all three methods produced a set of rotations back to the optimal alignment that are not significantly different statistically. We thus expect very little difference in the histograms of the case-by-case optimal quaternions produced by the three methods. The mean differences illustrated in the Figures are summarized as follows:

Figure:(Pair)	Mean(deg)	Std Dev(deg)
**Figure 2 (a:b)**	0.0021268	0.0011284
**Figure 2 (a:c)**	0.0084807	0.0044809
**Figure 2 (b:c)**	0.0063539	0.0033526

.

We emphasize that these numbers are in degrees for 1000 simulated samples with a distribution of global angles having a standard deviation of 11°. Thus we should have no issues using the chord approximation, though it does seem that the $q \cdot V$ measure is significantly better both in accuracy and simplicity of computation.

7. The 3D Combined Point+Frame Alignment Problem.

The 3D combined alignment problem for both spatial data and orientation-frame data involves a number of issues and subtleties that we were able to treat only superficially in the main text. In this section, we explore various options and evaluate their performance. This is necessary for anyone who might think of trying to attempt a combined alignment problem, so we have attempted to anticipate the questions and alternatives that might be explored and check their properties. The overall result is that it seems difficult to obtain significant additional information from the combined alignment strategies that we examined, so potential exploiters of this paradigm are forewarned.

From the main text, we are in possession of precise alignment procedures for both 3D spatial coordinates and 3D frame triad data (using the exact measure for the former and one of the approximate chord measures for the latter), and thus we can consider the full 6 degree-of-freedom alignment problem for combined data from a single structure. In fact this problem can also be solved in closed algebraic form given the our existing eigensystem formulation of the orientation alignment problem. While there are clearly appropriate domains of this type, e.g., any protein structure in the PDB database can be converted to a list of residue centers and their local frame triads (Hanson & Thakur, 2012), little is known at this time about the potential value of combined alignment. To establish the most complete possible picture, we now proceed to describe the details of our solution to the alignment problem for combined translational and rotational data.

In our treatment, we will assume the Δ_{RRR} measure since its profile matrix is traceless and manifestly independent of the quaternion signs, but there is no obstacle to using $\Delta_{\text{frame-sq}}$ if the data are properly prepared and one prefers

the simpler measure. For notational simplicity, we will let Δ_f stand for whatever orientation frame measure we have chosen, corresponding to Δ_x for the spatial measure, and thus we will denote the combined measure by Δ_{xf}.

The Combined Optimization Measure. A significant aspect of establishing a combined measure including the point measure Δ_x and the frame orientation measure Δ_f is the fact that the measures are *dimensionally incompatible*. We *cannot* directly combine the corresponding data minimization measures $\Delta_x(q_x) = \epsilon_{x:\max}$ and $\Delta_f(q_f) = \epsilon_{f:\max}$ because the spatial measure has dimensions of (length)^2 and the frame measure is essentially a dimensionless trigonometric function (the arc-distance measure produces (radians)^2, which is still incompatible).

While it should be obvious that a combined measure requires an arbitrary, problem-specific, interpolating constant with dimensions of length to produce a compatible measure, there has been some confusion in the literature. These issues are implicitly incorporated, e.g., in the weights α and β in the error function of the dual quaternion approach of (Walker *et al.*, 1991) and explicitly resolved with the introduction of dimensionful constants, e.g., in the molecular entropy work of (Fogolari *et al.*, 2016) [see also (Huggins, 2014)]. Our approach to defining a valid heuristic combined measure has three components:

- **Normalize the Profiles.** The numerical sizes of the maximal eigenvalues of the Δ_x and the Δ_f systems can easily differ by orders of magnitude. Since scaling the profile matrices changes the eigenvalues *but not the eigenvectors*, it is perfectly legitimate to start by dividing the profiles by their maximal eigenvalues before beginning the combined optimization, since this accomplishes the sensible effect of assigning maximal eigenvalues of exactly unity to both of our scaled profile matrices.
- **Interpolate between the Profiles.** To allow an arbitrary sensible weighting distinguishing between a location-dominated measure and an orientation-dominated measure, we simply incorporate a linear interpolation parameter $t \in [0,1]$, with $t = 0$ singling out Δ_x and the pure (unit eigenvalue) location-based RMSD, and $t = 1$ singling out Δ_f and the pure orientation (unit eigenvalue) QFA solution.
- **Scale the Frame Profile.** Finally, we incorporate the mandatory dimensional scaling adjustment by incorporating one additional (nominally dimensional) parameter σ that scales the orientation parameter space described by Δ_f to be more or less important than the "canonical" spatial dimension component Δ_x, which we leave unscaled. That is, with $\sigma = 0$ only the spatial measure survives, with $\sigma = 1$, the normalized measures have equal contributions, and with $\sigma > 1$, the orientation measure dominates (this effectively undoes the original frame profile eigenvalue scaling).

We thus start with a combined spatial-rotational measure of the form

$$\begin{aligned}
\Delta_{\text{initial}} &= (1-t)\sum_{a=1,b=1}^{3} R^{ba}(q)E_{ab} + t\,\sigma \sum_{a=1,b=1}^{3} R^{ba}(q)S_{ab} \\
&= (1-t)\,\mathrm{tr}\,(R(q)\cdot E) + t\,\sigma\,\mathrm{tr}\,(R(q)\cdot S) \\
&= \sum_{a=0,b=0}^{3} q_a\left[(1-t)M_{ab}(E) + t\,\sigma\,U_{ab}(S)\right] q_b \\
&= q\cdot\left[(1-t)M(E) + t\,\sigma\,U(S)\right]\cdot q\ ,
\end{aligned} \tag{130}$$

and then impose the unit-eigenvalue normalization on $M(E)$ and $U(S)$, giving our final measure as

$$\Delta_{xf}(t,\sigma) = q\cdot\left[(1-t)\frac{M(E)}{\epsilon_x} + t\,\sigma\frac{U(S)}{\epsilon_f}\right]\cdot q\ . \tag{131}$$

Because of the dimensional incompatibility of Δ_x and Δ_f, we have to treat the ratio

$$\lambda^2 = \frac{t\sigma}{1-t}$$

as a dimensionful constant, so if t is dimensionless, then σ carries the dimensional scale information.

From the profile matrix of Eq. (131), we now extract our optimal rotation solution using the same equations as always, solving for the maximal eigenvalue and its eigenvector either numerically or algebraically, leading to the equivalent of Eq. (22), as we have solved the standard RMSD maximal eigenvalue problem. The result is a parameterized eigensystem

$$\left.\begin{array}{c} \epsilon_{\mathrm{opt}}(t,\sigma) \\ q_{\mathrm{opt}}(t,\sigma) \end{array}\right\} \qquad (132)$$

yielding the optimal values $R(q_{\mathrm{opt}}(t,\sigma))$, $\Delta_{xf} = \epsilon_{\mathrm{opt}}(t,\sigma)$ based on the data $\{E,S\}$ no matter what we take as the values of the two variables (t,σ).

Properties of the Combined Optimization. Substantially different features arise in the solutions depending on how close the optimal rotations were for the initial, separate, systems Δ_x and Δ_f. We now choose a selection of simulated data sets with the following choices of approximate initial global rotations of the test data sets relative to the reference data:

Table 1
Offsets of sample data for the spatial vs orientation data used in exploring the properties of combined measures.

**DATA ID**	**(Space, Orientation)**	Measured Offset
Data Set 1	$(\mathbf{22}^\circ, -\mathbf{22}^\circ)$	44.60
Data Set 2	$(\mathbf{22}^\circ, -\mathbf{11}^\circ)$	21.98
Data Set 3	$(\mathbf{22}^\circ, \mathbf{0}^\circ)$	11.15
Data Set 4	$(\mathbf{22}^\circ, \mathbf{11}^\circ)$	11.15
Data Set 5	$(\mathbf{22}^\circ, \mathbf{21}^\circ)$	1.20

In Fig 3, we plot the trajectory of the maximal combined similarity measure for Data Set 1 as a function of t, showing the behavior for $\sigma = 1.0, 0.80$, and 1.15. Figure 4 shows a more comprehensive representation of the continuous behavior with σ, and in both figures, we see that the true optima are *at the end points*, $t = 0, 1$, the locations associated with the pure profile eigenvector solutions $q_x(\mathrm{opt})$ and $q_f(\mathrm{opt})$. There is no *better* optimal eigenvector (i.e., global rotation) for any intermediate value of t. In some circumstances, however, it might be argued that it is appropriate to choose the *distinguished value* of t at the minimum of the curve $\Delta_{xf}(t, \sigma = 1)$. As we shall see in a moment, just as in Fig 3 for $\sigma = 1$, this point is generally within a few percent of $t = 0.5$. As the spatial and orientation optima get closer and closer, the curves in t become much flatter and less distinguished, while the variation in σ is qualitatively the same as in Fig 4 .

Finally, we examine one more amusing visualization of the properties of the composite solutions, restricting ourselves to $\sigma = 1$ for simplicity, and examining the “sideways warp” in the quaternion eigenvector $q_{\mathrm{opt}}(t, \sigma = 1)$ in Eq. (132). We examine what happens to the combined similarity measure Eq. (131) if we smoothly interpolate from the identity matrix (that is, the quaternion $q_{\mathrm{ID}} = (1,0,0,0)$) through the optimal solution for each t and beyond the optimum by the same amount, using the *slerp* interpolation defined in Eq. (13), i.e., $q(s) = \mathit{slerp}(q_{\mathrm{ID}}, q_{\mathrm{opt}}(t, \sigma = 1), s)$. Figure 5 shows Data Set 1, with the largest relative spatial vs orientation angular differences, Figure 6 corresponds to the intervening Data Sets 2, 3, 4, and 5, with the Data Set parameters given above in Table 1. Data Set 5 in particular is perhaps the most realistic example, having nearly identical spatial and angular rotations, and we see negligible differences between the spatial and angular structures. These graphics also show how the local, non-optimal, neighboring quaternion values peak in s at the optimal ridge going from $t = 0$ to $t = 1$. The red dot is the maximum of Δ_x at $t = 0$, the green dot is the maximum of Δ_f at $t = 1$, and the blue dot, specific to each data set, is the distinguished point at the *minimum* of $\Delta_{xf}(t, \sigma = 1)$ in t, which for our data sets are always within 1% of $t = 0.5$. We observe that for equal and opposite rotations, the midpoint coincides almost exactly with the identity quaternion that occurs at the left and right boundaries of the plot. In other respects, the data in these figures show that we do not have *maxima* in the middle of

the interpolation in t, but we do have a distinguished value, always very near $t = 0.5$, that could be used as a baseline for a hybrid translational-rotational rotation choice.

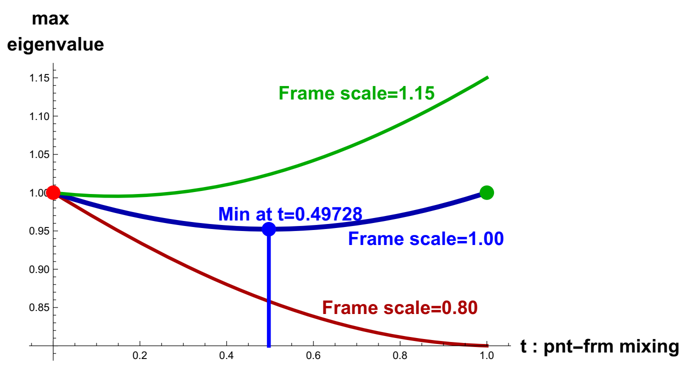

Figure 3
The blue curve is the path of the composite eigenvalue for Data Set 1 (the value of the similarity measure $\Delta_{xf}(t, 1)$) in the interpolation variable t with equally weighted space and orientation data, i.e., $\sigma = 1$. It has maxima only at the "pure" extremes at $t = 0, 1$, but there is a minimum that occurs, for these data, not at $t = 1/2$, but very nearby at $t = 0.49728$. Increasing the influence of the spatial data by taking $\sigma = 0.8$ gives the red curve, and increasing the influence of the orientation data by taking $\sigma = 1.15$ gives the green curve.

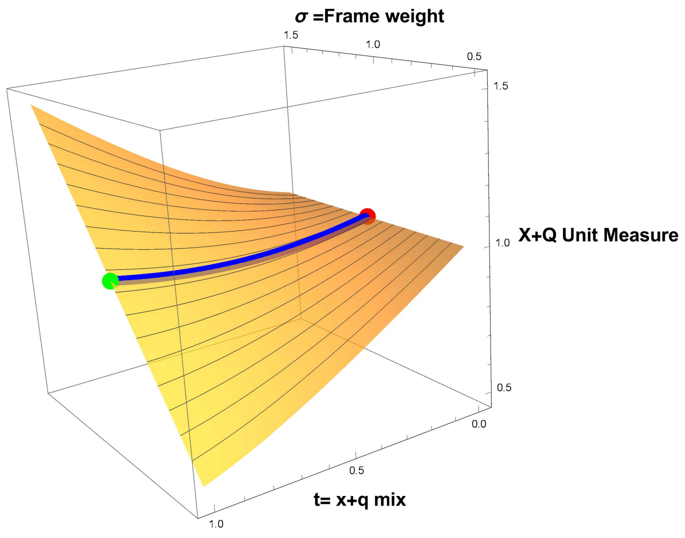

Figure 4
The $\Delta(t, \sigma)$ similarity-measure surface for Data Set 1 as a function of the interpolation parameter t and the relative scaling of the orientation term with σ, with the slightly concave curve at $\sigma = 1$ in the middle. The other data sets look very much like this one.

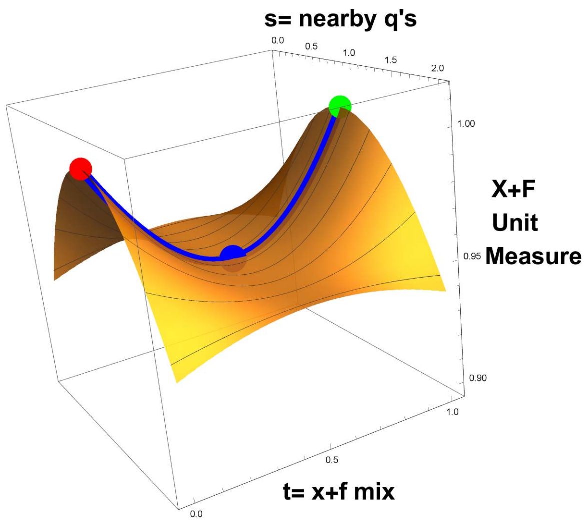

(Set 1)

Figure 5
The $\Delta_{xf}(t, 1)$ similarity-measure surface for Data Set 1, x-angle 22°, f-angle −22°, and fixed $\sigma = 1$ showing the deviation with the quaternion varying perpendicularly around the solution $q(t)$, starting at the identity quaternion at $s = 0$, as a function of the interpolation parameter t. Since $q(t)$ is the maximal eigenvector, all variations in q peak there. Both have distinguished central points at $t \approx 0.5$.

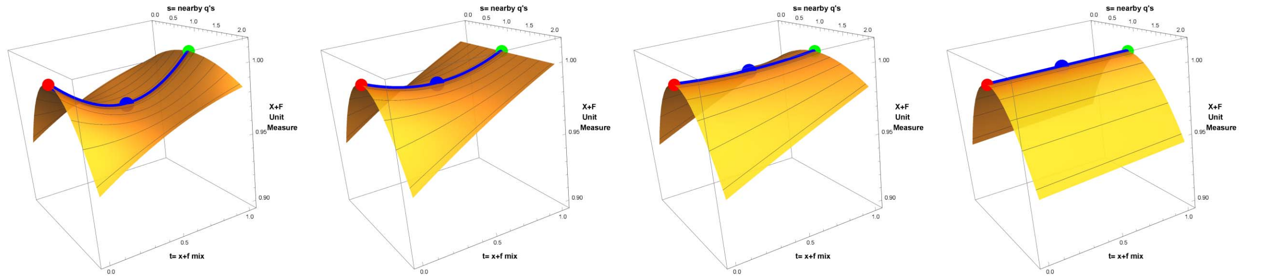

(Set 2) (Set 3) (Set 4) (Set 5)

Figure 6
The $\Delta_{xf}(t, 1)$ similarity-measures with $q(s)$ interpolated from the identity through the optimum for Δ_{xf} and past to the identity-mirror point, for Data Sets 2, 3, 4, and 5, where Data Set 5 has the x-angle and the f-angle only one degree apart, as we might have for real experimental data.

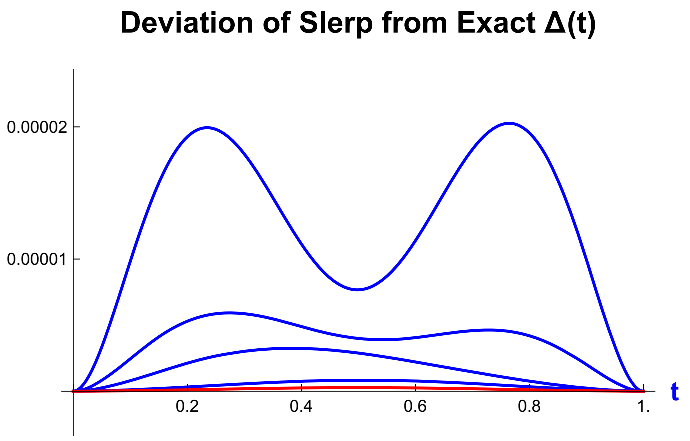

Figure 7
Here we see how close a simple *slerp*(t) between the extremal optimal eigenvectors $q_{\rm opt}(t=0,\sigma=1)=q_x(\rm opt)$ and $q_{\rm opt}(t=1,\sigma=1)=q_f(\rm opt)$ is to the rigorous result where we optimized $q_{\rm opt}(t,\sigma=1)$ *for all t*. The differences are *relative to the unit eigenvalue*, and thus are of order thousandths of a percent, decreasing significantly as the global rotations applied to the space and orientation data approach one another. The largest deviation is for Data Set 1, which interestingly has a third minimum near the center in t; for the highly similar data in Data Set 5, the difference shown in red had to be magnified by 100 even to show up on the graph.

The Simple Approximation. Having now observed that it is possible to construct and solve a rigorous combined RMSD-QFA problem (with the chord-distance approximation in the angular measure), one might ask how that compares to the very simplest idea one might use to interpolate between the measures: what if we take the rigorous combined profile matrix defined by Eq. (131), compared to the *slerp* relating the two optimal eigenvectors of the independent spatial and orientation frame problems, that is

$$q(t) = slerp(q_{x:\rm opt}, q_{f:\rm opt}, t)\ . \tag{133}$$

Given the individual optimal eigenvectors, if we compare this simple $q(t)$ to Eq. (131) for any t (and $\sigma = 1$), we find that the differences are essentially negligible. In Fig 7, we plot the continuous differences of the similarity functions, which we recall are scaled to have a maximal eigenvalue equal to unity. These scaled differences are on the order of one thousandth of a percent or less as the global rotations applied to the spatial and rotational data become close to one another. We conclude that, for all practical purposes, we might as well use Eq. (133) to estimate the combined similarities.

Appendix A

Details of the Algebraic Solutions to the Quartic Eigenvalue Problem

Given the data for the 3D or 4D test and reference structures, we can numerically solve for the maximal eigenvalue of $M_3(E_3)$ and its eigenvector in 3D, or the maximal eigenvalue of $G = M_4{}^{\mathsf{T}}(E_4) \cdot M_4(E_4)$ and the left and right eigenvectors of G in 4D. Alternatively, we can apply the numerical SVD method directly to E_3 or E_4 to determine the optimal rotation matrix.

However, we can also work out the properties of the eigensystems of the various matrices that have come up in our treatment *algebraically*, using classic methods (Abramowitz & Stegun, 1970) for solving quartic polynomial equations for the eigenvalues, to provide deeper insights into the structure of the problem. We now study some features of these results in more detail, and in particular we consider real symmetric matrices, with and without a trace, since essentially every problem we have encountered reduces to finding the maximal eigenvalues of a matrix in that category.

The Eigenvalue Expansions. We begin by writing down the eigenvalue expansion of an arbitrary real 4D matrix M as

$$\det[M - eI_4] = 0 \, , \tag{134}$$

where e denotes a generic eigenvalue and I_4 is the 4D identity matrix. Our task is to express these eigenvalues, particularly the maximal eigenvalue, in terms of the elements of the matrix M, and also to find their eigenvectors.

By expanding Eq. (134) in powers of e, we see how the four eigenvalues $e = \epsilon_{k=1,\ldots,4}$ depend on the known components of the matrix M and correspond to the solutions of the quartic equations that we can express in two useful forms,

$$e^4 + e^3 p_1 + e^2 p_2 + e p_3 + p_4 = 0 \tag{135}$$

$$(e - \epsilon_1)(e - \epsilon_2)(e - \epsilon_3)(e - \epsilon_4) = 0 \, . \tag{136}$$

Here the p_k are homogeneous polynomials of order k that can be expressed alternatively employing elements of M or elements of E for the 3D amd 4D spatial data, or with the corresponding orientation-frame data. At this point we want to be as general as possible, and so we note the form valid for all 4×4 matrices M in the expansion of Eq. (134) and Eq. (135):

$$p_1(M) = -\operatorname{tr}[M] \tag{137}$$

$$p_2(M) = -\frac{1}{2}\operatorname{tr}[M \cdot M] + \frac{1}{2}(\operatorname{tr}[M])^2 \tag{138}$$

$$p_3(M) = -\frac{1}{3}\operatorname{tr}[M \cdot M \cdot M] + \frac{1}{2}\operatorname{tr}[M \cdot M]\operatorname{tr}[M] - \frac{1}{6}(\operatorname{tr}[M])^3 \tag{139}$$

$$\begin{aligned} p_4(M) &= -\frac{1}{4}\operatorname{tr}[M \cdot M \cdot M \cdot M] + \frac{1}{3}\operatorname{tr}[M \cdot M \cdot M]\operatorname{tr}[M] + \frac{1}{8}\operatorname{tr}([M \cdot M])^2 - \frac{1}{4}\operatorname{tr}[M \cdot M](\operatorname{tr}[M])^2 + \frac{1}{24}(\operatorname{tr}[M])^4 \\ &= \det[M] \, . \end{aligned} \tag{140}$$

Remember that for our problem, M is just a real symmetric numerical matrix, and the four expressions $p_k(M)$ are also just a list of real numbers.

Matching the coefficients of powers of e in Eqs. (135) and (136), we can also eliminate e to express the the matrix data expressions p_k in terms of the symmetric polynomials of the eigenvalues ϵ_k as (Abramowitz & Stegun, 1970)

$$\left.\begin{aligned} p_1 &= -\epsilon_1 - \epsilon_2 - \epsilon_3 - \epsilon_4 \\ p_2 &= \epsilon_1\epsilon_2 + \epsilon_1\epsilon_3 + \epsilon_2\epsilon_3 + \epsilon_1\epsilon_4 + \epsilon_2\epsilon_4 + \epsilon_3\epsilon_4 \\ p_3 &= -\epsilon_1\epsilon_2\epsilon_3 - \epsilon_1\epsilon_2\epsilon_4 - \epsilon_1\epsilon_3\epsilon_4 - \epsilon_2\epsilon_3\epsilon_4 \\ p_4 &= \epsilon_1\epsilon_2\epsilon_3\epsilon_4 \end{aligned}\right\} \, . \tag{141}$$

Both Eq. (135) and Eq. (141) can in principle be solved directly for the eigenvalues in terms of the matrix data using the solution of the quartic published by Cardano in 1545 and investigated further by Euler (Euler, 1733; Bell, 1733(2008); Nickalls, 2009) (see also (Abramowitz & Stegun, 1970; Weisstein, 2019; Nickalls, 1993; Wikipedia:Cardano, 2019)). Applying, e.g., the Mathematica function

$$\texttt{Solve[myQuarticEqn[e]} == 0, \texttt{e}, \texttt{Quartics} \rightarrow \texttt{True]} \tag{142}$$

to Eq. (135) immediately returns a usable algebraic formula. However, applying `Solve[ ]` to Eq. (141) is in fact unsuccessful, although invoking `Reduce[pkofepsEqns,` $\{\epsilon_1, \epsilon_2, \epsilon_3, \epsilon_4\}$`, Quartics → True, Cubics → True]` can solve Eq. (141) iteratively and produces the same final answer that we obtain from Eq. (135), as does using a Gröbner basis based on Eq. (141).

In the main paper, we presented a robust algebraic solution that could be evaluated numerically for the quaternion eigenvalues in the special case of a symmetric traceless 4×4 profile matrix $M_3(E_3)$ based on the 3D cross-covariance matrix E_3; we will complete the steps deriving that solution below. But first we will study general 4×4 real matrices, and then specialize to symmetric matrices with and without a trace, as all of our cases of interest are of this type. We note (Golub & van Loan, 1983) that any nonsingular real matrix that can be written in the form $[S^{\mathsf{T}} \cdot S]$ is itself symmetric and has only positive real eigenvalues; in general, the symmetric matrices $[S^{\mathsf{T}} \cdot S]$ and $[S \cdot S^{\mathsf{T}}]$ share one set of eigenvalues, but have distinct eigenvectors. Thus, even if we study only symmetric matrices, we can get significant information about *any* matrix S as long as we can recast our investigation to exploit the associated symmetric matrices $[S^{\mathsf{T}} \cdot S]$ and $[S \cdot S^{\mathsf{T}}]$.

The Basic Structure: Standard Algebraic Solutions for 4D Eigenvalues. When we solve Eq. (135) directly using the textbook quartic solution without explicitly imposing restrictions, we find that the general structure for the eigenvalues $e = \epsilon_k(p_1, p_2, p_3, p_4)$ takes the form

$$\left.\begin{array}{llll} \epsilon_1(p) = -\dfrac{p_1}{4} + F(p) + G_+(p) & & \epsilon_2(p) = -\dfrac{p_1}{4} + F(p) - G_+(p) \\ \epsilon_3(p) = -\dfrac{p_1}{4} - F(p) + G_-(p) & & \epsilon_4(p) = -\dfrac{p_1}{4} - F(p) - G_-(p) \end{array}\right\} . \tag{143}$$

Here $-p_1 = (\epsilon_1 + \epsilon_2 + \epsilon_3 + \epsilon_4)$ is the trace, and we can see that "canonical form" for the quartic Eq. (135), with a missing cubic term in e, results from simply changing variables from $e \rightarrow e + (\epsilon_1 + \epsilon_2 + \epsilon_3 + \epsilon_4)/4$ to effectively add 1/4 of the trace to each eigenvalue. The other two types of terms have the following explicit expressions in terms of the four independent coefficients p_k:

$$\left.\begin{array}{rcl} F(p_1, p_2, p_3, p_4) &=& \sqrt{\dfrac{{p_1}^2}{16} - \dfrac{p_2}{6} + \dfrac{1}{12}\left(\sqrt[3]{a + \sqrt{-b^2}} + \dfrac{r^2}{\sqrt[3]{a + \sqrt{-b^2}}}\right)} \\ G_\pm(p_1, p_2, p_3, p_4) &=& \sqrt{\dfrac{3{p_1}^2}{16} - \dfrac{p_2}{2} - F^2(p) \pm \dfrac{s(p)}{32\, F(p)}} \\ &=& \\ \multicolumn{3}{l}{\sqrt{\dfrac{{p_1}^2}{8} - \dfrac{p_2}{3} - \dfrac{1}{12}\left(\sqrt[3]{a + \sqrt{-b^2}} + \dfrac{r^2}{\sqrt[3]{a + \sqrt{-b^2}}}\right) \pm \dfrac{s(p)}{32\sqrt{\dfrac{{p_1}^2}{16} - \dfrac{p_2}{6} + \dfrac{1}{12}\left(\sqrt[3]{a + \sqrt{-b^2}} + \dfrac{r^2}{\sqrt[3]{a + \sqrt{-b^2}}}\right)}}}} \end{array}\right\} \tag{144}$$

with

$$\left.\begin{array}{rcl} r^2(p_1,p_2,p_3,p_4) &=& {p_2}^2 - 3p_1p_3 + 12p_4 \;=\; \sqrt[3]{a^2+b^2} \\ a(p_1,p_2,p_3,p_4) &=& {p_2}^3 + \frac{9}{2}\left(3{p_3}^2 + 3{p_1}^2 p_4 - p_1p_2p_3 - 8p_2p_4\right) \\ b^2(p_1,p_2,p_3,p_4) &=& r^6(p) - a^2(p) \\ s(p_1,p_2,p_3,p_4) &=& 4p_1p_2 - {p_1}^3 - 8p_3 \end{array}\right\} . \tag{145}$$

For general real matrices, which may have complex conjugate pairs of eigenvalues, the sign of r^2 can play a critical role, so giving in to the temptation to write

$$\frac{r^2}{\sqrt[3]{a+\sqrt{-b^2}}} \to \sqrt[3]{a-\sqrt{-b^2}}$$

leads to anomalies; in addition, b^2 can take on any value, so evaluating this algebraic expression numerically while getting the phases of all the roots right can be problematic. So far as we can confirm, setting aside matrices with individual peculiarities, the formula Eq. (143) yields correct complex eigenvalues for all real matrices, though the numerical order of the eigenvalues can be irregular. When we restrict our attention to real symmetric matrices, a number of special constraints come into play that significantly improve the numerical behavior of the algebraic solutions, as well as allowing us to simplify the algebraic expression itself. The real symmetric matrices are all that concern us for any of the alignment problems.

Symmetric Matrices. We restrict our attention from here on to general symmetric 4×4 real matrices, for which the eigenvalues must be real, and so the roots of the matrix's quartic characteristic polynomial must be real. A critical piece of information comes from the fact that the quartic roots are based on an underlying cube root solution (a careful examination of how this works can be found, for example, in (Coutsias *et al.*, 2004; Coutsias & Wester, 2019; Nickalls, 2009)). As noted. e.g., in (Abramowitz & Stegun, 1970), the roots of this cubic are *real* provided that a particular discriminant is *negative*. This expression takes the form

$${q_{\mathrm{AS}}}^3 + {r_{\mathrm{AS}}}^2 \le 0 ,$$

where $\{\mathrm{AS}\}$ disambiguates the Abramowitz-Stegun variable names, and the relationship to our parameterization in terms of the eigenequation coefficients p_k is simply

$$q_{\mathrm{AS}} = -\frac{1}{9} r^2(p_1,p_2,p_3,p_4) , \qquad r_{\mathrm{AS}} = \frac{1}{27} a(p_1,p_2,p_3,p_4) . \tag{146}$$

Thus we can see from Eq. (145) that

$$b^2(p_1,p_2,p_3,p_4) = r^6(p) - a^2(p) = -9^3\left({q_{\mathrm{AS}}}^3 + {r_{\mathrm{AS}}}^2\right) , \tag{147}$$

and hence for symmetric real matrices we must have $b^2(p) \ge 0$. Therefore for this case we can always write

$$\left(a(p) + \sqrt{-b(p)^2}\right) \longrightarrow (a + \mathrm{i}\, b) , \tag{148}$$

and then we can rephrase our general solution from Eqs. (143), (144), and (145) as

$$\left.\begin{array}{rcl} F(p_1,p_2,p_3,p_4) & = & \sqrt{\dfrac{{p_1}^2}{16} - \dfrac{p_2}{6} + \dfrac{1}{6}\,r(p)\,c(a,b)} \\ G_{\pm}(p_1,p_2,p_3,p_4) & = & \sqrt{\dfrac{{p_1}^2}{8} - \dfrac{p_2}{3} - \dfrac{1}{6}\,r(p)\,c(a,b) \pm \dfrac{s(p)}{32\,\sqrt{\dfrac{{p_1}^2}{16} - \dfrac{p_2}{6} + \dfrac{1}{6}\,r(p)\,c(a,b)}}} \\ & = & \sqrt{\dfrac{3{p_1}^2}{16} - \dfrac{p_2}{2} - F^2(p) \pm \dfrac{s(p)}{32\,F(p)}} \end{array}\right\},\qquad (149)$$

where the cube root terms can now be reduced to real-valued trigonometry:

$$\left.\begin{array}{rcl} r(p)\,c(a,b) & = & r(p)\,\cos\left(\dfrac{\arg\,(a+\mathrm{i}b)}{3}\right) \;=\; \dfrac{1}{2}\left((a+\mathrm{i}\,b)^{1/3} + (a-\mathrm{i}\,b)^{1/3}\right) \\ r^2(p) & = & {p_2}^2 - 3p_1p_3 + 12p_4 \;=\; \sqrt[3]{a^2+b^2} \;=\; (a+\mathrm{i}\,b)^{1/3}(a-\mathrm{i}\,b)^{1/3} \\ r^6(p) & = & a^2(p) + b^2(p) \\ s(p) & = & 4p_1p_2 - {p_1}^3 - 8p_3 \end{array}\right\}.\qquad (150)$$

Alternative Method: The Cube Root Triples Method and Its Properties. Our first general method above corresponds directly to (Abramowitz & Stegun, 1970), and consists of combinations of signs in two blocks of expressions. The second method that we are about to explore uses sums of three expressions in all four eigenvalues, with each term having a square root ambiguity; this is fundamentally Euler's solution, discussed, for example, in (Coutsias *et al.*, 2004; Coutsias & Wester, 2019) and (Nickalls, 2009). The correspondence between this triplet and the four expressions in Eq. (149) is delicate, but deterministic, and we will show the argument leading to the equations we introduced in the main text.

The "Cube Root Triple" method follows from the observation that if we break up the general form of the four quartic eigenvalues into a trace part and a sum of three identical parts whose signs are arranged to be traceless, we find an equation that can be easily solved, and which (under some conditions that we will remove) evaluates numerically to the same eigenvalues as Eq. (149), but can be expressed in terms of a one-line formula for the eigenvalue system. The Ansatz that we start with is the following:

$$\left.\begin{array}{rcl} \epsilon_1 & \stackrel{?}{=} & -\dfrac{p_1}{4} + \sqrt{X} + \sqrt{Y} + \sqrt{Z} \\ \epsilon_2 & \stackrel{?}{=} & -\dfrac{p_1}{4} + \sqrt{X} - \sqrt{Y} - \sqrt{Z} \\ \epsilon_3 & \stackrel{?}{=} & -\dfrac{p_1}{4} - \sqrt{X} + \sqrt{Y} - \sqrt{Z} \\ \epsilon_4 & \stackrel{?}{=} & -\dfrac{p_1}{4} - \sqrt{X} - \sqrt{Y} + \sqrt{Z} \end{array}\right\}.\qquad (151)$$

If we now insert our expressions for $\epsilon_k(p_1,X,Y,Z)$ from Eq. (151) into Eq. (141), we see that the p_k equations are

transformed into a quartic system of equations that can in principle be solved for the components of the eigenvalues,

$$\left.\begin{array}{rcl} p_1 &=& p_1 \\ p_2 &=& \dfrac{3{p_1}^2}{8} - 2\,(X+Y+Z) \\ p_3 &=& \dfrac{{p_1}^3}{16} - 8\,\sqrt{X\,Y\,Z} - p_1(X+Y+Z) \\ p_4 &=& \dfrac{{p_1}^4}{256} + X^2+Y^2+Z^2 - 2\,(YZ+ZX+XY) - p_1\sqrt{X\,Y\,Z} - \dfrac{{p_1}^2}{8}(X+Y+Z) \end{array}\right\} . \qquad (152)$$

While our original equation Eq. (141) does not respond to `Solve`[..., $\{\epsilon_1, \epsilon_2, \epsilon_3, \epsilon_4\}$, ...], and Eq. (152) with $X \to u^2$, $Y \to v^2$, $Z \to w^2$ does not respond to `Solve`[..., {`u`, `v`, `w`}, ...], for some reason Eq. (152) with X, Y, Z as the free variables responds immediately to `Solve[pkEqnList , {X, Y, Z}, Quartics → True ]`, and produces a solution for $X(p)$, $Y(p)$, and $Z(p)$ that we can manipulate into the following form,

$$F_f(p) = \frac{{p_1}^2}{16} - \frac{p_2}{6} - \frac{1}{12}\left(\phi(f)\left(a(p)+\sqrt{-b^2(p)}\right)^{1/3} + \frac{r^2(p)}{\phi(f)\left(a(p)+\sqrt{-b^2(p)}\right)^{1/3}}\right) . \qquad (153)$$

Here $F_f(p)$ with $f = (x, y, z)$ represents $X(p)$, $Y(p)$, or $Z(p)$ corresponding to one of the three values of the cube roots $\phi(f)$ of (-1) given by

$$\phi(x) \;=\; -1\;, \qquad \phi(y) \;=\; \tfrac{1}{2}\left(1+\mathrm{i}\sqrt{3}\right)\;, \qquad \phi(z) \;=\; \tfrac{1}{2}\left(1-\mathrm{i}\sqrt{3}\right)\;, \qquad (154)$$

and the utility functions are defined as above in Eq. (145). Once again, because we have symmetric real matrices with real eigenvalues, we know that the discriminant condition for real solutions requires $b^2(p) \geq 0$, so we can again apply Eq. (148) to transform each $\left(a(p) \pm \sqrt{-b^2(p)}\right)$ term into the form $(a(p) \pm \mathrm{i}\, b(p))$. This time we get a slightly different formula because there is a different $\sqrt[3]{-1}$ phase incorporated into each of the X, Y, Z terms, and we obtain the following intermediate result:

$$\begin{array}{rcl} F_f(p) &=& \dfrac{{p_1}^2}{16} - \dfrac{p_2}{6} - \dfrac{1}{12}\left(\phi(f)\,(a+\mathrm{i}b)^{1/3} + r^2(p)\dfrac{1}{\phi(f)\,(a+\mathrm{i}b)^{1/3}}\right) \\ &=& \dfrac{{p_1}^2}{16} - \dfrac{p_2}{6} - \dfrac{1}{6}\left(\phi(f)(a+\mathrm{i}b)^{1/3} + \overline{\phi(f)}\,(a-\mathrm{i}b)^{1/3}\right) \qquad (155) \\ &=& \dfrac{{p_1}^2}{16} - \dfrac{p_2}{6} - \dfrac{1}{6}\left(\phi(f)(a+\mathrm{i}b)^{1/3} + \overline{\phi(f)(a+\mathrm{i}b)^{1/3}}\right)\;, \qquad (156) \end{array}$$

where $\overline{\phi(f)}$, etc., denotes the complex conjugate, and we took advantage of the relation $\sqrt[3]{a^2+b^2} = r^2(p)$. The cube root terms again reduce to real trigonometry, giving our final result (remember that $\phi(x) = -1$, changing the sign)

$$F_f(p_1, p_2, p_3, p_4) \;=\; \frac{{p_1}^2}{16} - \frac{p_2}{6} + \frac{1}{6}\left(r(p)\cos_f(p)\right)\;, \qquad (157)$$

but now with the direct incorporation of the three phases of $\sqrt[3]{-1}$ from Eq. (154) (see, e.g., (Nickalls, 1993)), we get nothing but phase-shifted real cosines,

$$\cos_X(p) = \cos\left(\frac{\arg(a+\mathrm{i}b)}{3}\right),\; \cos_Y(p) = \cos\left(\frac{\arg(a+\mathrm{i}b)}{3} - \frac{2\pi}{3}\right),\; \cos_Z(p) = \cos\left(\frac{\arg(a+\mathrm{i}b)}{3} + \frac{2\pi}{3}\right) . \qquad (158)$$

The needed subset of the utility functions now reduces to

$$\left.\begin{array}{rcl} r^2(p_1,p_2,p_3,p_4) & = & {p_2}^2 - 3p_1p_3 + 12p_4 \;=\; \sqrt[3]{a^2+b^2} \;=\; (a+\mathrm{i}b)^{1/3}(a-\mathrm{i}b)^{1/3} \\ a(p_1,p_2,p_3,p_4) & = & {p_2}^3 + \frac{9}{2}\left(3{p_3}^2 + 3{p_1}^2p_4 - p_1p_2p_3 - 8p_2p_4\right) \\ b^2(p_1,p_2,p_3,p_4) & = & r^6(p) - a^2(p) \end{array}\right\} . \tag{159}$$

Repairing Anomalies in the Cube Root Triple Form. We are not quite finished, as our X,Y,Z triplets acquire an ambiguity due to possible alternate sign choices when we take the square roots of X,Y,Z to construct the eigenvalues themselves using the Ansatz of Eq. (151). As long as all the terms of one part change sign together, the tracelessness of the X,Y,Z segment of the eigenvalue system is maintained, so there are a number of things that could happen with the signs without invalidating the general properties of Eq. (151). We can check that, with random symmetric matrix data, Eq. (151) with Eq. (157) will yield the correct eigenvalues about half the time, while Eq. (143) with Eq. (149) always works. Inspecting Eq. (149) and Eq. (157) with Eq. (158), we observe that $F(p_1,p_2,p_3,p_4) = \sqrt{F_X(p_1,p_2,p_3,p_4)} = \sqrt{X}$; we can also see that Eq. (149) suggests that a relation of the following form should hold,

$$G_\pm(p_1,p_2,p_3,p_4) \sim \sqrt{Y} \pm \sqrt{Z} \; ,$$

so we can immediately conjecture that something is going wrong with the sign choice of the root $\sqrt{Z}$. It turns out that $G_+(p)$ changes its algebraic structure to essentially that of $G_-(p)$ when the numerator $s(p) = (4p_1p_2 - {p_1}^3 - 8p_3)$ inside the square root in Eq. (149) changes sign. That tells us exactly where there is a discrepancy with the choice $\sqrt{Y} + \sqrt{Z}$. If we define the following sign test,

$$\sigma(p_1,p_2,p_3,p_4) = \mathrm{sign}\left(4p_1p_2 - {p_1}^3 - 8p_3\right) \; , \tag{160}$$

we discover that we can make Eq. (151) agree exactly with the robust $G_\pm(p)$ from Eq. (149) for all the random symmetric numerical matrices we were able to test, provided we make the following simple change to the final form of the X,Y,Z formula for the eigenvalue solutions:

$$\left.\begin{array}{rcl} \epsilon_1 & = & -\frac{p_1}{4} + \sqrt{X} + \sqrt{Y} + \sigma(p)\sqrt{Z} \\ \epsilon_2 & = & -\frac{p_1}{4} + \sqrt{X} - \sqrt{Y} - \sigma(p)\sqrt{Z} \\ \epsilon_3 & = & -\frac{p_1}{4} - \sqrt{X} + \sqrt{Y} - \sigma(p)\sqrt{Z} \\ \epsilon_4 & = & -\frac{p_1}{4} - \sqrt{X} - \sqrt{Y} + \sigma(p)\sqrt{Z} \end{array}\right\} . \tag{161}$$

Algebraic Equivalence of Standard and Cube Root Triple Form. With the benefit of hindsight, we now complete the picture by working out the algebraic properties of Eq. (143) and Eq. (144) that confirm our heuristic derivation of Eq. (161). First, we look back at Eq. (152) and discover that, using the relations for p_2 and p_3, we can incorporate $X+Y+Z = 3{p_1}^3/16 - p_2/2$ into p_3 to get a very suggestive form for our expression $s(p)$ from Eq. (145) in terms of the only square-root ambiguity in our original equations that we used to solve for $(X(p),Y(p),Z(p))$, which is

$$s(p_1,p_2,p_3,p_4) \;=\; 4p_1p_2 - {p_1}^3 - 8p_3 \;=\; 64\sqrt{X(p)Y(p)Z(p)} \; . \tag{162}$$

Already we see that this is potentially nontrivial because $s(p)$ does not have a deterministic sign, but $\sqrt{X(p)Y(p)Z(p)}$ will always be positive unless we have a deterministic reason to choose the negative root.

Next, using Eq. (153), we recast Eq. (144) in a form that uses $F(p) \equiv \sqrt{X(p)} \equiv \sqrt{F_x(p)}$, as well as Eq. (162), to give

$$\left.\begin{aligned} F(p_1,p_2,p_3,p_4) &= \sqrt{X(p_1,p_2,p_3,p_4)} \\ &= \sqrt{\frac{{p_1}^2}{16} - \frac{p_2}{6} + \frac{1}{12}\left(\sqrt[3]{a-\sqrt{-b^2}} + \sqrt[3]{a+\sqrt{-b^2}}\right)} \\ G_\pm(p_1,p_2,p_3,p_4) &= \sqrt{\frac{3{p_1}^2}{16} - \frac{p_2}{2} - F^2(p) \pm \frac{s(p)}{32\,F(p)}} \\ &= \sqrt{A(p_1,p_2,p_3,p_4) \pm B(p_1,p_2,p_3,p_4)} \end{aligned}\right\} \tag{163}$$

where in fact we know a bit about how $B(p)$ should look:

$$B(p) = \frac{s(p)}{32\,\sqrt{X(p)}} \,. \tag{164}$$

Now we solve the equations

$$\sqrt{A(p) \pm B(p)} = \sqrt{Y} \pm \sigma(p)\sqrt{Z} \tag{165}$$

for $A(p)$ and $B(p)$, to discover

$$\begin{aligned} A(p) &= Y(p) + \sigma^2(p)Z(p) \\ &= Y(p) + Z(p) \end{aligned} \tag{166}$$

$$B(p) = 2\sigma(p)\sqrt{Y(p)Z(p)} \,, \tag{167}$$

where we note that these useful relations are nontrivial to discover *directly* from our original expressions for $F(p)$ and $G_\pm(p)$. Finally, using Eq. (164), we conclude that

$$s(p) = 64\,\sigma(p)\sqrt{X(p)Y(p)Z(p)} \,, \tag{168}$$

which confirms that the appearance of

$$\sigma(p) = \mathrm{sign}(s(p)) = \mathrm{sign}(4p_1p_2 - {p_1}^3 - 8p_3) \tag{169}$$

in the (X,Y,Z) expression of Eq. (161) is rigorous and inevitable, as it can be deduced directly from its appearance in $B(p)$.

Alternative Reduction of the Quartic Solution. Perhaps a more explicit way to connect the $(F, G_\pm)$ and (X,Y,Z) forms, and one we might have used from the beginning with further insight, is to observe that $G_\pm$ is actually the square

root of a perfect square,

$$\left.\begin{array}{rcl} G_{\pm} & = & \sqrt{\left(\sqrt{Y} \pm \sigma\sqrt{Z}\right)^2} \\ & = & \sqrt{Y + Z \pm 2\sigma\sqrt{YZ}} \\ & = & \sqrt{Y + Z \pm 2\sigma\dfrac{\sqrt{XYZ}}{\sqrt{X}}} \\ & = & \sqrt{Y + Z \pm 2\sigma\dfrac{64\sqrt{XYZ}}{64\sqrt{X}}} \\ & = & \sqrt{Y + Z \pm 2\sigma\dfrac{|s(p)|}{64\sqrt{X}}} \\ & = & \sqrt{Y + Z \pm \dfrac{s(p)}{32\,\sqrt{X(p)}}} \end{array}\right\} , \tag{170}$$

where we used the fact that $\sigma(p)|s(p)| = s(p)$. As long as the sign with which $G_{\pm}$ enters into the solution is consistent, the alternative overall signs of the radicals in Eq. (170) will be included correctly.

The Traceless Triple Form. The explicitly traceless X, Y, Z triplet form that corresponds to a set of eigenvalues in descending magnitude order that we introduced for the 3D RMSD problem in the main text is is obtained by imposing the traceless condition, $p_1 = 0$, obeyed by the 3D profile matrix $M_3(E_3)$:

$$\left.\begin{array}{rcl} \epsilon_1 & = & +\sqrt{X} + \sqrt{Y} + \sigma(p)\sqrt{Z} \\ \epsilon_2 & = & +\sqrt{X} - \sqrt{Y} - \sigma(p)\sqrt{Z} \\ \epsilon_3 & = & -\sqrt{X} + \sqrt{Y} - \sigma(p)\sqrt{Z} \\ \epsilon_4 & = & -\sqrt{X} - \sqrt{Y} + \sigma(p)\sqrt{Z} \end{array}\right\} . \tag{171}$$

Then Eq. (152) simplifies to

$$p_1 = 0 \tag{172}$$

$$p_2 = -2\,(X + Y + Z) \tag{173}$$

$$p_3 = -8\,\sigma(p)\,\sqrt{X\,Y\,Z} \tag{174}$$

$$p_4 = X^2 + Y^2 + Z^2 - 2\,(YZ + ZX + XY)\ , \tag{175}$$

and the solutions for $X(p)$, $Y(p)$, and $Z(p)$ (and thus for $\epsilon_k(p)$) reduce to:

$$F_f(p_2, p_3, p_4) = +\frac{1}{6}\left(r(p)\cos_f(p) - p_2\right)\ , \tag{176}$$

where the phased cosine terms retain their form

$$\cos_X(p) = \cos\left(\frac{\arg(a + \mathrm{i}b)}{3}\right),\ \cos_Y(p) = \cos\left(\frac{\arg(a + \mathrm{i}b)}{3} - \frac{2\pi}{3}\right),\ \cos_Z(p) = \cos\left(\frac{\arg(a + \mathrm{i}b)}{3} + \frac{2\pi}{3}\right). \tag{177}$$

Here $F_f(p)$ with $f = (x, y, z)$ as always represents $X(p)$, $Y(p)$, or $Z(p)$ and the utility functions simplify to

$$\left.\begin{array}{rcl} \sigma(p_3) & = & \operatorname{sign}(-p_3) \\ r^2(p_2,p_3,p_4) & = & {p_2}^2 + 12p_4 \;=\; \sqrt[3]{a^2+b^2} \;=\; (a+\mathrm{i}b)^{1/3}(a-\mathrm{i}b)^{1/3} \\ a(p_2,p_3,p_4) & = & {p_2}^3 + \frac{9}{2}\left(3{p_3}^2 - 8p_2p_4\right) \\ b^2(p_2,p_3,p_4) & = & r^6(p) - a^2(p) \\ & = & \frac{27}{4}\left(16p_4{p_2}^4 - 4{p_3}^2{p_2}^3 - 128{p_4}^2{p_2}^2 + 144{p_3}^2p_4p_2 - 27{p_3}^4 + 256{p_4}^3\right) \end{array}\right\} . \tag{178}$$

Summary: We therefore have two alternate robust expressions, Eq. (143) with Eq. (149) and Eq. (161) with Eq. (157), for the entire eigenvalue spectrum of any real, symmetric 4×4 matrix M characterized by its four intrinsic eigenequation coefficients (p_1, p_2, p_3, p_4). For the simpler traceless case, we can take advantage of Eq. (171) with Eq. (176).

References

Abramowitz, M. & Stegun, I. (1970). *Handbook of mathematical functions*. New York: Dover Publications Inc. Pages 17–18.

Bar-Itzhack, I. Y. (2000). *Journal of Guidance, Control, and Dynamics*, **23**(6), 1085–1087. *https://arc.aiaa.org/doi/abs/10.2514/2.4654

Bell, J. (1733(2008)). *An English translation of Euler's De formis radicum aequationum cujusque ordinis conjectatio.* . *arXiv:0806.1927v1 [math.HO]. http://arxiv.org/abs/0806.1927

Coutsias, E., Seok, C. & Dill, K. (2004). *J. Comput. Chem.* **25**(15), 1849–1857. *http://www.ncbi.nlm.nih.gov/pubmed/15376254

Coutsias, E. & Wester, M. (2019). *J. Comput. Chem.* **40**(15), 1496–1508. *https://doi.org/10.1002/jcc.25802

Euler, L. (1733). *Commentarii academiae scientiarum imperialis Petropolitianae*, **6**, 216–231. *http://www.eulerarchive.org/pages/E030.html

Fogolari, F., Foumthuim, C. J. D., Fortuna, S., Soler, M. A., Corazza, A. & Esposito, G. (2016). *Journal of Chemical Theory and Computation*, **12**(1), 1–8. PMID: 26605696. *https://doi.org/10.1021/acs.jctc.5b00731

Golub, G. & van Loan, C. (1983). *Matrix Computations*. Baltimore, MD: Johns Hopkins University Press, 1st ed. Sec 12.4.

Hanson, A. J. (2006). *Visualizing Quaternions*. Morgan-Kaufmann/Elsevier.

Hanson, A. J. & Thakur, S. (2012). *Jour. Molec. Graphics and Modelling*, **38**, 256–278.

Hartley, R., Trumpf, J., Dai, Y. & Li, H. (2013). *Int. J. Comput. Vis.* **103**(3), 267–305.

Huggins, D. J. (2014). *J. Chem. Theory Comput.* **10**, 3617–3625.

Huynh, D. Q. (2009). *J. Math. Imaging Vis.* **35**(2), 155–164. *http://dx.doi.org/10.1007/s10851-009-0161-2

Jupp, P. & Kent, J. (1987). *Appl. Statist.* **36**, 34–46.

Markley, F. L., Cheng, Y., Crassidis, J. L. & Oshman, Y. (2007). *J. Guidance, Control, & Dynamics*, **30**(4), 1193–1197.

Moakher, M. (2002). *SIAM J. Matrix Anal. Appl.* **24**(1), 1–16.

Nickalls, R. (1993). *The Mathematical Gazette*, **77**, 354–359. *http://www.jstor.org/stable/3619777

Nickalls, R. (2009). *The Mathematical Gazette*, **93**, 66–75. *htp://www.nickalls.org/dick/papers/maths/quartic2009.pdf)

Schönemann, P. (1966). *Psychometrika*, **31**, 1–10.

Shepperd, S. W. (1978). *Journal of Guidance and Control*, **1**(3), 223–224.

Shoemake, K. (1985). In *Computer Graphics*, vol. 19, pp. 245–254. Proceedings of SIGGRAPH 1985.

Shuster, M. D. & Natanson, G. A. (1993). *The Journal of the Astronautical Sciences*, **41**(4), 545–556.

Walker, M. W., Shao, L. & Volz, R. A. (1991). *CVGIP: Image Underst.* **54**(3), 358–367. *https://doi.org/10.1016/1049-9660(91)90036-O

Weisstein, E. W., (2019). Quartic equation. `http://mathworld.wolfram.com/QuarticEquation.html`. [Online; accessed 12-May-2019].

Wikipedia:Cardano, (2019). Ars Magna (Gerolamo Cardano) — Wikipedia, the free encyclopedia. `http://en.wikipedia.org/w/index.php?title=Ars\%20Magna\%20(Gerolamo\%20Cardano)&oldid=873028064`. [Online; accessed 15-May-2019].
